# Effects of Hydrogen Bonding Solvation by Diverse Fluorinated Bulky Alcohols on the Reaction Rate and Selectivity in Crown Ether Mediated Nucleophilic Fluorination in an Aprotic Solvent

**DOI:** 10.1021/acsorginorgau.4c00081

**Published:** 2024-11-28

**Authors:** Eloah P. Ávila, Mauro V. de Almeida, Marcelo S. Valle, Josefredo R. Pliego

**Affiliations:** a Chemistry Department, Federal University of Juiz de Fora, Cidade Universitaria, São Pedro, Juiz de Fora, Minas Gerais 36036-900, Brazil; b Departamento de Ciências Naturais, Universidade Federal de São João del-Rei, São João del-Rei, MG 36301-160, Brazil

**Keywords:** nucleophilic fluorination, preferential solvation, phase transfer catalysis, solvent effects, theoretical calculations, ωB97M-V

## Abstract

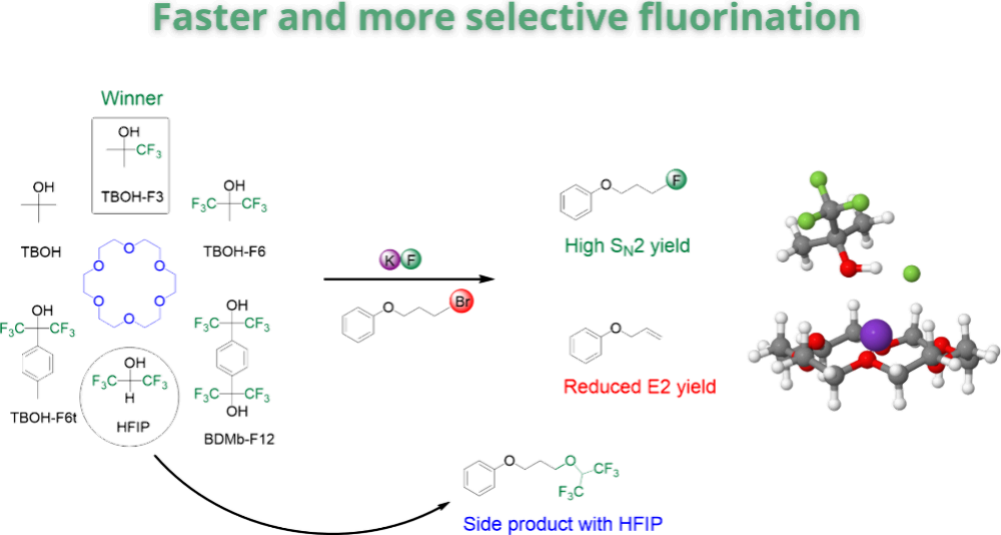

Solvent effects play a critical role in ionic chemical reactions and have been a research topic for a long time. The solvent molecules in the first solvation shell of the solute are the most important solvating species. Consequently, manipulation of the structure of this shell can be used to control the reactivity and selectivity of ionic reactions. In this work, we report extensive experimental and insightful computational studies of the effects of adding diverse fluorinated bulky alcohols with different solvation abilities to the fluorination reaction of alkyl bromides with potassium fluoride promoted by 18-crown-6. We found that adding a stoichiometric amount of these alcohols to the acetonitrile solution has an important effect on the kinetics and selectivity. The most effective alcohol was 2-trifluoromethyl-2-propanol (TBOH-F3), and the use of 3 equiv of this alcohol to fluorinate a primary alkyl bromide led to a 78% fluorination yield in just 6 h of reaction time at a mild temperature of 82 °C, with 8% of E2 yield. The more challenging secondary alkyl bromide substrate obtained 44% fluorination yield and 56% E2 yield at 18 h of reaction time. More fluorinated alcohols with six or more fluorine atoms have resulted in relatively acidic alcohols, leading to large amounts of the corresponding ethers of these alcohols as side products. The widely used hexafluoroisopropanol (HFIP) was the least effective one for monofluorination, indicating that both acidity and bulkiness are important features of the alcohols for promoting fluorination using KF salt. Nevertheless, the ether of HFIP can be easily formed with the substrate, generating a highly fluorinated ether product. Theoretical calculations predict Δ*G*^‡^ in close agreement with the experiments and explain the higher selectivity induced by the fluorinated bulky alcohols in relation to the use of crown ether alone.

## 1 Introduction

The development of nucleophilic fluorination methods of organic compounds has gained an exciting advancement in the past two decades.^1−3^ These developments include stoichiometric reagents,^4−12^ use of crown ether and derivatives,^13−22^ special solvents,^23^ ionic liquids,^24,25^ and catalytic processes based on transition metals^26−31^ and phase transfer catalysis.^32,33^ In the first case, new and more efficient reactants for deoxyfluorination based on S–F bond compounds such as PyFluor^8^ have made this route more attractive for aliphatic fluorination. In addition, safer reactants based on C–F bond compounds such as AlkylFluor^10^ and PhenoFluor^34^ were also developed. Another class of reactants is the tetraalkylammonium fluoride compounds.^4−7^ Although useful for some S_N_Ar reactions of activated substrates, these reactants lead to too much elimination products in the case of S_N_2 fluorination. More selective fluorination processes can be achieved with difluorosilicate reagents, such as tetrabutylammonium difluorotriphenylsilicate (TBAT) and derivatives.^12,35−37^ Nevertheless, even these reagents do not provide enough selectivity for the challenging secondary alkyl bromide substrates. Since new drugs containing CHF and CH_2_F groups have been reported,^38−41^ new developments in aliphatic fluorination are highly desirable. Thus, for the formation of the C(sp^3^)-F bond, we can highlight the use of KF salt as an alternative for aliphatic nucleophilic fluorination because it is a cheap, widely available, and greener fluorine source. However, its low solubility in organic solvents leads to low yield or even no reaction at all.^23,42^ The use of crown ether as a phase transfer catalyst, first reported in the 1970s,^43^ and the design of new macrocycle compounds along the past decade^13−15,18,19^ have led to an important advance in the area of nucleophilic fluorination. It is worth observing that these advances are not limited to the development of synthetic methods. The use of a structured environment around the fluoride ion for controlling its reactivity and selectivity has opened a new area of investigation beyond a simple solvent effect: the rational use of intermolecular forces for controlling ionic reactivity.^44−48^

**Figure 1:**
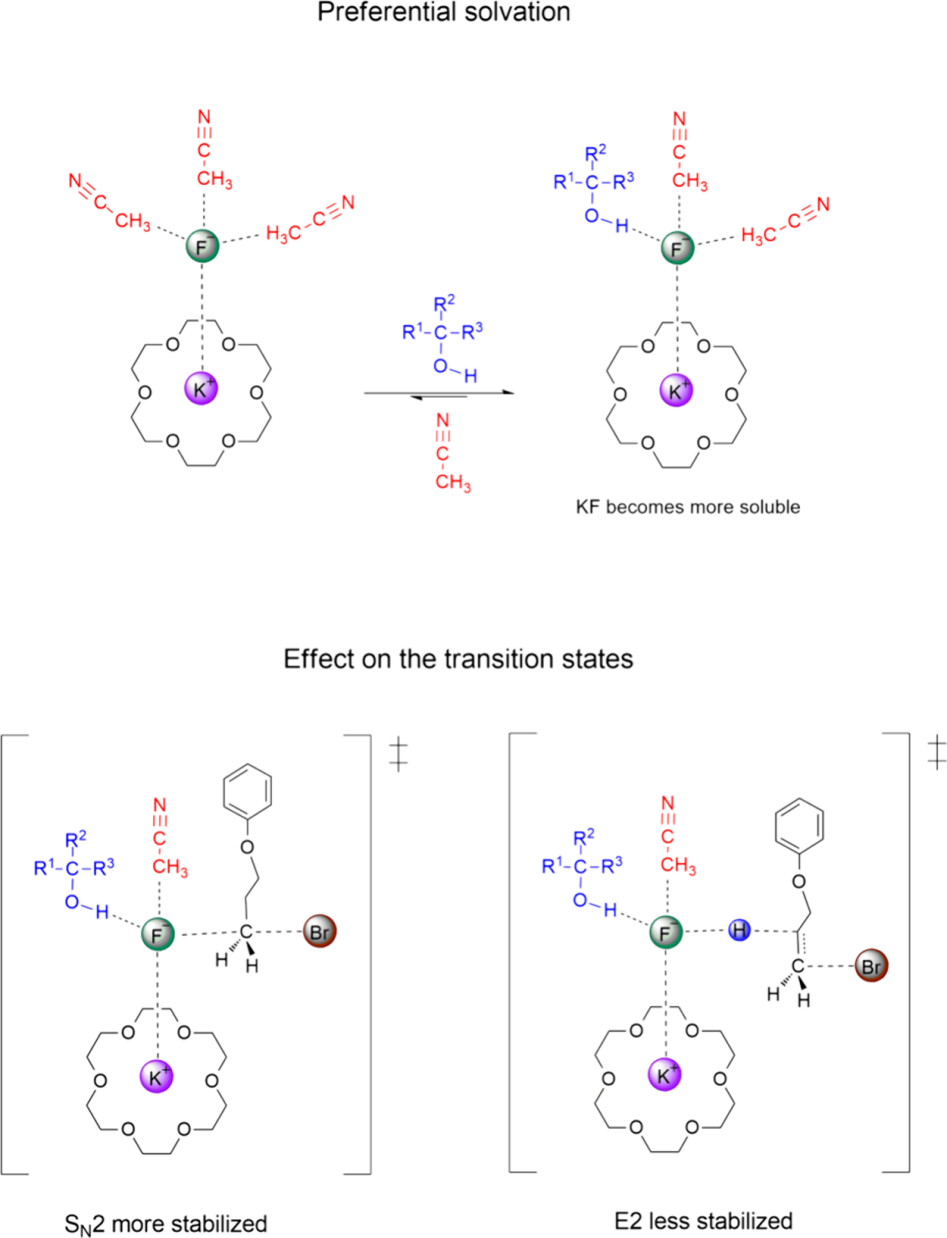
Preferential solvation of the fluoride ion resulting from the added alcohol to the acetonitrile solution having the KF(18-crown-6) complex and effect on the S_N_2/E2 selectivity.

The mechanism in which crown ethers work for catalyzing nucleophilic fluorination with KF is via coordination with the potassium ion of this salt, forming a soluble ion pair, which reacts with the substrate.^17,21^ In aprotic solvents as acetonitrile, the fluoride ion in close contact with its counterion is weakly solvated by the CH_3_ groups,^17,49^ resulting in a reactive and low selective fluoride ion. Consequently, crown ether catalyzed/mediated fluorination can lead to a substantial side E2 reaction, especially for secondary substrates.^43^ Aiming to overcome this problem, hydrogen bond donor species such as bulky alcohols can be added to the reaction medium.^17^ Because these alcohols interact more with the fluoride ion via hydrogen bonding than with the acetonitrile molecules, they can coordinate to the fluoride ion,^50^ replacing the acetonitrile solvation shell and creating a preferential solvation by the alcohol (Figure 1).^17^ This equilibrium has two consequences: First, it increases the solubility of the KF salt due to the increased solvation of the fluoride ion. Second, the fluoride ion becomes less basic due to hydrogen bonding, and the product of the S_N_2 reaction is more favorable than E2, enhancing the selectivity (Figure 1).^51,52^ Based on this effect, a large diversity of hydrogen bond donor species can be used, resulting in different performances.

**Scheme 1:**
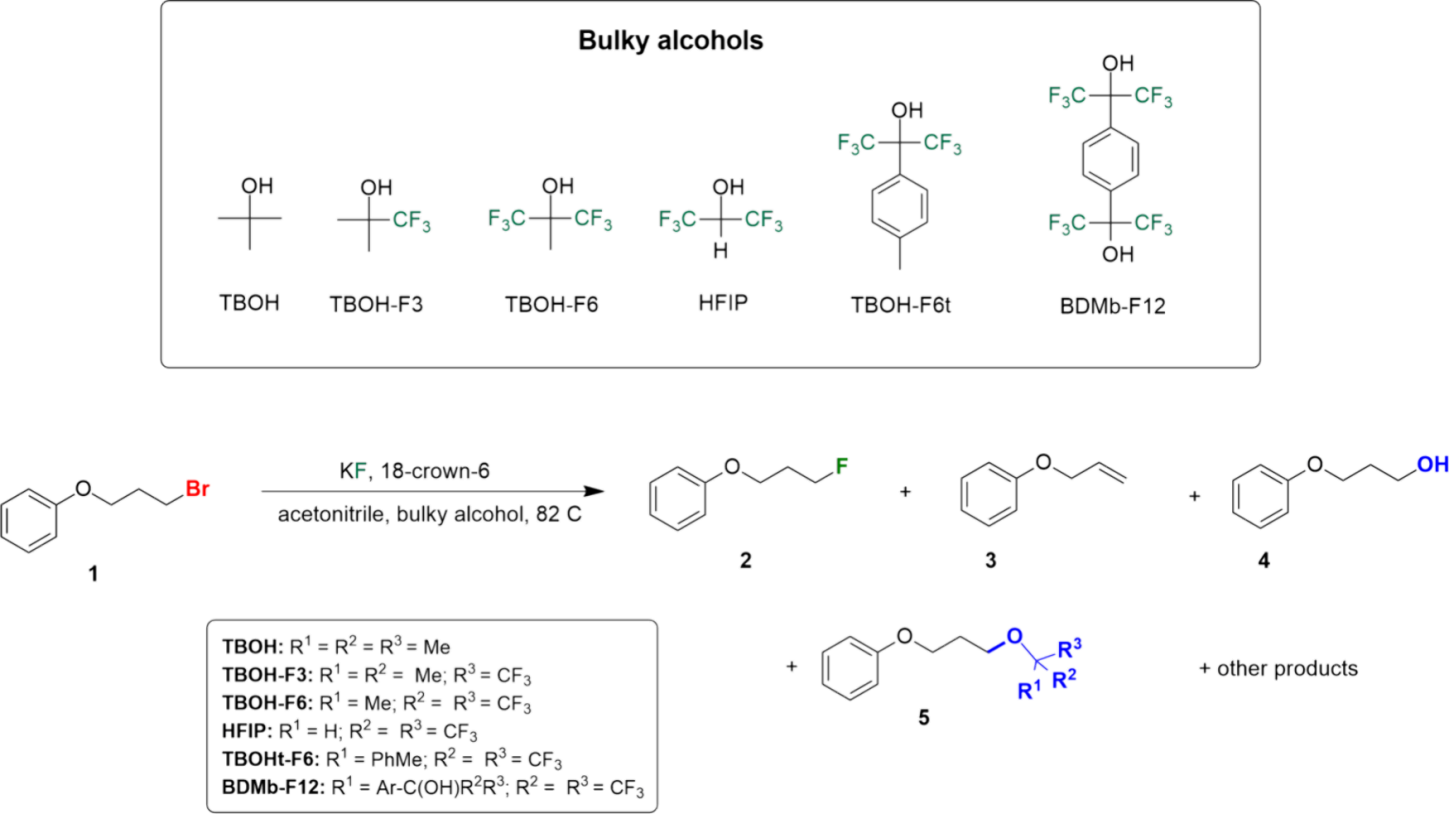
Reaction System Investigated in This Work Using Diverse Bulky Alcohols

The mechanism in Figure 1 has been recently explored in theoretical studies and very limited experiments.^16,17,53^ These studies have suggested that bulk alcohols can accelerate the reaction rate and induce more selective fluorination of a reference primary alkyl bromide substrate. Nevertheless, these limited experimental studies correspond to results of the reaction mixture after a long reaction time (24 h) with *tert-*butyl alcohol (TBOH), 1,4-bis(2-hydroxy-2-propyl)benzene (BDMb), and 1,1,1,3,3,3-hexafluoro-2-methylpropan-2-ol (TBOH-F6) alcohols. A wider experimental investigation of this problem, following the reaction kinetics of the reaction mixture and the formation of unexpected side products, as well as an extended analysis for diverse fluorinated bulky alcohols, has not been reported yet. Thus, this work has presented an unprecedented experimental study of the reaction system of Scheme 1, involving a wide diversity of bulky alcohols, aimed to provide a substantial advance to our understanding of these reaction systems. We used ^1^H NMR, mass spectrometry, and more reliable theoretical calculations than previous reports. The concentrations of the crown ether and the alcohols have also been varied aimed to analyze their effect on the reaction rate and selectivity.

## 2 Methodology

### 2.1 Theoretical Calculations

The reaction system presented in Scheme 1 was investigated by high-level theoretical calculations, considering the TBOH-F3 and TBOH-F6 alcohols. All the geometry optimization was done using the X3LYP functional^54^ in conjunction with the ma-def2-SVP basis set.^55,56^ The solvent effect (acetonitrile) was also included in the optimization via the CPCM method.^57−59^ The vdW surface and Gaussian charge scheme were utilized in these CPCM optimizations to generate a smoother potential of mean force surface.^60^ This is an important issue because the present system has intermolecular complexes with low vibrational modes. Following the optimizations, harmonic frequency calculations were performed to determine the thermodynamic properties. Because low vibrational modes can lead to substantial error in the free energy, we used the quasi-harmonic approximation where the low vibrational modes are transformed to rotational motions, as proposed by Grimme.^61^ However, we did the transformation of both enthalpy and entropy of low vibrational modes to free rotation because this scheme is more consistent and leads to better agreement with experimental data of clustering free energy.^62^ Thus, the free energy contribution for each vibrational mode *n* with frequency ν is given by the following equation:
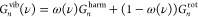
1where the first term on the right side is the harmonic oscillator free energy and the second term is the free rotor free energy. The ω(ν) damping function used to switch these functions is
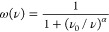
2with ν_0_ = 100 cm^–1^ and α = 4.

To obtain more reliable electronic energies, single-point energy calculations were done using the accurate ωB97M-V functional^63,64^ and the extended ma-def2-TZVPP basis set.^55,56^ In this composite scheme, the solvation free energy was also determined by single-point calculation using the CPCM/X3LYP/ma-def2-SVP level with atomic radii defined in a recent report for acetonitrile solvent.^65^ The most realistic SES surface was used in these calculations, and the nonelectrostatic contribution was calculated using an area-dependent term (molecular SES surface) as recently reported.^65^ This solvation method is more reliable than the SMD model^66^ for the present problem because the atomic cavity of the bromine atom defined by SMD leads to a large error in the solvation free energy.^65^ This point is critical to obtaining an accurate free energy profile.

The p*K*_a_ in acetonitrile solvent of the alcohols in Scheme 1 was also evaluated, aimed at better understanding of the formation of the observed ether products. Because the solvation model used in this study predicts solvation free energy values for anions in acetonitrile solvent in close agreement with the experimental data,^65^ we used the direct method to calculate p*K*_a_ values^67,68^ based on the reaction
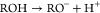
3and the p*K*_a_ was calculated by
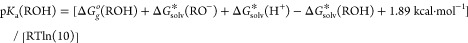
4where we considered that Δ*G*_solv_^*^(H^+^) = −253.2 kcal·mol^–1^ based on a free energy scale^69^ used in the parametrization of the CPCM method.^65^

All the electronic structure calculations were done with the ORCA 5.0.3 program,^70,71^ and the calculation of the thermodynamic properties was done with a Python script written in our group using the data generated by the ORCA calculations.

### 2.2 Experimental Section

All of the reagents were obtained commercially and used without further purification. The experimental procedure to carry out the reactions presented in Scheme 1 is described in the SI file. Thin layer chromatography was performed on TLC plates (silica gel 60 F254) and visualized by employing a UV lamp. Yields refer to chromatographically purified and spectroscopically pure compounds. The ^1^H and ^13^C NMR spectra were recorded at 500 and 125 MHz, respectively, on a Bruker Avance III 500 MHz. Chemical shifts for ^1^H and ^13^C NMR were reported as δ (parts per million (ppm)) relative to the signals of DMSO-*d*_6_ at 2.50 ppm (quintet) and 39 ppm (septet) and CDCl_3_ at 7.26 ppm (singlet) and 77 ppm (triplet). Tetramethylsilane (TMS) was established as an internal reference. NMR chemical shifts are reported employing the following peak abbreviation pattern: br, broad; s, singlet; d, doublet; dd, double doublet; t, triplet; dt, double triplet; q, quartet; dq; double of quartets; and m, multiplet. Infrared experiments were acquired on a Bruker ALPHA FTIR MB102 Spectrometer. High-resolution mass spectra (HRMS) were recorded on a Triple Quad instrument with the accelerator TOF analyzer. Electrospray ionization mass spectrometry ESI-MS(/MS) measurements were performed in the positive and negative ion modes and within the *m*/*z* 50–1000 range.

## 3 Results and Discussion

### 3.1 Acidity of Bulky Alcohols in Acetonitrile

To understand the acid–base equilibria involving bulky alcohols and the fluoride ion in acetonitrile, we calculated their p*K*_a_ values. This is an important issue because the fluoride ion can work as a base in acetonitrile, leading to deprotonation of the alcohol followed by the reaction of the alkoxide with the alkyl bromide. The results are listed in Table 1. The calculated p*K*_a_ of HF is 19.5, which can be compared with the p*K*_a_ of *tert-*butyl alcohol of 42.9. Thus, deprotonation of *tert-*butyl alcohol should be very difficult. For comparison, we also calculated the p*K*_a_ of water molecules in acetonitrile, obtaining a value of 41.9. It is worth observing that water is usually present in the reaction medium, leading to alcohol products. The present calculations indicate that the direct deprotonation of water by the solvated fluoride ion in acetonitrile is very unfavorable. In the sequence, the trifluorinated *tert-*butyl alcohol (TBOH-F3) has a substantial decrease in its p*K*_a_ value to 35.0. Nevertheless, its deprotonation remains difficult.

**Table 1:** Calculated p*K*_a_ Values in Acetonitrile Solution^a^

	ωB97M-V^b^	ΔΔ*G*_solv_^c^	Δ*G*_g_^o^^d^	Δ*G*_sol_*^e^	p*K*_a_^f^	p*K*_a_ (exp)^g^
HF → F^–^ + H^+^	372.46	–337.26	361.93	26.55	19.5	
H_2_O → OH^–^ + H^+^	394.76	–326.25	381.54	57.18	41.9	
TBOH → TBO^–^ + H^+^	383.15	–311.64	368.31	58.57	42.9	
TBOH-F3 → TBO-F3^–^ + H^+^	367.72	–306.98	352.90	47.80	35.0	
TBOH-F6 → TBO-F6^–^ + H^+^	352.40	–303.74	337.58	35.73	26.2	
HFIP → HFIP^–^ + H^+^	350.36	–303.09	335.52	34.32	25.2	
TBOH-F6t → TBO-F6t^–^ + H^+^	347.40	–301.23	332.54	33.20	24.3	
BDMb-F12 → BDMb-F12^–^ + H^+^	341.92	–297.13	327.24	32.00	23.5	
TBOH-F9 → TBO-F9^–^ + H^+^	335.30	–298.94	320.46	23.40	17.2	20.5

a Thermochemical data in units of kcal mol^–1^, 25 °C.

b Electronic energies using the ma-def2-TZVPP basis set.

c Variation of the solvation free energy.

d Gas-phase free energy at 1 atm.

e Solution phase free energy using 1 mol L^–1^ standard state.

f Theoretical p*K*_a_ values.

g Experimental data from ref (72).

With the substitution of more hydrogen atoms by fluorine, forming TBOH-F6, another substantial decrease of the p*K*_a_ is predicted, reducing to 26.2. This lower value suggests an easier equilibrium of alcohol deprotonation with a higher possibility of the formation of the corresponding ether. Other hexafluorinated alcohols such as HFIP and TBOH-F6t have even lower p*K*_a_ values of 25.2 and 24.3, respectively, suggesting that these alcohols are more prone to deprotonate. The diol BDMb-F12 is even more acidic, with a calculated p*K*_a_ value of 23.5. This was the most acidic alcohol experimentally investigated in this work. The -last alcohol analyzed was TBOH-F9, with a p*K*_a_ value calculated to be 17.2, below that of the HF. Thus, this alcohol was not used in the experiments. For comparison, the experimental value of the p*K*_a_ of this alcohol was reported to be 20.5,^72^ a deviation of 3.3 p*K*_a_ units from our calculations.

### 3.2 Theoretical Free Energy Profile of the Reaction System

**Figure 2:**
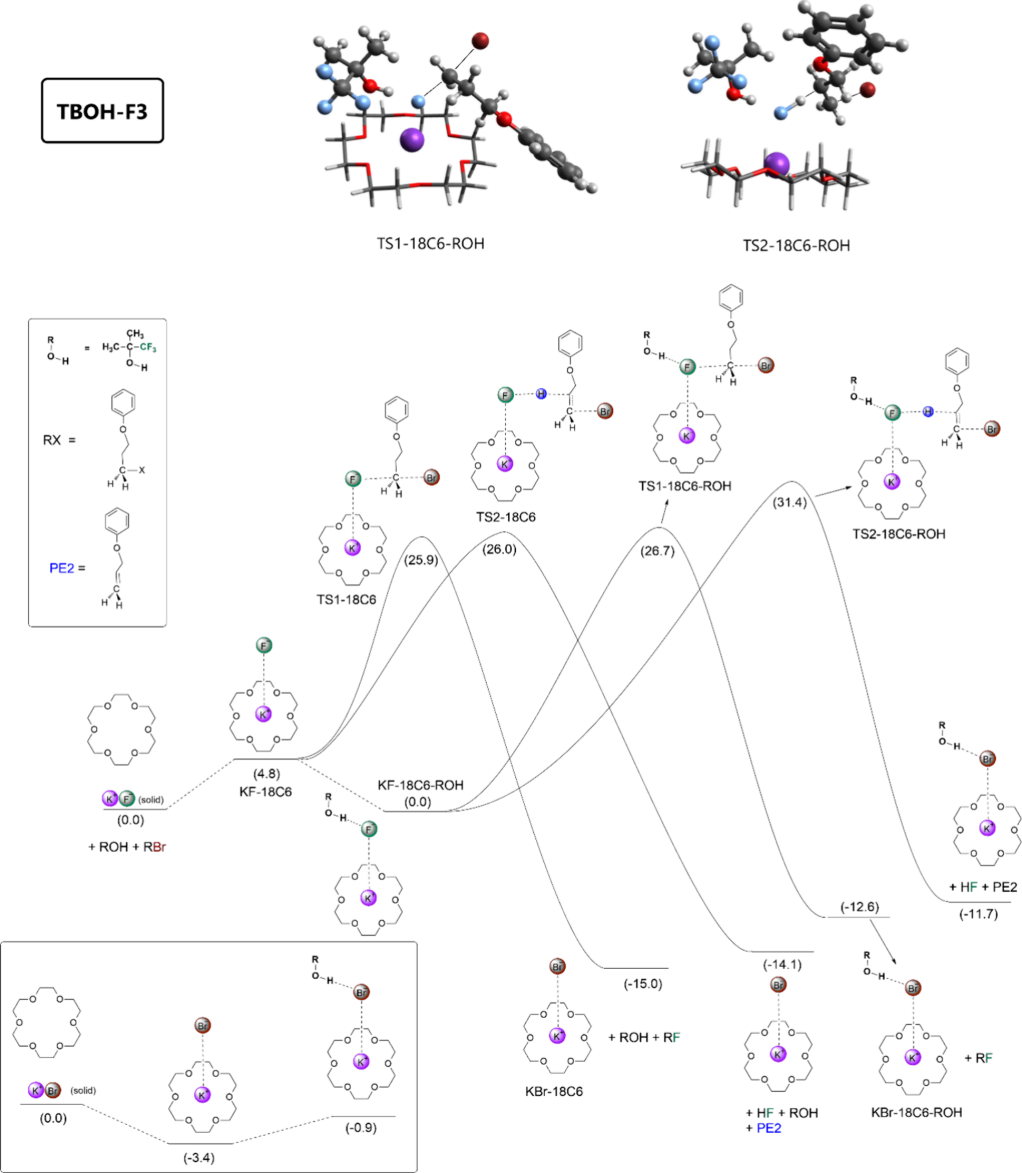
Free energy profile calculated for nucleophilic fluorination of the substrate mediated by crown ether and TBOH-F3. Units are in kcal mol^–1^, 25 °C, 1 mol L^–1^ standard state.

**Figure 3:**
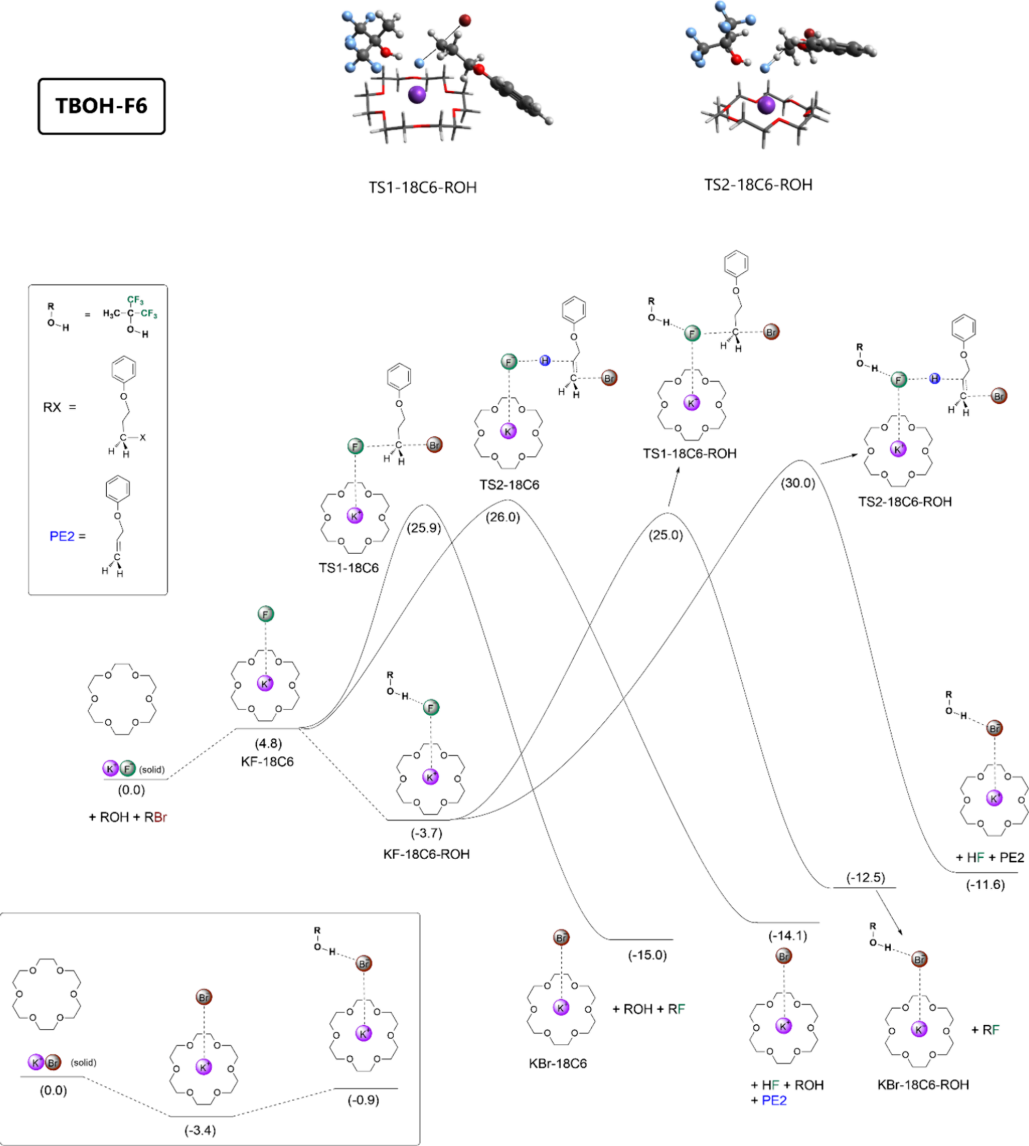
Free energy profile calculated for nucleophilic fluorination of the substrate mediated by crown ether and TBOH-F6. Units in kcal mol^–1^, 25 °C, 1 mol L^–1^ standard state.

We have done accurate theoretical calculations for the reaction system using TBOH-F3 and TBOH-F6 alcohols, which are detailed in the theoretical methods section. The corresponding free energy profiles are presented in Figures 2 and 3, respectively. For the TBOH-F3 alcohol in Figure 2, we can see that the solubilization of the KF salt by 18-crown-6 (KF-18C6 complex) is positive by 4.8 kcal mol^–1^, meaning that a small part of the KF is solubilized by 18-crown-6, in agreement with experimental data.^73^ The effective free energy barriers for the S_N_2 and E2 steps involving KF-18C6 are 25.9 and 26.0 kcal mol^–1^, respectively. These calculations suggest a very high competition between fluorination and elimination reactions for the true substrate, which corresponds to full anhydrous conditions. In the real system, water is present in the medium in variable concentration, which can retard the reaction and increase the S_N_2/E2 product ratio. Another important aspect of this reaction is the fact that the KBr product can be fully solubilized by 18-crown-6, with the result of the KBr becoming bound to the crown ether. Thus, as the reaction advances, less crown ether becomes available for promoting the reaction, and this fact leads to the reduced catalytic ability of the crown ether. In fact, adding the free energy cost to release the crown ether from KBr-18C6 to the free energy barrier of the S_N_2 reaction leads to a final barrier of 29.3 kcal mol^–1^. Thus, a stoichiometric or even larger stoichiometric amount of crown ether would be needed to observe fast reaction rates in acetonitrile reflux (close to 82 °C).

The TBOH-F3 alcohol added to the reaction medium leads to the formation of the KF-18C6-ROH complex, with the Δ*G* for the process calculated to be −4.8 kcal mol^–1^. Thus, the free energy of this complex in relation to the initial reactants is 0.0 kcal mol^–1^, indicating an important ability of this alcohol to increase the solubility of KF. From this reference complex, the Δ*G*^‡^ barriers for the S_N_2 and E2 pathways are 26.7 and 31.4 kcal mol^–1^, respectively. Then, the reactivity promoted by 18-crown-6 alone or in combination with TBOH-F3 is very close for the S_N_2 pathway but becomes much less favorable for E2, with a difference in Δ*G*^‡^ of 4.7 kcal mol^–1^. Therefore, the calculations indicate that the TBOH-F3 alcohol should induce a substantial increase in selectivity provided that a small concentration of KF-18C6 stays in the solution phase, reducing the side E2 pathway via TS2-18C6. In addition, the reaction rate should remain relatively fast, and the problem of KBr bound to the 18-crown-6 must continue to retard the reaction for substoichiometric use of 18-crown-6. The KBr-18C6-ROH complex is not stable, and alcohol should be released to the solution. In summary, the calculations predict that an overstoichiometric addition of TBOH-F3 to the reaction system must induce a substantial increase in the S_N_2/E2 product ratio.

Another alcohol investigated was TBOH-F6, and the free energy profile is presented in Figure 3. As expected, the TBOH-F6 alcohol interacts more with KF-18C6 than does TBOH-F3, with a Δ*G* for the complexation in the solution phase of −8.5 kcal mol^–1^, resulting in a free energy of −3.7 kcal mol^–1^ in the diagram. Thus, this alcohol has an improved ability to solubilize KF in the KF-18C6 complex. However, the Δ*G*^‡^ values for S_N_2 and E2 reactions from these reference complexes are increased to 28.7 and 33.7 kcal mol^–1^, respectively. Observing these numbers, the reaction rate should become lower than using TBOH-F3, with a small increase in the selectivity, with a difference in Δ*G*^‡^ for S_N_2 and E2 reactions amounting to 5.0 kcal mol^–1^. However, there is an interesting difference in the free energy profile, which could increase the reaction rate when using a smaller concentration of the crown ether: the very favorable free energy for solubilization of KF (−3.7 kcal mol^–1^) can overcome the free energy cost to release the KBr from KBr-18C6 (3.4 kcal mol^–1^). Thus, a different kinetic behavior should be expected with the addition of this alcohol, with a lower initial reaction rate. Furthermore, the addition of this alcohol should result in a kinetics less sensible to the use of a lower concentration of the crown ether.

### 3.3 Experiments on the Effect of the 18-Crown-6 on the Reaction Rate and Selectivity

Our computed free energy profile (Figure 2) points out that in the beginning of the reaction, the rate law is given by
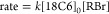
5with an approximated integrated rate law given by
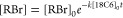
6

**Figure 4:**
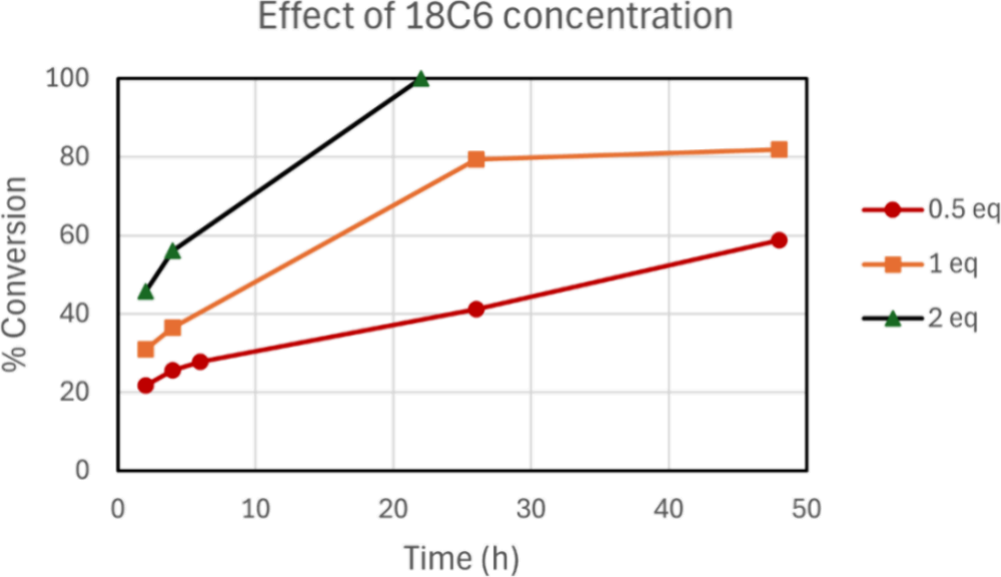
Kinetics of the conversion of substrate **1** at different 18-crown-6 concentrations. Conditions: 1 mmol of substrate **1**, 2 mmol of KF, 4.0 mL of acetonitrile, 82 °C.

Thus, the reaction rate should increase with the concentration of the crown ether and of the substrate. However, as the reaction advances, the resulting KBr acts as an inhibitor of the reaction, binding to the 18-crown-6, and the reaction rate should be considerably retarded in substoichiometric concentration of the 18-crown-6. The experimental results are presented in Figure 4 using 0.5, 1.0, and 2.0 equiv of 18-crown-6. The experimental results confirm that the reaction rate is dependent on the concentration of crown ether. Considering 2 h of reaction time, it is evident the highest conversion was obtained with the use of 2 equiv of the crown ether (46%), whereas the use of 1.0 equiv led to substantially less products (31%). In addition, the reaction goes to 100% completion in 22 h when using 2 equiv, whereas 1 equiv does not lead to total conversion even considering 48 h of reaction. Rather, the reaction slowed considerably, in line with the calculated free energy profile that points out the retarding effect of KBr being formed. As an approximated estimation of the Δ*G*^‡^ barrier, considering eq 6 and 2 h of reaction time with 2 equiv of 18C6, we can calculate that Δ*G*^‡^ = 27.0 kcal mol^–1^, in excellent agreement with our theoretically calculated value of 25.9 kcal mol^–1^ presented in Figure 2 for the S_N_2 process.

**Figure 5:**
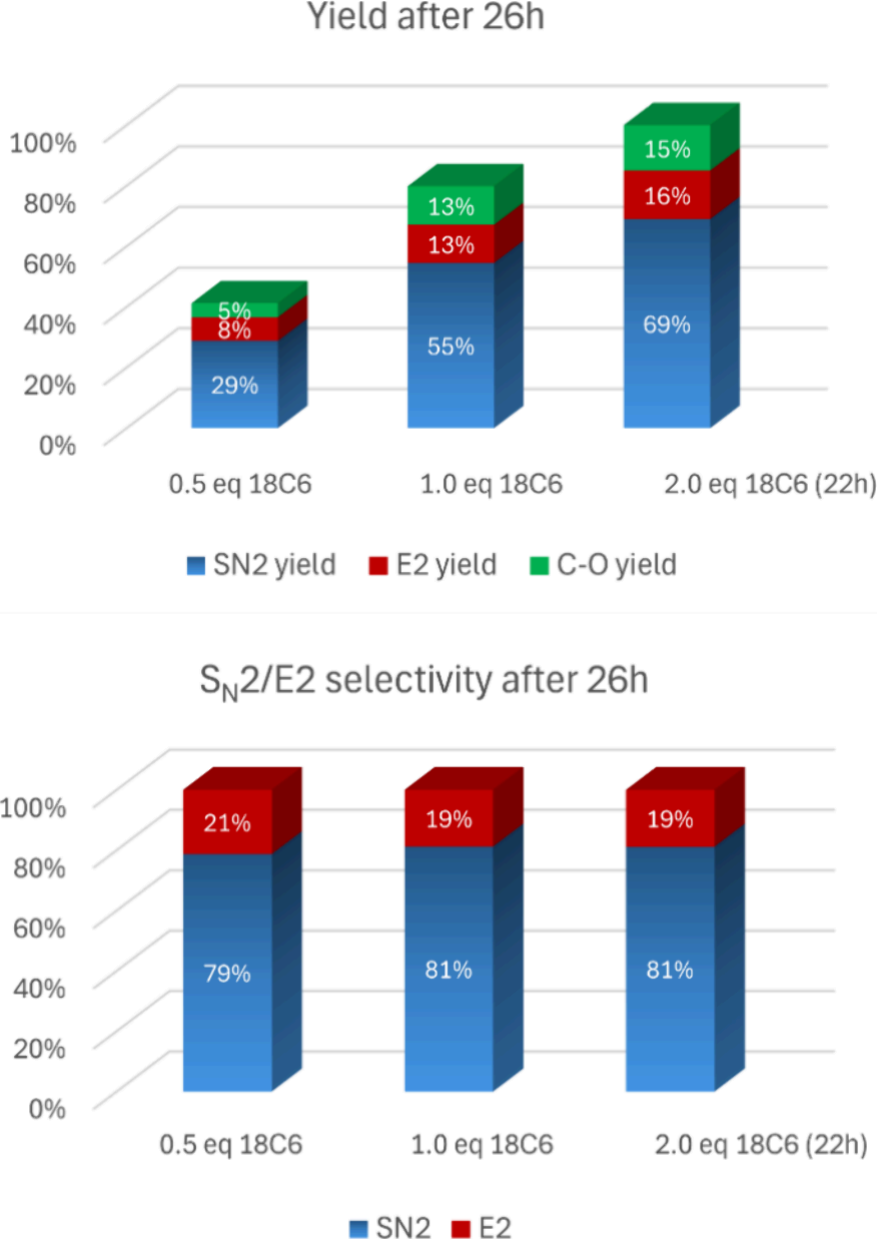
Effect of 18C6 concentration after 26 h in the yield and selectivity of the fluorination reaction of substrate **1**. Conditions: 1 mmol of substrate **1**, 2 mmol of KF, 4.0 mL of acetonitrile, 82 °C.

As for the selectivity (Figure 5), we can see the formation of an elimination product (**3**) as well as hydrolysis (**4**), indicating that water present in the medium participates in the reaction. These water molecules should form a complex of the kind KF-18C6-H2O similar to the alcohols. It is worth observing that 18C6 is hygroscopic and water is also present in the acetonitrile solvent. Consequently, both the hydrolysis and E2 reaction competition led to lower yields of fluorinated compounds, with less than 70% total selectivity for S_N_2. Another important point to observe is that water present in the medium can change the S_N_2/E2 selectivity and explain why the calculations have indicated 54:46 for the S_N_2/E2 product ratio, and the experimental results are 81:19 (22 h). Our initial view of using bulky alcohols with stronger hydrogen bonding donation ability as the fluorinated bulky alcohols is that these alcohols would replace acetonitrile and even water molecules in the solvation of fluoride ion, reducing both E2 and hydrolysis side reactions. Thus, no drying procedures were used in this study.

### 3.4 Effect of the Bulky Alcohols on the Kinetics

**Figure 6:**
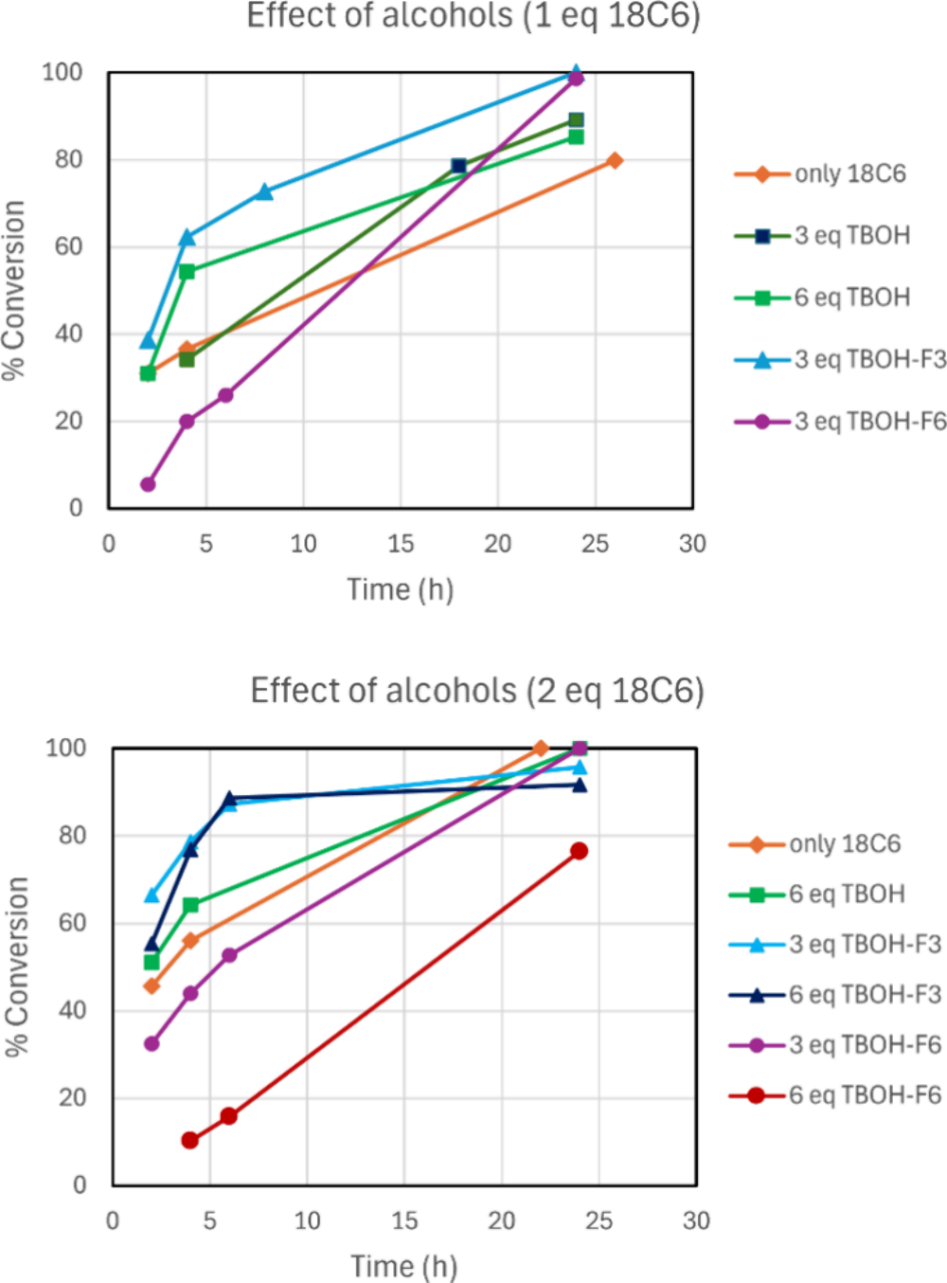
Kinetics of the conversion of substrate **1** on different 18-crown-6 and bulky alcohol combinations. Conditions: 1 mmol of substrate **1**, 2 mmol of KF, 4.0 mL of acetonitrile, 82 °C.

The effect of the addition of a stoichiometric amount of bulky alcohols on the kinetics of the reaction of KF with substrate **1** can be seen in Figure 6. Considering 4 h of reaction time and 1 equiv of 18C6, the alcohols TBOH (6 equiv) and TBOH-F3 (3 equiv) are able to accelerate the conversion better than the reference case using the crown ether only, with the latter being the most effective. In the case of TBOH-F6 (3 equiv), the reaction is slower. This observation agrees with the free energy profiles in Figures 2 and 3 because the barrier for the S_N_2 reaction involving TBOH-F3 is 2 kcal mol^–1^ smaller than that involving TBOH-F6 (KF-18C6-ROH reference point). It is worth observing that the S_N_2 reaction involving crown ether only has a slightly smaller Δ*G*^‡^ (by 0.8 kcal mol^–1^) than that involving TBOH-F3. Thus, the experimentally observed slower kinetics of using only 18C6 could be related to the uncertainty in our calculations, suggesting that the real barrier for the reaction involving TBOH-F3 is lower. A second possible explanation is the presence of water in the solution phase, which retards the reaction promoted by 18C6.

Another interesting point to observe when using 1 equiv of 18C6 combined with the bulky alcohols is that the reaction slows for TBOH and TBOH-F3. Nevertheless, the reaction goes to complete conversion at 24 h for TBOH-F6, suggesting that its kinetics is less affected by the advance of the reaction. This fact can be explained by the free energy profile of Figure 3, where the formation of the KF-18C6-ROH complex is so favorable that this alcohol can suppress the inhibition effect of the released KBr observed for the other alcohols. In the case of Figure 2, the free energy cost to release the KBr leads to the retardation of the reaction.

When the reaction is performed using 2 equiv of 18C6, the reaction rate is evidently increased for all of the combinations, and the use of TBOH-F3 (3 or 6 equiv) leads to almost 90% conversion at 6 h of reaction time. Nevertheless, after this time, the reaction rate slows down, and the reaction has 96% of conversion (3 equiv) at 24 h. In the case of TBOH-F6, we also tested the use of 3 and 6 equiv of this alcohol. Whereas the use of 3 equiv has resulted in reasonable kinetics, with full conversion at 24 h of reaction, the use of 6 equiv led to a considerable retarding of the reaction, with 76% conversion at 24 h of reaction. A possible explanation for this finding is the formation of an unreactive KF-18C6-ROH_2_ complex, which reduces the concentration of the active KF-18C6-ROH complex.

For both TBOH-F3 and TBOH-F6, an approximate estimation can be done for the experimental Δ*G*^‡^ using 2 equiv of 18C6 and 3 equiv of the fluorinated alcohols. Considering the free energy profiles and a short reaction time, the kinetics should follow the law:
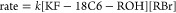
7a second-order kinetics. Considering 2 h of reaction time and the integrated kinetics law, we can calculate that Δ*G*^‡^ = 26.5 kcal mol^–1^ for TBOH-F3 and Δ*G*^‡^ = 27.3 kcal mol^–1^ for TBOH-F6 from the KF-18C6-ROH reference point. These estimated values are close to those presented in the theoretical free energy profiles of 26.7 and 28.7 kcal mol^–1^, respectively, for the S_N_2 reaction. This very good agreement between theory and experiment can be attributed to the most reliable theoretical calculations used for this kind of system in this work, such as more accurate functionals, solvent effect included in the optimizations, and more accurate treatment of low vibrational frequencies. In addition, the experimental kinetics was estimated at the beginning of the reaction.

### 3.5 Yield and Selectivity at a Short Reaction Time

**Figure 7:**
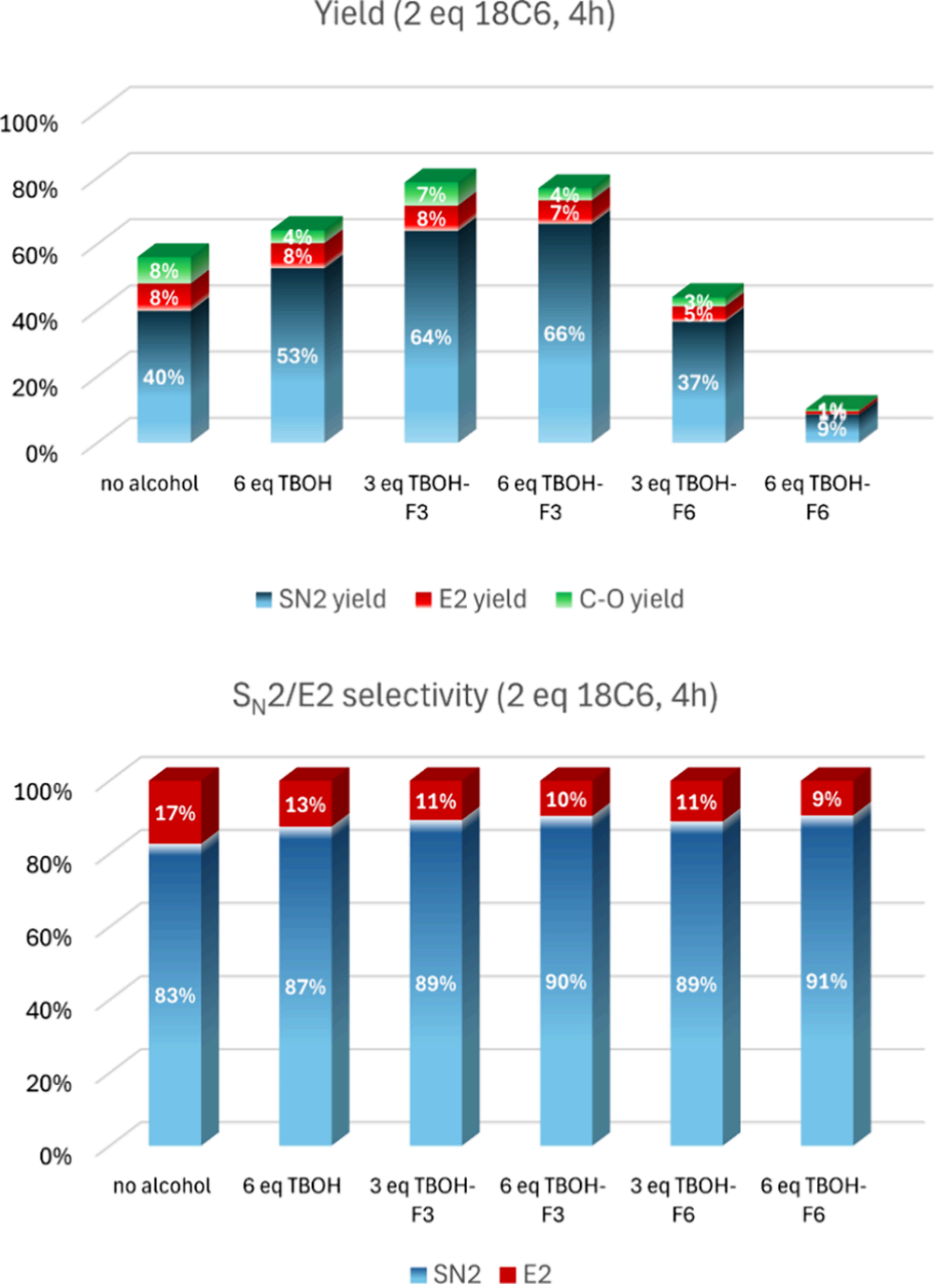
Yield and selectivity of the reaction of substrate **1** using different bulky alcohols and 4 h of reaction time. Conditions: 1 mmol of substrate **1**, 2 mmol of KF, 2 mmol of 18C6, 4.0 mL of acetonitrile, 82 °C.

The selectivity of the reaction is another important aspect to be considered. For better analyzing the effect of the alcohols and excluding side reactions in the latter reaction time, we chose 4 h of reaction time and used 2 equiv of 18C6. The results are presented in Figure 7. The use of 18C6 leads to only a 40% yield of S_N_2, 8% of E2, and 8% of hydrolysis (C–O yield). Considering only S_N_2/E2 selectivity, there is 83% of S_N_2. Because hydrolysis is observed, we wonder if this selectivity is partially affected by the water molecules, inducing more S_N_2. The use of 6 equiv of TBOH improves the S_N_2 yield to 53%, producing less hydrolysis (4%) and 87:13 for the S_N_2/E2 product ratio (87% selectivity). The TBOH-F3 alcohol has a more enhanced effect, leading to a 66% yield of S_N_2 (6 equiv of the alcohol) and 90% selectivity of S_N_2/E2. A small yield of 4% of hydrolysis is also observed. Considering the more fluorinated TBOH-F6 alcohol, the retarding effect on the kinetics is observed with a yield of 37% (3 equiv of the alcohol) and a high selectivity of 89% for S_N_2/E2. The conversion is very low for the use of 6 equiv of this alcohol.

Controlling the S_N_2/E2 regioselectivity is a very important aspect of this reaction from a practical point of view. When we observe the theoretical free energy profiles of Figures 2 and 3, the difference of 5 kcal mol^–1^ in the Δ*G*^‡^ in favor of S_N_2 over E2 in the transition states involving the coordinated alcohols indicates that only the S_N_2 product should be observed. The fact that close to 10% of E2 is experimentally observed indicates that this mechanism is being mediated by another pathway. In our view and based on the free energy profiles of Figures 2 and 3, the parallel E2 reaction occurs by the KF-18C6 complex, which has high S_N_2/E2 competition, and this species must remain present in a small concentration. In addition, its faster kinetics (Δ*G*^‡^ = 21.2 kcal mol^–1^ from soluble KF-18C6) could lead to a small formation of this side product, explaining the experimental findings. A similar behavior was proposed for fluorination involving TBAF complexes with *t*-butanol.^74,75^ For the present system, quantitative predictions of product ratio require a detailed microkinetic analysis.^76−78^

Beyond the reactions of fluorination via S_N_2 and elimination, the hydrolysis reaction leading to alcohol product **4** is an important side reaction. As discussed in a later section, the hydrolysis yield depends on the amount of water molecules in the solution phase, which is 0.008 mol/L for acetonitrile.^79^ Because the reaction was carried out in an open system, water present in the atmosphere can be absorbed by the acetonitrile solution, and a longer reaction time leads to more water being absorbed. However, the water molecule is a weak nucleophile, and its p*K*_a_ is very high, limiting the formation of much more reactive hydroxide ions. Thus, in principle, the hydrolysis product should be unfavorable. A provable mechanism for explaining the hydrolysis product could be the fluoride ion acting as a base in the transition state of nucleophilic water or even an equilibrium of deprotonation of water driven by the formation of the stable HF_2_^–^ ion.

Another side reaction leading to C–O bond formation is the etherification of the additive alcohol product **5**. The ether product was observed for the most fluorinated TBOH-F6 alcohol, whereas the hydrolysis product was observed for the less fluorinated alcohols. Indeed, more fluorinated alcohols are easier to deprotonate, in line with the lower p*K*_a_ value in Table 1. Although these alcohols have excellent hydrogen-bonding donating properties, fluorination yield becomes compromised for the hexafluorinated alcohols. This point is better discussed in Section 3.6.

### 3.6 Best Yield of S_N_2 for Each Alcohol

**Figure 8:**
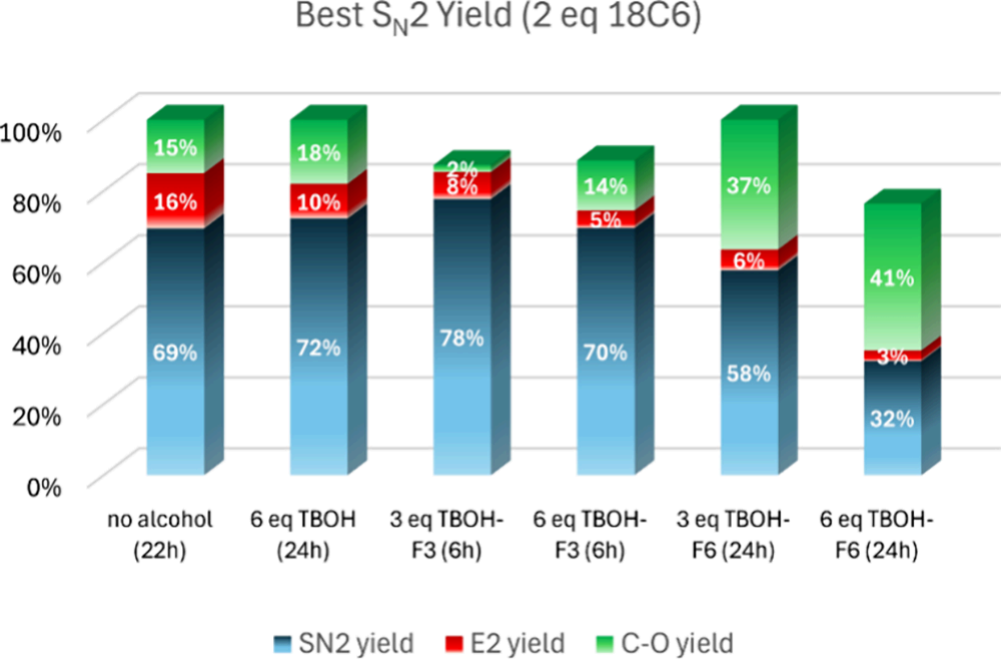
Yield of the reaction of substrate **1** using different bulky alcohols and considering the higher S_N_2 fluorination yield. Conditions: 1 mmol of substrate **1**, 2 mmol of KF, 2 mmol of 18C6, 4.0 mL of acetonitrile, 82 °C.

The best performance of the alcohols for yielding the fluorination product is presented in Figure 8. We can notice that TBOH-F3 (3 equiv) presents the best performance in yield and reaction time, leading to 78% S_N_2 yield in just 6 h of reaction time, with an S_N_2/E2 product ratio of 91:9. The use of 6 equiv of TBOH-F3 and 6 h of reaction time also leads to a good yield of 70%, with a very high S_N_2/E2 product ratio of 93:7. Thus, this latter combination could be especially useful in the cases of secondary alkyl bromides because a high suppression of side E2 process would be more challenging. For comparison, the crown ether only leads to a 69% yield and an S_N_2/E2 product ratio of 82:18 in 22 h. Although we have not investigated this in this study, a more controllable environment with lower water concentration could reduce the hydrolysis product.

In the case of TBOH-F6, the best S_N_2 yield (58%) was obtained with 3 equiv of the alcohol and 24 h of reaction time, leading to the complete conversion of the alkyl bromide substrate. The S_N_2/E2 selectivity is also high (91:9), and the major drawback in this case is the high C–O yield of 37%. The high yield of this side product is due to the formation of ether **5** from TBOH-F6, which was not observed for TBOH and TBOH-F3. Increasing the concentration of this alcohol using 6 equiv, both the reactivity and selectivity decreased. The total conversion becomes 76%, with a higher C–O yield (product **5**) of 41%.

### 3.7 Formation of Alcohols and Ether Products by Hydrolysis and Alcoholysis (C–O Product)

**Scheme 2:**
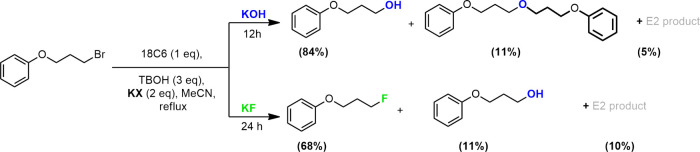
Control Experiments for the Generation of the C–O Products

Aimed at better understanding the formation of products **4** and **5**, we performed some control experiments in the presence of strong bases, such as KOH and *t*BuOK, under phase transfer catalysis (PTC) conditions. In the presence of KOH and TBOH as sources of base and bulky alcohol, respectively (control experiment 1, SI), we observed that the hydrolysis product was obtained mostly (88%), in addition to a small portion of a dimeric ether (11%), and no *t*-butyl ether was formed (Scheme 2). This finding indicates that nucleophilic *t*-butoxide is not generated. When we compare the reaction of KF with that of the alkyl bromide under the same conditions, only the alcohol appears as a minor product (Scheme 2). This product is formed due to the presence of water in the solution because we performed these reactions in the absence of an inert atmosphere and the solvent was not dried. These findings suggest that the alcohol product is not deprotonated by the weaker KF base or that the amount of dimeric ether is too low to be detected under KF as a base.

**Figure 9:**
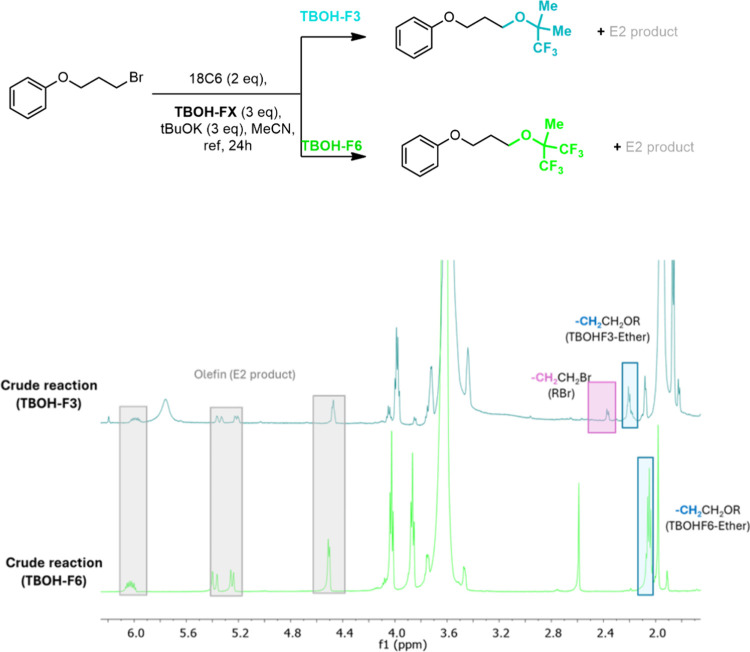
Control experiments for generation of the ethers of the fluorinated bulky alcohols showing the ^1^H NMR spectra of TBOH-F3 and TBOH-F6 under a strong basic medium and PTC conditions.

We tested the tBuOK strong base under the same conditions as Scheme 2, and we did not observe the formation of the ether of TBOH (Scheme S4 in the SI). For the case of the fluorinated bulky alcohols, we also evaluated their behavior under PTC conditions and a strong basic medium (Figure 9). We observed that these more acidic fluorinated alcohols can be deprotonated by tBuOK and react with the substrate via S_N_2, in line with Table 1. Considering reactivity, the bulky TBOH-F6 alcohol reacted completely by forming the respective TBOH-F6 ether and E2 product in a ratio of 3:1 in 24 h of reaction time. Under the same conditions, the TBOH-F3 alcohol formed the ether and E2 product in a ratio of 2:1 without total conversion.

**Figure 10:**
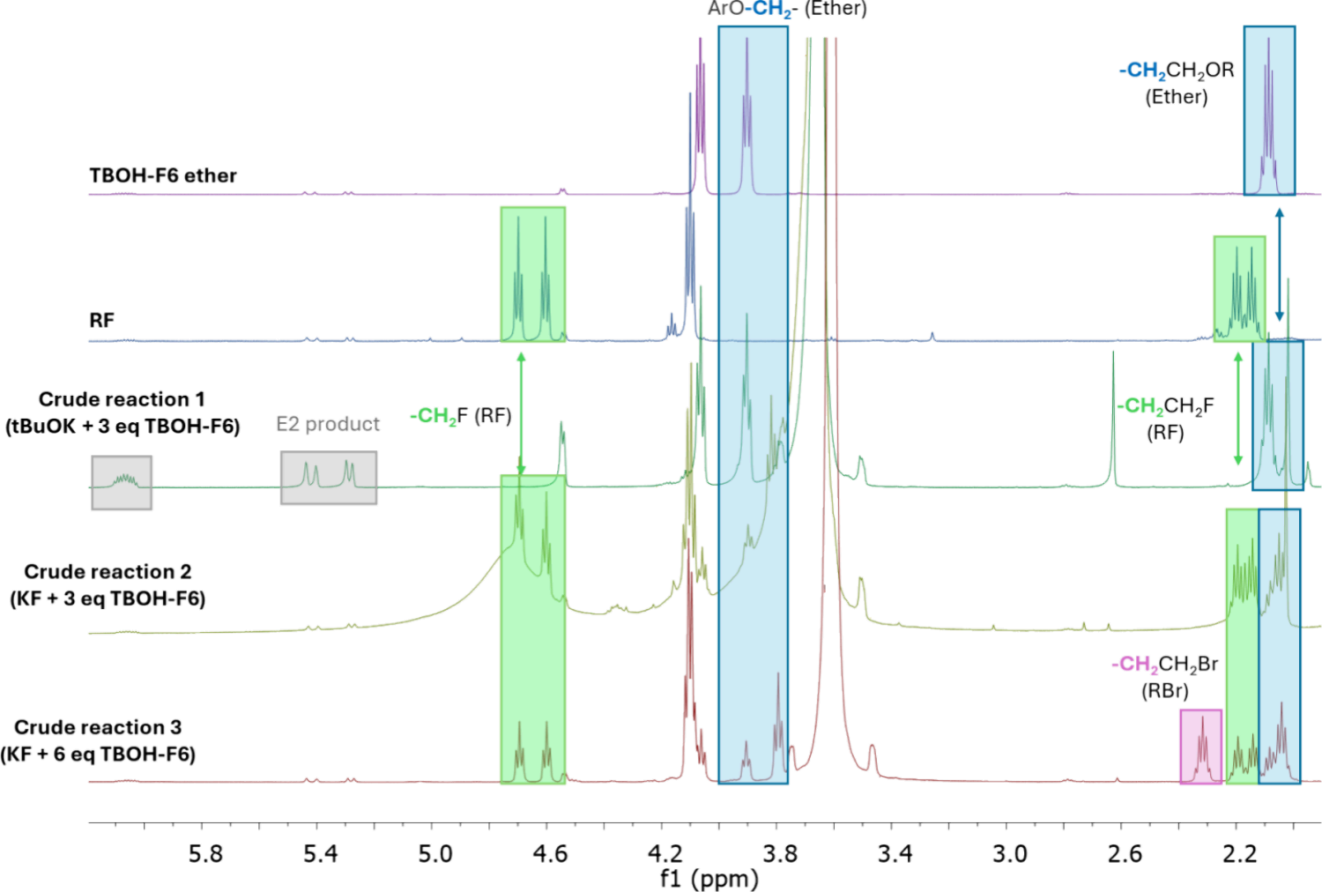
Experiments and the respective ^1^H NMR spectra supporting the formation of the TBOH-F6 ether as a side product of the fluorination with KF. In the experiments, 1 mmol of the alkyl bromide substrate **1**, 1 mmol of 18-crown-6, 4 mL of acetonitrile solvent, and 82 °C (reflux) were used.

The formation of TBOH-F6 ether (**5**) as a side product in the fluorination of the alkyl bromide substrate reacting with KF in the presence of 18-crown-6 and the TBOH-F6 alcohol is presented in Figure 10. We can see the signal of the ether in an aliquot of the crude reaction compared to the isolated ether and with its signal as a product of the reaction promoted by the tBuOK strong base. The formation of this kind of ether was not observed for TBOH and TBOH-F3, which can be justified by their substantially higher predicted p*K*_a_ values in acetonitrile solvent (Table 1). Among the F6 alcohols, TBOH-F6 has the highest p*K*_a_ of 26.2. Therefore, the other tested F6 alcohols and the BDMb-F12 alcohols, which have even lower values of p*K*_a_, could also present the same side ether product, compromising their efficiency for promoting the monofluorination reaction. We discuss these cases in a separate section.

### 3.8 Performance of Several F6 and F12 Alcohols

**Figure 11:**
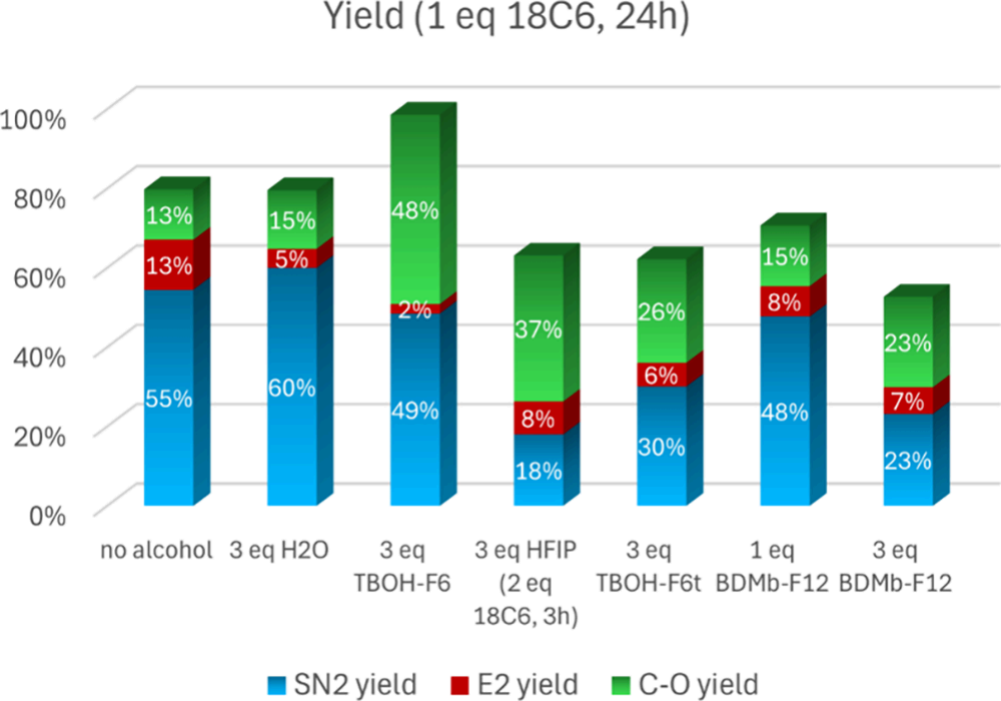
Yield of the reaction of substrate **1** using all of the F6 and F12 bulky alcohols. Conditions: 1 mmol of substrate **1**, 2 mmol of KF, 1 mmol of 18C6, 4.0 mL of acetonitrile, 82 °C.

The effect of highly fluorinated alcohols, which are more acidic, in combination with 1 equiv of crown ether is presented in Figure 11. We also included the effect of the addition of 3 equiv of water to the solution. As expected, more water leads to less E2 product and higher hydrolysis. The TBOH-F6 alcohol leads to a considerable conversion of 99%, with a substantial suppression of E2 product. However, the fluorination yield is only 49%, forming 48% of the related ether **5**. The widely used hexafluoroisopropanol (HFIP) alcohol led to an even worse result, with 18% of S_N_2 fluorination and 37% of ether **5** formation in 3 h of reaction and using 2 equiv of the crown ether. Thus, the steric effect of the methyl groups close to the hydroxyl group seems to be very important in decreasing the formation of the C–O product. The TBOH-F6t alcohol also has a performance worse than TBOH-F6, with a 30% yield of S_N_2 fluorination, 6% of E2, and 26% of ether formation. This lower performance can be attributed to its lower p*K*_a_, resulting in stronger hydrogen bonding and easier deprotonation. The analysis of the NMR spectra showing the formation of these ethers is presented in the SI.

**Figure 12:**
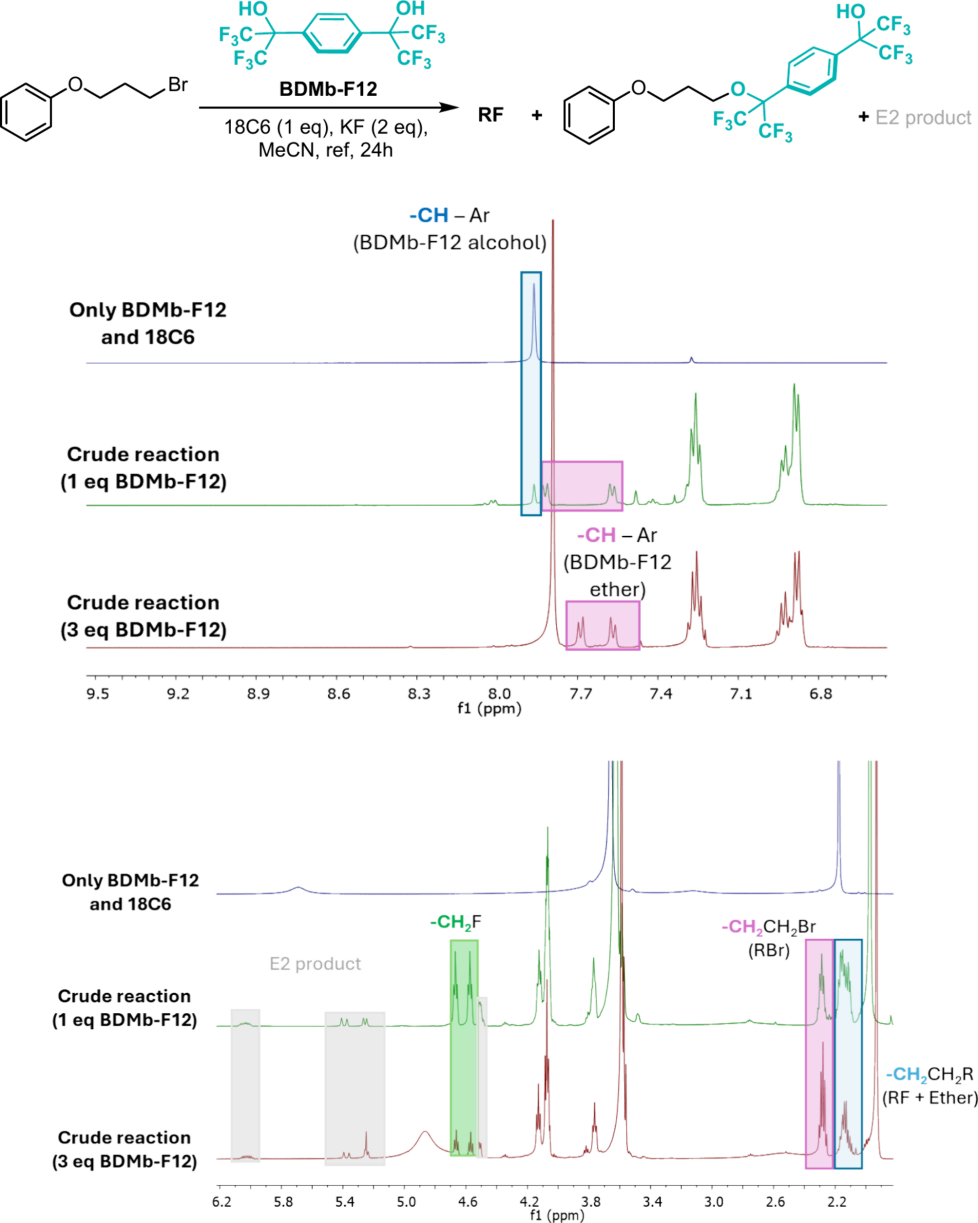
^1^H NMR spectra of fluorination reactions using BDMb-F12 (1 and 3 equiv) and 1 equiv of 18C6, 24 h.

The last tested alcohol was the fluorinated bulky diol BDMb-F12. Our initial idea was that this alcohol could work like the BMDb alcohol to stabilize the transition state.^16^ Unfortunately, its low p*K*_a_ calculated to be 23.5 resulted in the easy deprotonation of this alcohol, leading to the formation of the corresponding ether. Nevertheless, even with this higher acidity, the use of 1 equiv of this ether led to 48% S_N_2 and only 15% of ether. Increasing the concentration of this alcohol to 3 equiv resulted in a slower reaction rate and a higher proportion of the ether product. In this case, ether formation is evidenced by the desymmetrization of aromatic hydrogen atoms (Figure 12). When we compare it to TBOH-F6, the same is observed by the decreasing of both reactivity and selectivity when increasing the alcohol concentration.

### 3.9 Test of TBOH-F3 for a Secondary Alkyl Bromide Substrate

**Scheme 3:**
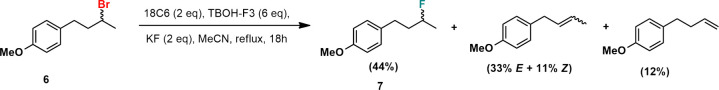
Fluorination of a Secondary Alkyl Bromide with KF under the Effect of TBOH-F3 and 18-Crown-6 Combination

Our final investigation was to evaluate the effect of the best-performing TBOH-F3 alcohol on the more challenging secondary alkyl bromide substrate. The results are presented in Scheme 3 and in the SI. Considering that a higher suppression of the E2 product is desirable, we have used 6 equiv of the alcohol. When we compared the behavior of the primary alkyl bromide **1** (Scheme 1) and of the secondary alkyl bromide **6** (Scheme 3) substrates under the same conditions, we observed that after 2 h of reaction, the reactivity of both was similar, with the total conversion of 55 and 42% for the primary and secondary substrates, respectively (see the SI). After 18 h, we observed the total conversion of the secondary alkyl bromide while that of the primary bromide was 92% in 24 h. These results indicate that the combination of 18C6 and TBOH-F3 was efficient in promoting the reactivity of the secondary substrates. However, as expected, the S_N_2/E2 selectivity for the secondary substrate **6** at 18 h of reaction was lower than that observed for the primary substrate **1**, with 44% yield of fluorination and 56% yield of E2 against 75% yield of fluorination and only trace of the E2 product for the primary substrate. However, no hydrolysis product was observed for the secondary substrate.

An additional comparison can be made with other monofluorination procedures. For example, Trojan and co-workers^12^ have recently tested several difluorosilicates and reported that tetrabutylammonium difluorotriphenylsilicate (TBAT) was the most effective to fluorinate 2-bromooctane. Performing the reaction for 24 h at 85 °C, they obtained 92% conversion and an S_N_2/E2 product ratio of 39:61, resulting in a 36% fluorination yield. In another study, by modifying TBAT with the addition of an electron-donating group to the aromatic ring of this reactant, they obtained an up to 49% fluorination yield.^80^ A study using TBAF combined with diverse bulky alcohols has not resulted in S_N_2/E2 selectivity higher than 81:19 in a primary substrate, suggesting that this combination should not be effective for a secondary substrate.^75^ The use of diarylureas rather than bulky alcohols combined with TBAF has resulted in lower reactivity but improved selectivity up to 90:10 product ratio for S_N_2/E2 involving a primary substrate.^81^ Kim and co-workers have also reported the use of KF with 2 equiv of 18-crown-6 in *t*-amyl alcohol solvent for a primary alkyl bromide substrate, obtaining an S_N_2 yield of 93% and E2 yield of 6% (isolated yields) in 4 h of reaction time and higher temperature of 100 °C. No secondary alkyl bromide substrate was reported.^20^

## 4 Conclusions

The addition of a stoichiometric amount of hydrogen bonding donor bulky alcohols to an acetonitrile solution leads to a solvent system with an improved ability to dissolve KF salt by the use of 18-crown-6. In addition, this solvent system can accelerate the reaction rate and increase the S_N_2/E2 selectivity, enhancing the fluorination yield. Among the several tested fluorinated bulky alcohols, the use of TBOH-F3 (3 or 6 equiv) was the most effective, leading to improved kinetics and selectivity. A hydrolysis product was also observed due to the presence of water, which could be controlled by performing the reaction under more anhydrous conditions. Higher fluorinated alcohols such as TBOH-F6, HFIP, TBOH-F6t, and BDMb-F12 are considerably more acidic, resulting in the deprotonation of these alcohols and generation of the respective alkoxides. These alkoxides can react with the substrate, producing the respective ethers as a side product. Although less effective for monofluorination, these more fluorinated alcohols such as HFIP can be useful to introduce highly fluorinated fragments to an organic substrate.

The extensive experimental investigation in this work was assisted by a reliable computational investigation, which provides an important conceptual framework and insightful mechanistic pictures for the molecular-level processes. Our theoretical calculations are in very good agreement with experimentally estimated kinetics data and explain the improved selectivity induced by TBOH-F3. The present study reports an improved monofluorination method using readily available reactants, leading to effective fluorination with KF salt, even for a secondary alkyl bromide substrate under mild conditions.

## Data Availability

The data underlying this study are available in the published article and its Supporting Information.

## Supporting Information Available

The coordinates of the optimized structures, tables of the calculations, experimental procedure, NMR spectra, and HRMS spectra (PDF)
^1^H NMR signals and yields (Table S1) (XLSX)
^1^H NMR signals and yields (Table S2) (XLSX)
XYZ-F3 (ZIP)
XYZ-F6 (ZIP)
