# Supplementary material for: Effects of Hydrogen Bonding Solvation by Diverse Fluorinated Bulky Alcohols on the Reaction Rate and Selectivity in Crown Ether Mediated Nucleophilic Fluorination in an Aprotic Solvent
Source: ACS Org Inorg Au. 2024 Nov 28;5(1):69–83. doi: 10.1021/acsorginorgau.4c00081 (PMC11803469; doi:10.1021/acsorginorgau.4c00081)
Supplement: Supplementary file 1 — gg4c00081_si_001.pdf [file gg4c00081_si_001.pdf]

Supporting Information

Effects of Hydrogen Bonding Solvation by Diverse Fluorinated Bulky Alcohols on the Reaction Rate and Selectivity in Crown Ether Mediated Nucleophilic Fluorination in Aprotic Solvent

Eloah P. Ávila[a,b], Mauro V. de Almeida[a], Marcelo S. Valle[b]
and Josefredo R. Pliego.[b]*

a - Chemistry Department, Federal University of Juiz de Fora, Cidade Universitaria, São Pedro, Juiz de Fora, Minas Gerais, 36036-900, Brazil.
b - Departamento de Ciências Naturais, Universidade Federal de São João del-Rei, 36301-160, São João del-Rei, MG, Brazil.

* pliego@ufsj.edu.br

1. General Information of fluorination reactions 3

1.1. Experimental procedures and characterization data for the primary alkyl bromide 3

1.2. Experimental procedures and characterization data of the secondary alkyl bromide 3

1.2.1. Experimental procedures and characterization data of the secondary alkyl bromide preparation 3

1.2.2. Experimental procedures and characterization data of the fluorination reaction for the secondary alkyl bromide 4

1.3. NMR and HRMS spectra of optimization reaction conditions 6

1.3.1. ^{1}H NMR spectra of the crude reactions of Table S1 6

1.3.2. ^{1}H NMR spectra of the crude reactions of Table S2 32

1.3.3. ^{1}H NMR spectra of the crude reaction of the secondary substrate.......35

1.3.4. HRMS spectra of the fluorination reaction mixtures..............................36

1.4. NMR and HRMS spectra of the isolated compounds...................................38

2. Control experiments: background reactions of C-O bond formation under basic media - hydrolysis and etherification reactions...47

2.1. Experimental procedures 47

2.2. Characterization data of isolated alcohol, alkyl and aryl ether derivatives 48

2.3. NMR and HRMS spectra 50

3. Behavior of alkyl/aryl alcohols towards the selectivity: basicity x nucleophilicity of fluorine anion 65

3.3.1. Hydrolysis reaction 65

3.3.2. HFIP 67

3.3.3. TBOH-F6...68

3.3.5. TBOHt-F6-ether reaction formation...71

3.3.6. BDMb-F12 alcohol 73

4. Table of Theoretical Calculations...74

5. References..75

1. General Information of fluorination reactions

1.1. Experimental procedures and characterization data for the primary alkyl bromide

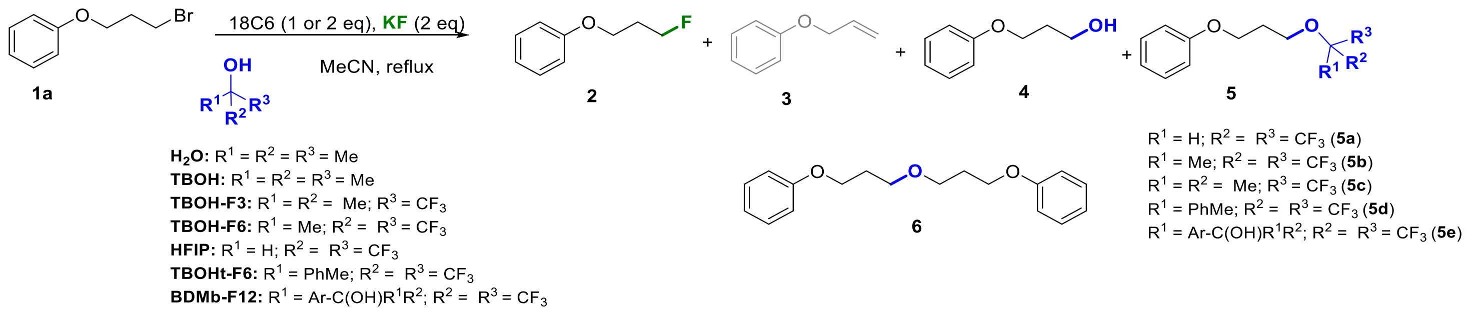

Scheme S1. General procedure of fluorination reactions promoted by different alcohols

In a round-bottomed flask, coupled to a condenser, the primary alkyl bromide (0.2591 g, 1 mmol), 18C6 (0.5, 1.0 or 2.0 mmol), KF (0.1160 g, 2 mmol) and bulky alcohols (3 or 6 mmol) were added to 4 mL of acetonitrile. The reaction was stirred under reflux and some aliquots of 0.5 mL were taken, in different times, then the solvent was removed under reduced pressure. The crude reactions were submitted to ^{1}H NMR measurements to obtain the conversion rates (Tables S1 and S2). The yields of fluorinated compound (**2**) were measured only by ^{1}H NMR analyses. The isolated yields were not determined due to difficult complete separation, which would lead to reduced yield values. The pure spectra of **2** and **5e** were obtained after column chromatography of the crude reaction of Entry 2 (Table S2), solvent hexane:AcOEt from 99:1 to 95:5 [1].

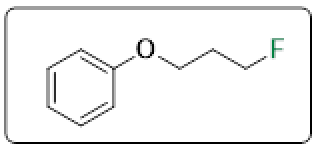

(3-fluoropropoxy)benzene (2): The product **2** was obtained as a colorless oil. ^{1}H NMR (500 MHz, $CDCl_3$) δ 7.28 (t, J = 7.8 Hz, 2H), 6.95 (t, J = 7.8 Hz, 1H), 6.94 (d, J = 7.8 Hz, 2H), 4.65 (dt, ^{2}J = 47.1, 5.8 Hz, 2H), 4.10 (t, J = 6.1 Hz, 2H), 2.17 (dqui, ^{3}J = 26.0, 5.9 Hz, 2H). $^{13}C\{1H\}$ NMR (125 MHz, $CDCl_3$) δ 158.8, 129.5, 120.9, 114.5, 80.8 (d, ^{1}J = 164.5 Hz, -**C**H_2F), 63.4 (d, ^{3}J = 5.2 Hz, PhO-**C**$H_2CH_2CH_2$F), 30.5 (d, ^{2}J = 20.0 Hz, -**C**H_2CH_2F).

1.2. Experimental procedures and characterization data of the secondary alkyl bromide

1.2.1. Experimental procedures and characterization data of the secondary alkyl bromide preparation

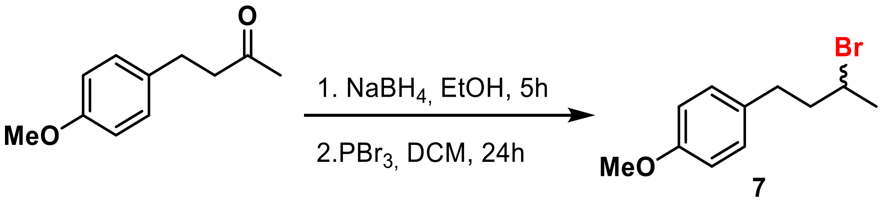

Scheme S2. Preparation of secondary alkyl bromide by reduction and bromination steps

To a suspension of $NaBH_4$ (1.6 g, 42 mmol) in EtOH (50 mL), at 0°C, an ethanolic solution of the starting ketone (5g, 28 mmol) was added dropwise. The reaction mixture was stirred at room temperature until the reaction was complete. The solvent was removed under reduced pressure, and

the reaction mixture was extracted with a saturated NaOH solution and purified by chromatographic column, providing the secondary alcohol intermediate (1.8 g, 36 % yield). [2]

Then, a solution of PBr_3 (4.05 g, 15 mmol) in 15 mL of DCM was dropwise added to a solution of alcohol (1.8 g, 10 mmol) in dichloromethane (15 mL) at 0°C. Then, the reaction mixture was stirred for 24h and the solvent was removed under reduced pressure. The crude reaction was purified with a gradient chromatographic column, providing the secondary bromide.[2]

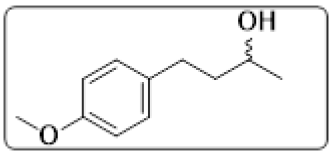

4-(4-methoxyphenyl)butan-2-ol (Intermediate alcohol): The secondary alcohol was obtained as a colorless oil (1.8 g, 36% yield). ^{1}H NMR (500 MHz, $CDCl_3$) δ 7.15-7.13 (m, 2H), 6.87-6.85 (m, 2H), 3.84 (qui, J = 6.2 Hz, 1H), 3.88 – 3.82 (m, 1H), 2.72 (ddd, J = 15.3, 9.3, 6.2 Hz, 1H), 2.68 – 2.60 (m, 1H), 1.83 – 1.70 (m, 2H), 1.25 (d, J = 6.2 Hz, 3H). ^{13}C{1H} NMR (125 MHz, $CDCl_3$) δ 157.8, 134.1, 129.3, 113.9, 67.5, 55.3, 41.1, 31.2, 23.6.

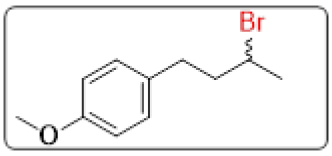

1-(3-bromobutyl)-4-methoxybenzene (7):

The secondary alkyl bromide was obtained as a colorless oil (0.200 g, 10% yield).^{1}H NMR (500 MHz, $CDCl_3$) δ 7.04-7.02 (m, 2H), 6.75-6.74 (m, 2H), 4.02 – 3.93 (m, 1H), 3.69 (s, 3H), 2.74-2.68 (m, 1H), 2.65 – 2.55 (m, 1H), 2.05 – 1.97 (m, 1H), 1.96 – 1.85 (m, 1H), 1.63 (d, J = 6.7 Hz, 3H). ^{13}C{1H} NMR (125 MHz, $CDCl_3$) δ 158.0, 133.0, 129.5, 113.9, 55.3, 51.0, 43.0, 33.1, 26.6.

1.2.2. Experimental procedures and characterization data of the fluorination reaction for the secondary alkyl bromide

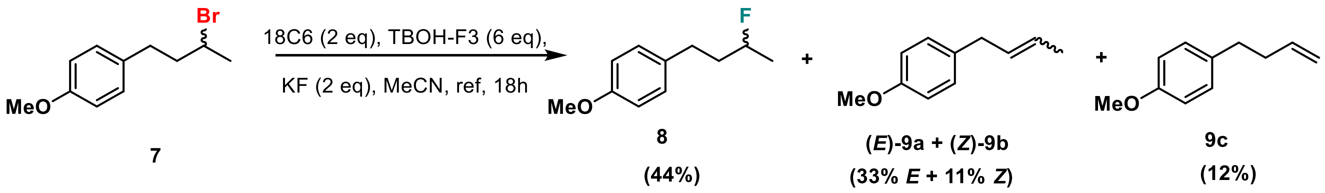

Scheme S3. Fluorination reaction of secondary alkyl bromide promoted by the best optimized condition

In a round-bottomed flask, coupled to a condenser, the secondary bromine (0.2420 g, 1 mmol), 18C6 (0.5280 g, 2 mmol) and KF (0.116, 2 mmol), and TBOH-F3 (0.7680 g, 0.61 mL, 6 mmol) were added in 4 mL of acetonitrile. The reaction was stirred under reflux and some aliquots of 0.5 mL were taken, at different times, and then the solvent was removed under reduced pressure. The yields were measured only by ^{1}H NMR analyses. The isolated yields were not determined due to difficult complete separation, which would lead to reduced yield values. The pure spectra of **8** and **9a** were obtained after column chromatography of the crude reaction, solvent hexane:AcOEt from 99:1 to 95:5.

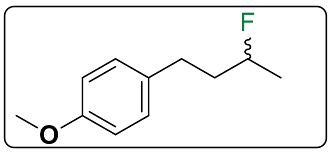

1-(3-fluorobutyl)-4-methoxybenzene (8): The product **8** was obtained as a colorless oil. ^{1}H NMR (500 MHz, $CDCl_3$) δ 7.13-7.11 (m, 2H), 6.85-6.83 (m, 2H), 4.74 – 4.57 (m, 1H), 3.79 (s, 3H), 2.80 – 2.70 (m, 1H), 2.70 – 2.59 (m, 1H), 2.02 – 1.90 (m, 1H), 1.87 – 1.71 (m, 1H), 1.34 (dd, *J* = 23.9, 6.2 Hz, 3H). ^{13}C{1H} NMR (125 MHz, $CDCl_3$) δ 157.9, 133.6, 129.3, 113.9, 90.0 (d, ^1J = 164.8 Hz), 55.3, 38.9 (d, ^2J = 20.7 Hz), 30.4 (d, ^3J = 4.9 Hz), 21.0 (d, ^2J = 22.7 Hz). HRMS (ESI) *m/z*: $[M]^+$ Calcd for $C_{11}H_{14}FO^+$ 181.1023; Found 181.1026; $[M+KF_2]^-$ Calcd for $C_{11}H_{15}F_3OK^-$ 259.0718; Found 259.0784.

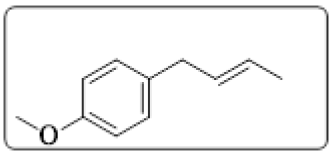

(*E*)-1-(but-2-en-1-yl)-4-methoxybenzene (9a): The product **9a** was obtained as a colorless oil. ^{1}H NMR (500 MHz, $CDCl_3$) δ 7.13-7.11 (m, 2H), 6.87-6.85 (m, 2H), 5.60 (dt, *J* = 12.8, 6.0 Hz, 1H), 5.51 (dt, *J* = 12.8, 6.0 Hz, 1H), 3.81 (s, 3H), 3.28 (d, *J* = 6.0 Hz, 2H), 1.71 (d, *J* = 6.0 Hz, 3H). ^{13}C{1H} NMR (125 MHz, $CDCl_3$) δ 158.0, 133.2, 130.5, 129.4, 126.0, 113.8, 55.3, 38.1, 29.7. HRMS (ESI) *m/z*: $[M]^+$ Calcd for $C_{11}H_{13}O^+$ 161.0961; Found 161.0969.

1.3. NMR and HRMS spectra of optimization reaction conditions

1.3.1. ^{1}H NMR spectra of the crude reactions of Table S1

Entry 1

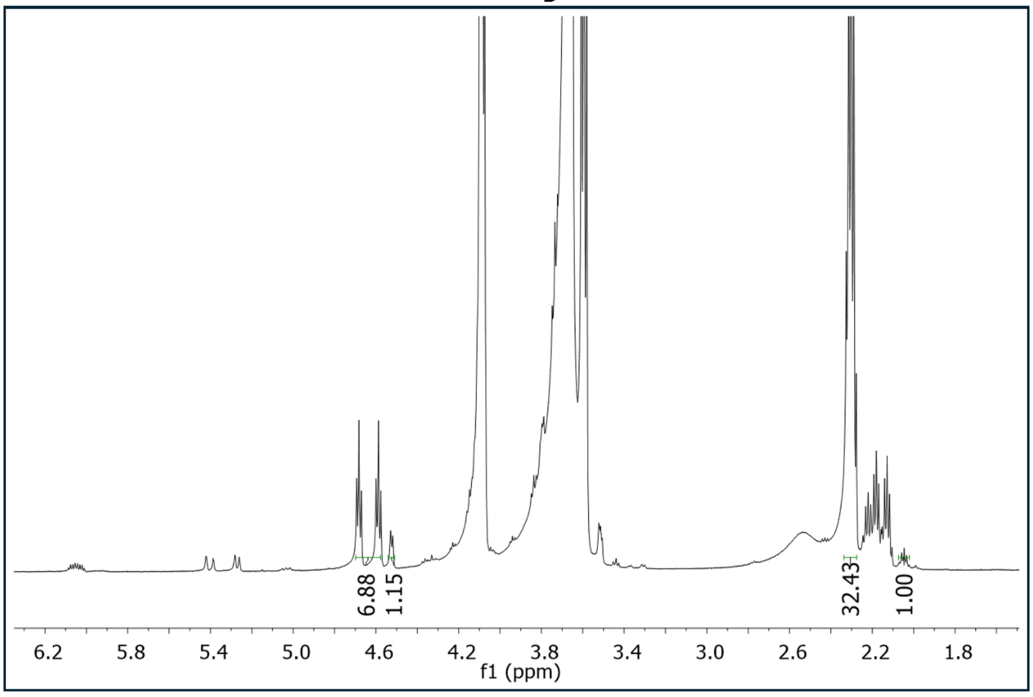

18C6 (50 mol %)
No alcohol, MeCN, reflux, **2h**

Entry 2

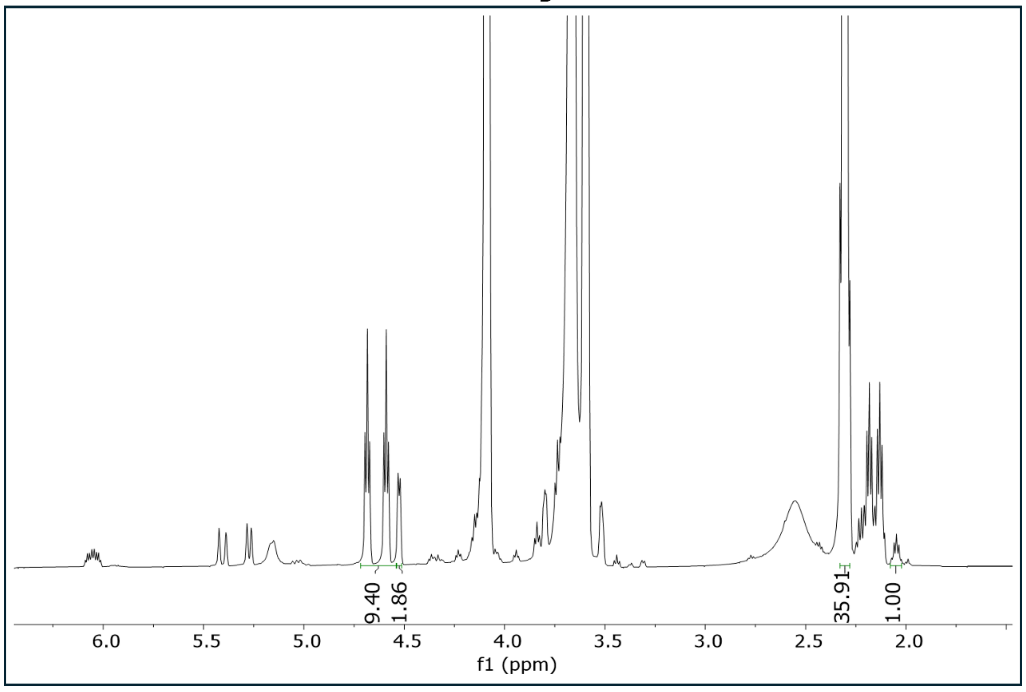

18C6 (50 mol %)
No alcohol, MeCN, reflux, **4h**

Entry 3

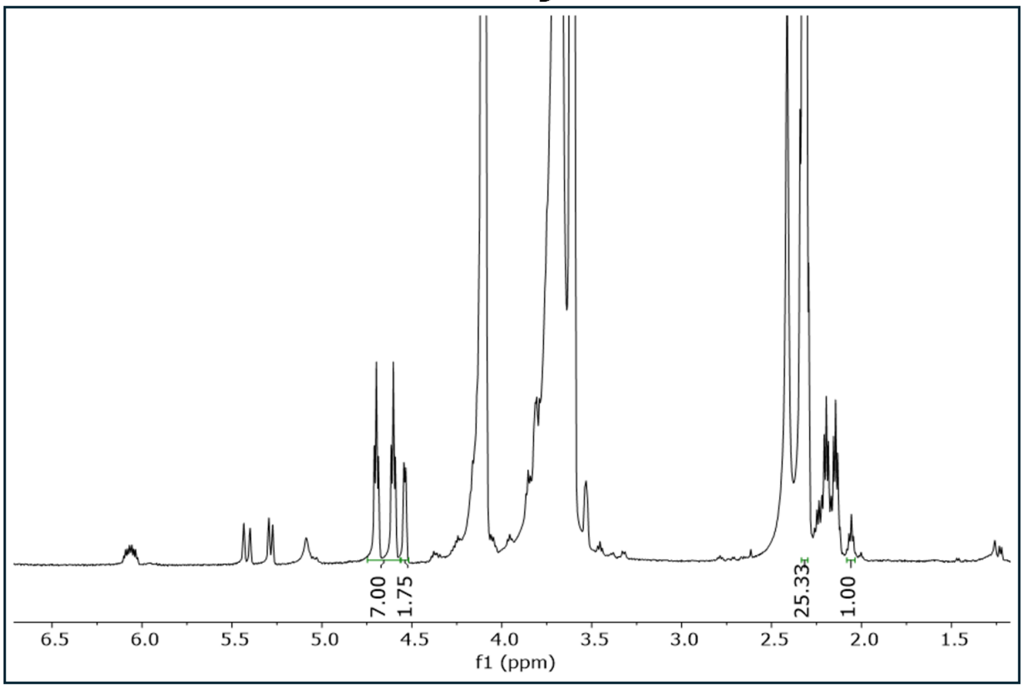

18C6 (50 mol %)
No alcohol, MeCN, reflux, **6h**

Entry 4

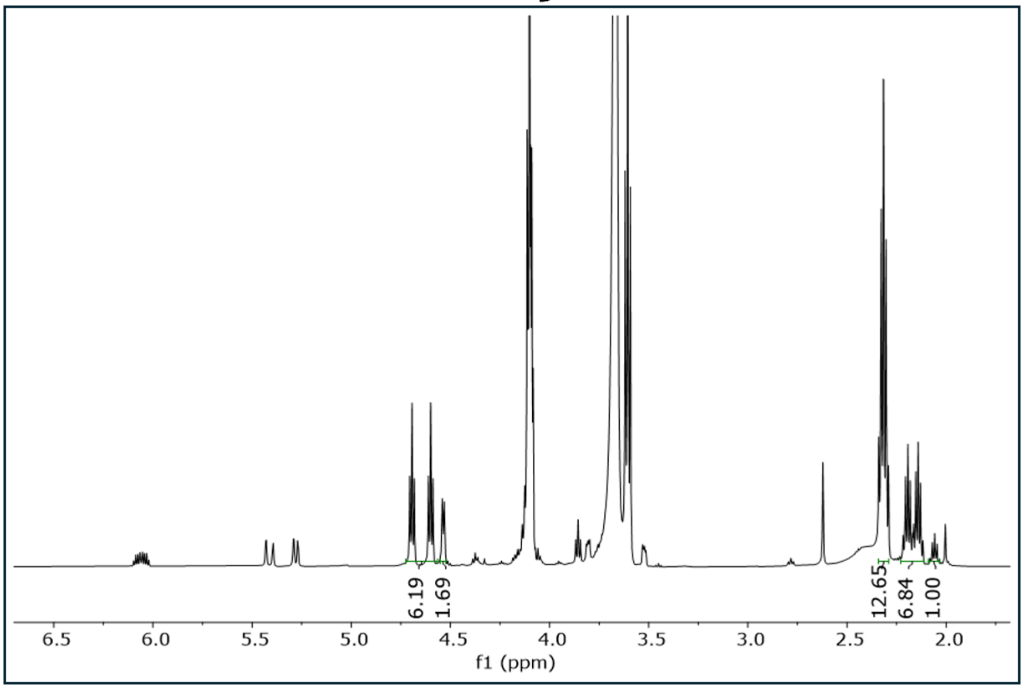

18C6 (50 mol %)
No alcohol, MeCN, reflux, **26h**

Entry 5

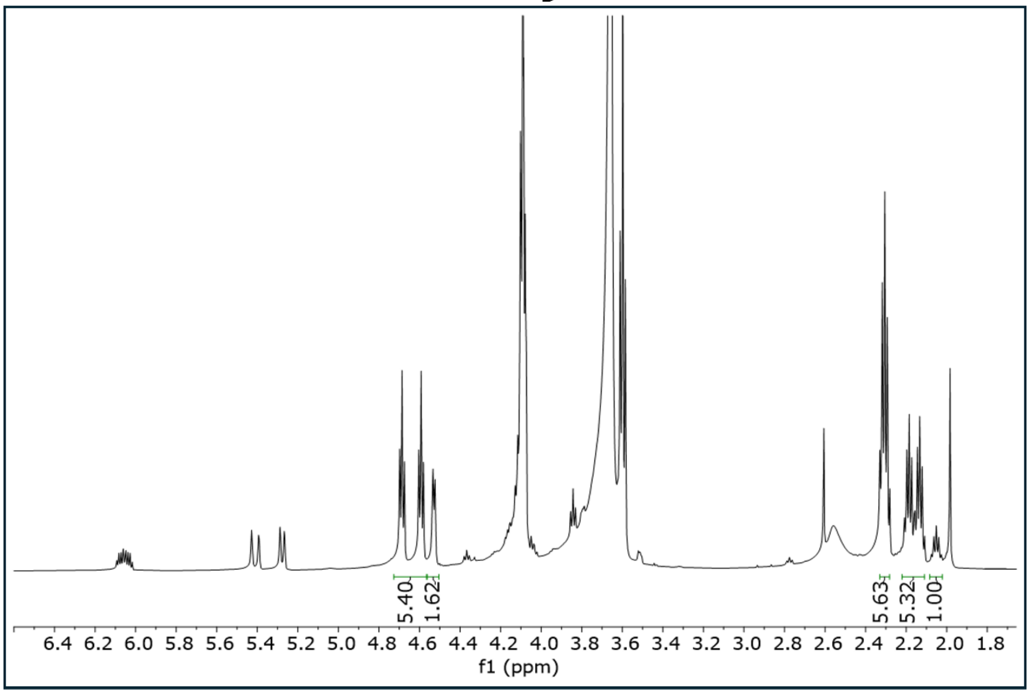

18C6 (50 mol %)

No alcohol, MeCN, reflux, **48h**

Figure S1. ^{1}H ^{1}H NMR ($CDCl_3$, 500 MHz) spectra of fluorination reaction 1 (1 mmol of RBr, 0.5 mmol of 18C6 and 2 mmol of KF) in 2, 4, 6, 26 and 48 h, Entries 1-5.

Entry 6

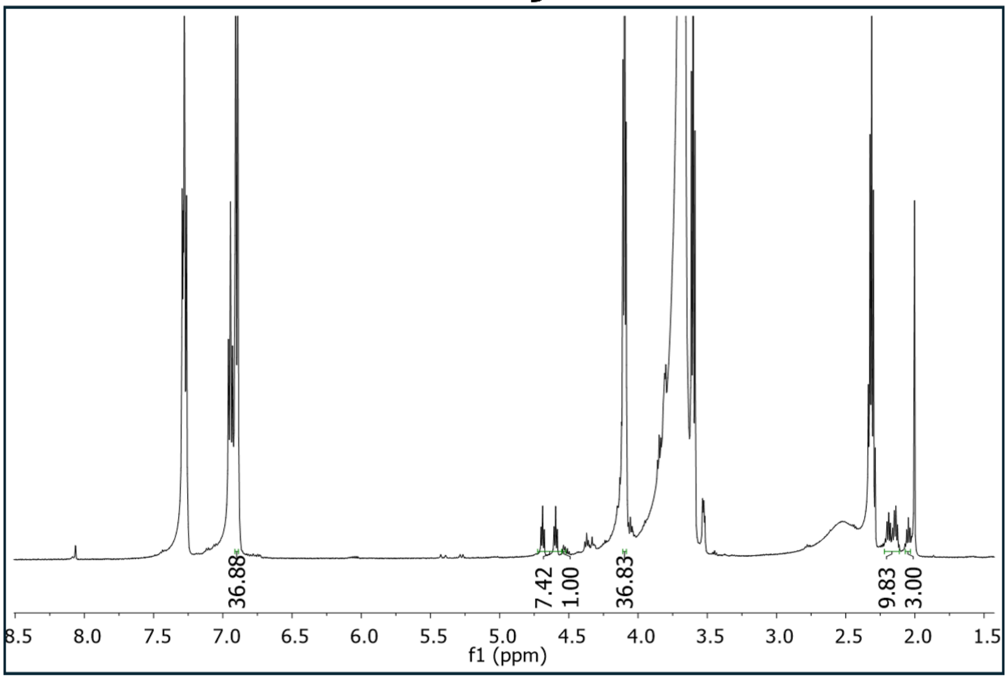

18C6 (1 mmol)

No alcohol, MeCN, reflux, **2h**

Entry 7

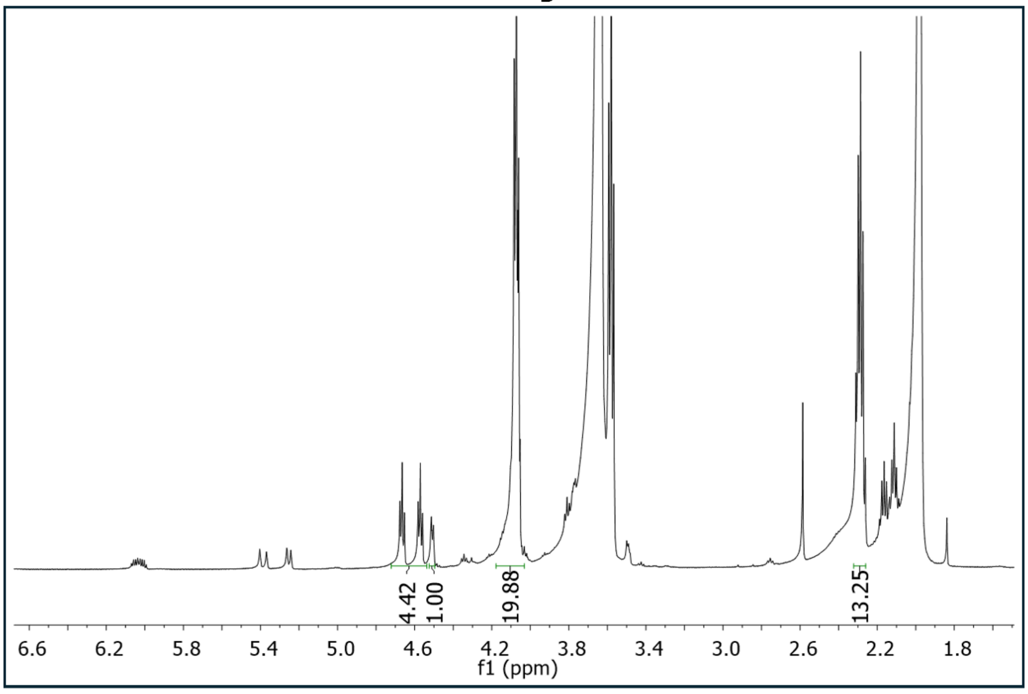

18C6 (1 mmol)

No alcohol, MeCN, reflux, **4h**

Entry 8

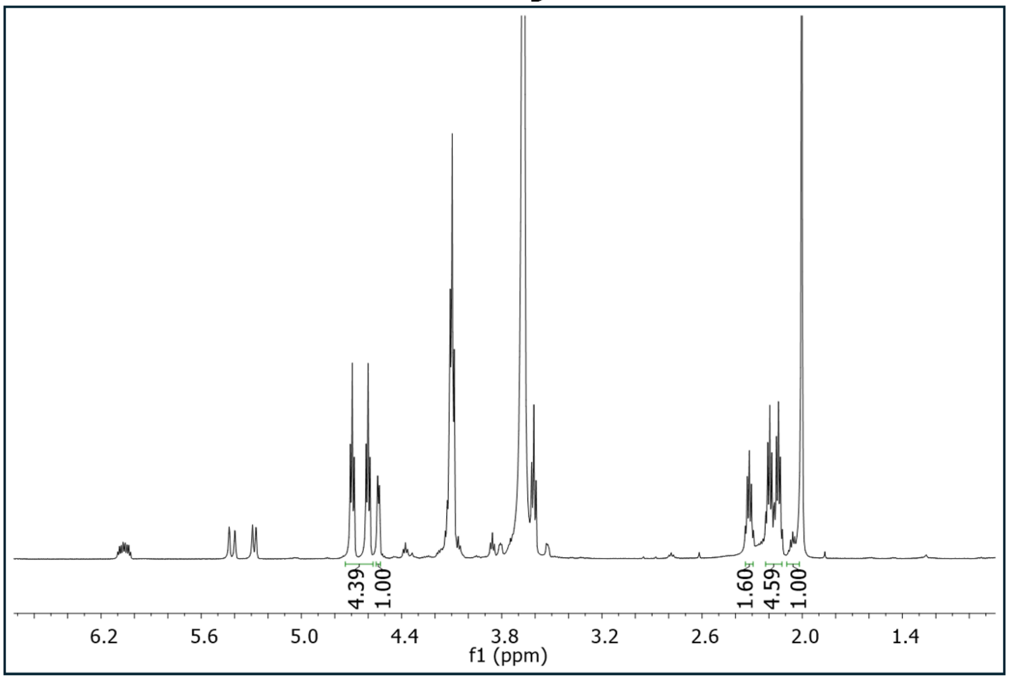

18C6 (1 mmol)
No alcohol, MeCN, reflux, **26h**

Entry 9

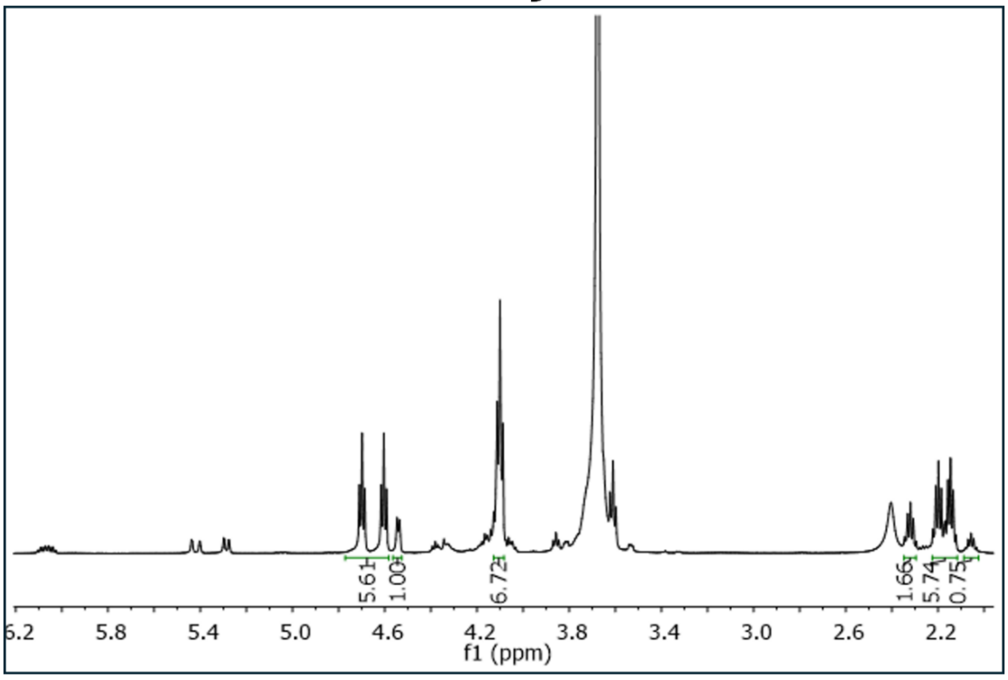

18C6 (1 mmol)
No alcohol, MeCN, reflux, **48h**

Figure S2. ^{1}H NMR ($CDCl_3$, 500 MHz) spectra of fluorination reaction 2 (1 mmol of RBr, 1 mmol of 18C6 and 2 mmol of KF) in 2, 4, 26 and 48h, Entries 6-9.

Entry 10

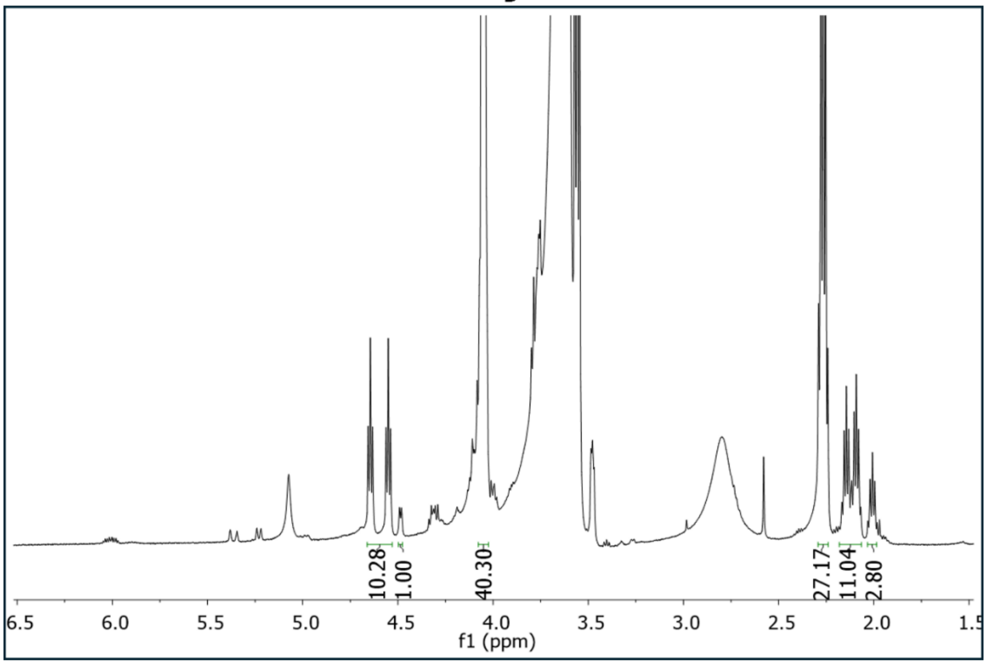

18C6 (1 mmol)
tBuOH (3 mmol), MeCN, reflux, **4h**

Entry 11

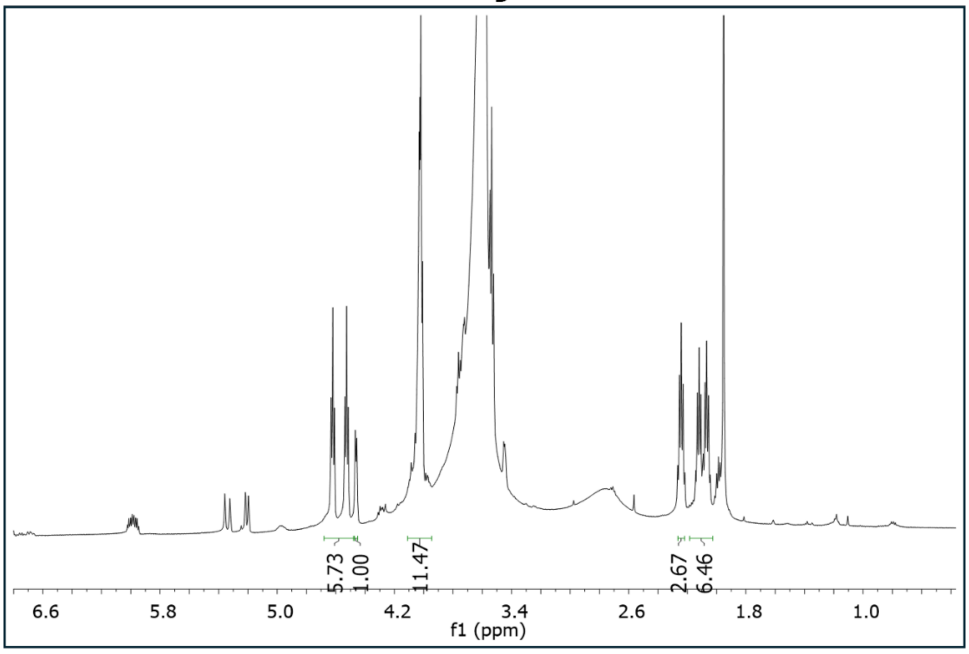

18C6 (1 mmol)
tBuOH (3 mmol), MeCN, reflux, **18h**

Entry 12

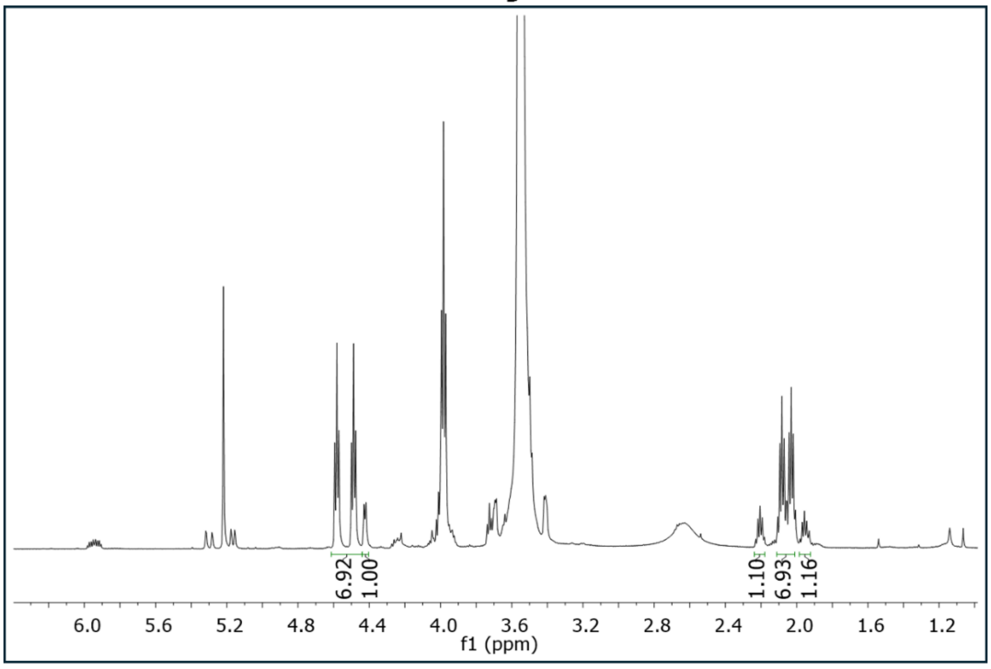

18C6 (1 mmol)

tBuOH (3 mmol), MeCN, reflux, **24h**

Figure S3. ^{1}H NMR ($CDCl_3$, 500 MHz) spectra of fluorination reaction 3 (1 mmol of RBr, 1 mmol of 18C6, 3 mmol of TBOH and 2 mmol of KF) in 4, 18 and 24 h, Entries 10-12

Entry 13

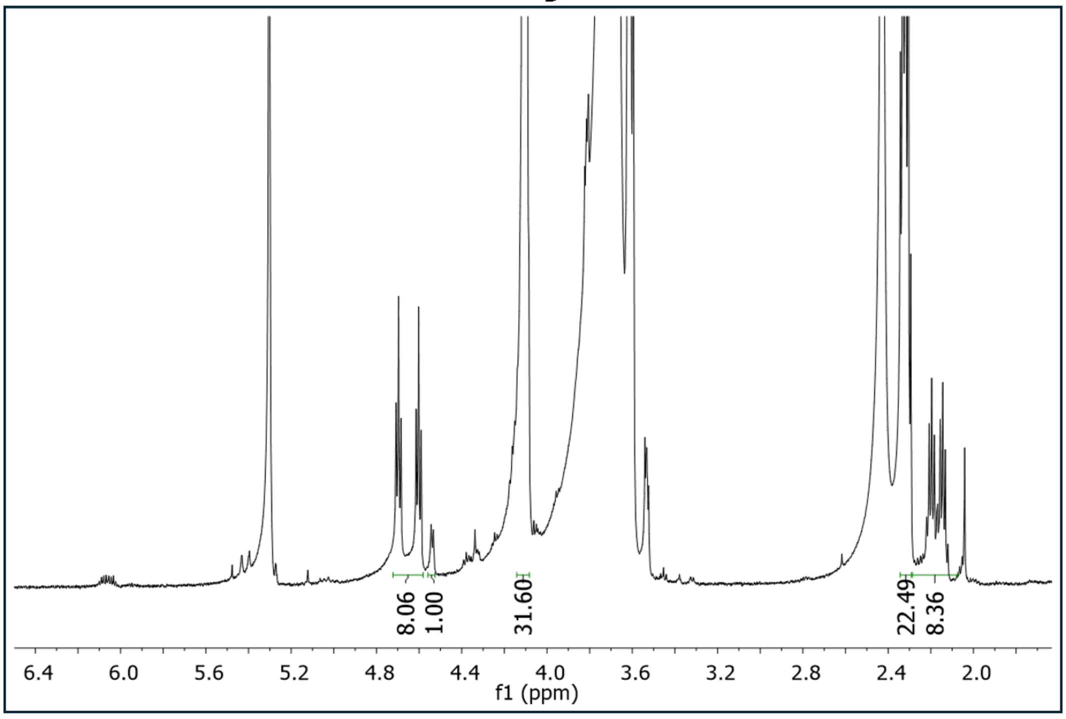

18C6 (1 mmol)
tBuOH (6 mmol), MeCN, reflux, **2h**

Entry 14

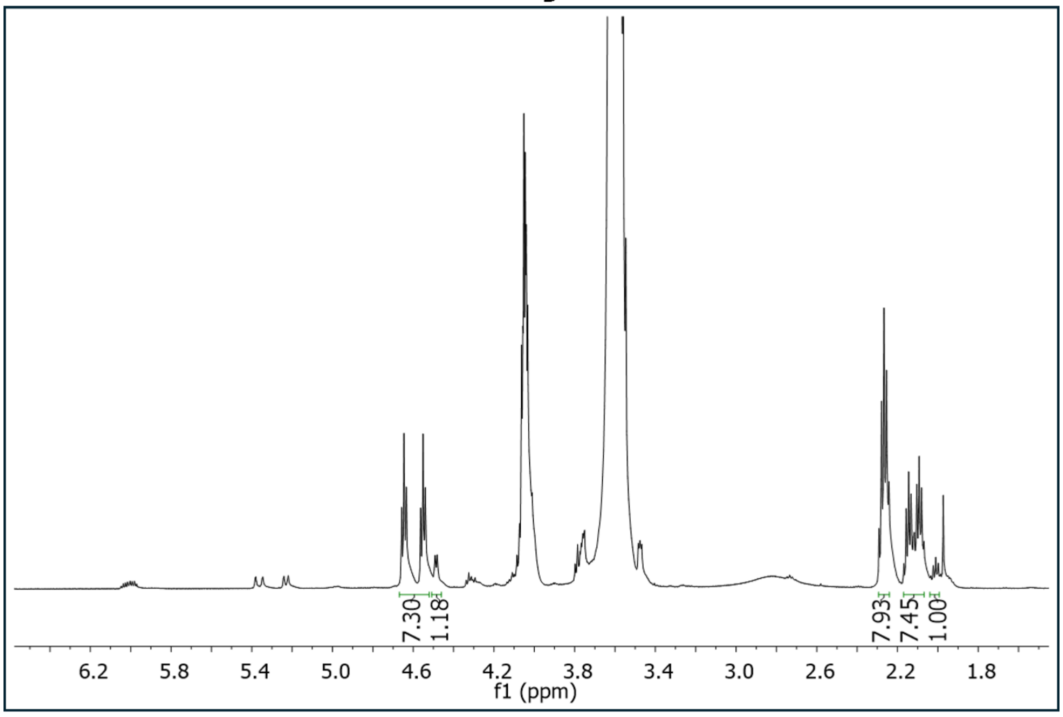

18C6 (1 mmol)
tBuOH (6 mmol), MeCN, reflux, **4h**

Entry 15

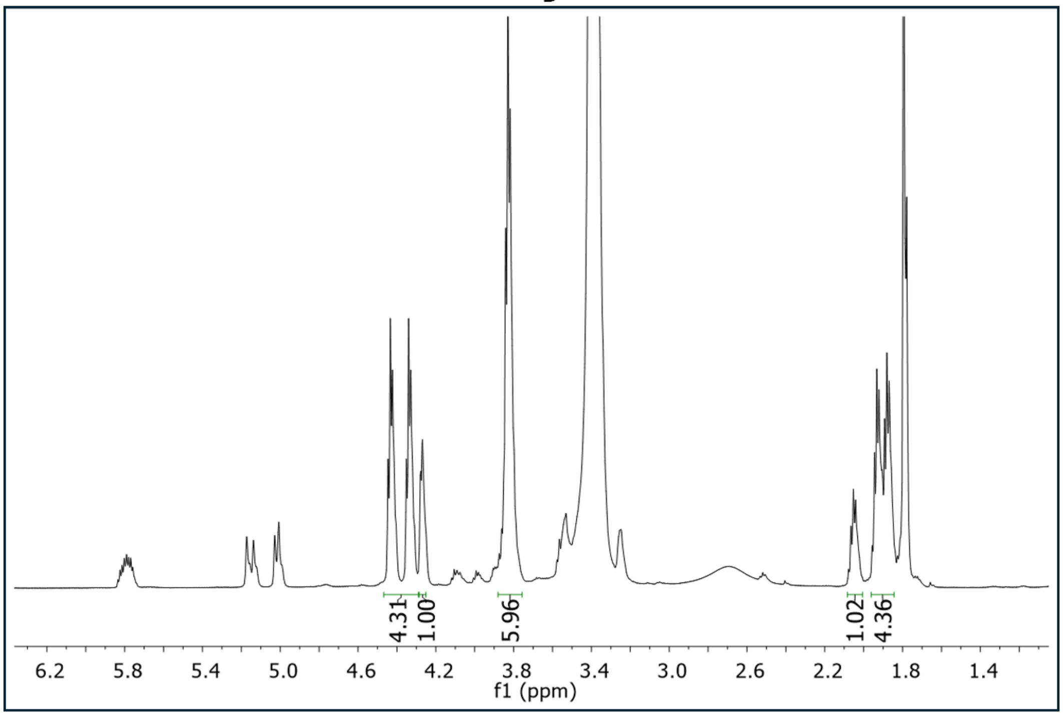

18C6 (1 mmol)
tBuOH (6 mmol), MeCN, reflux, **24h**

Figure S4. ^{1}H NMR ($CDCl_3$, 500 MHz) spectra of fluorination reaction 4 (1 mmol of RBr, 1 mmol of 18C6, 6 mmol of TBOH and 2 mmol of KF) in 2, 4, and 24 h, Entries 13-15

Entry 16

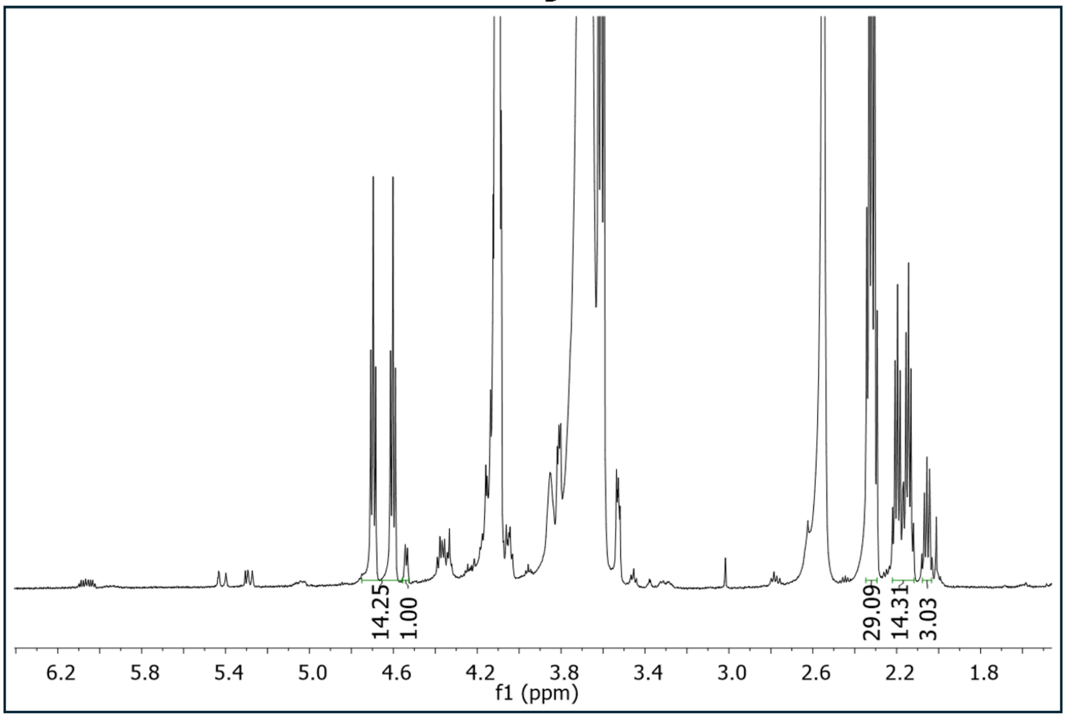

18C6 (1 mmol)
TBOH-F3 (3 mmol), MeCN, reflux, **2h**

Entry 17

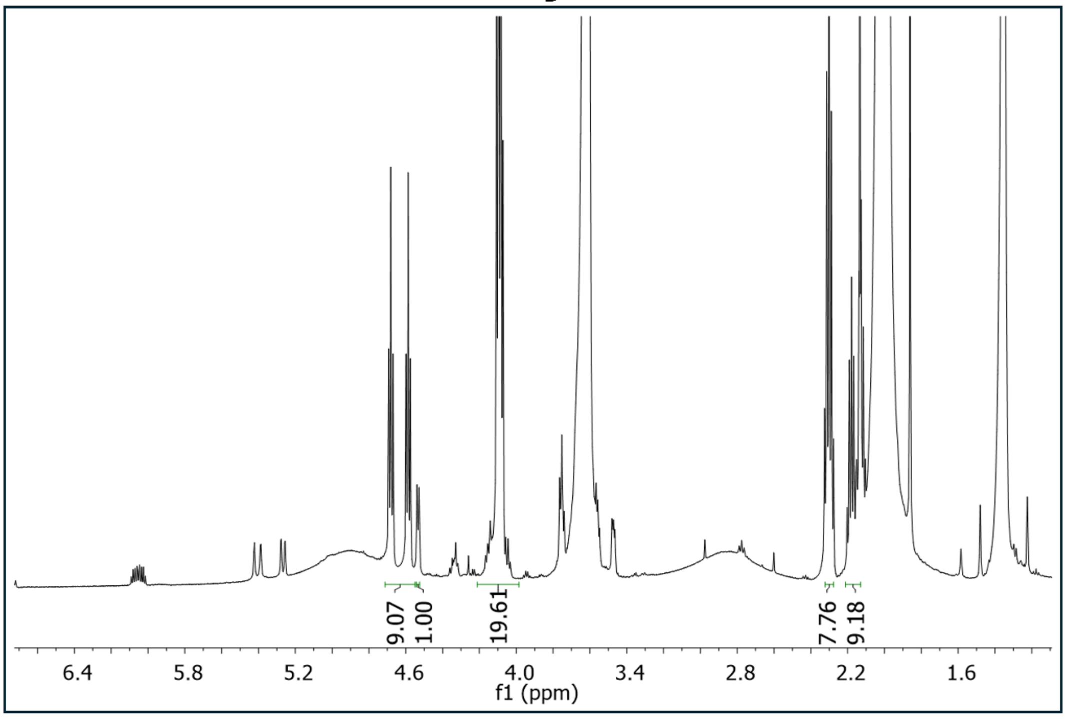

18C6 (1 mmol)
TBOH-F3 (3 mmol), MeCN, reflux, **4h**

Entry 18

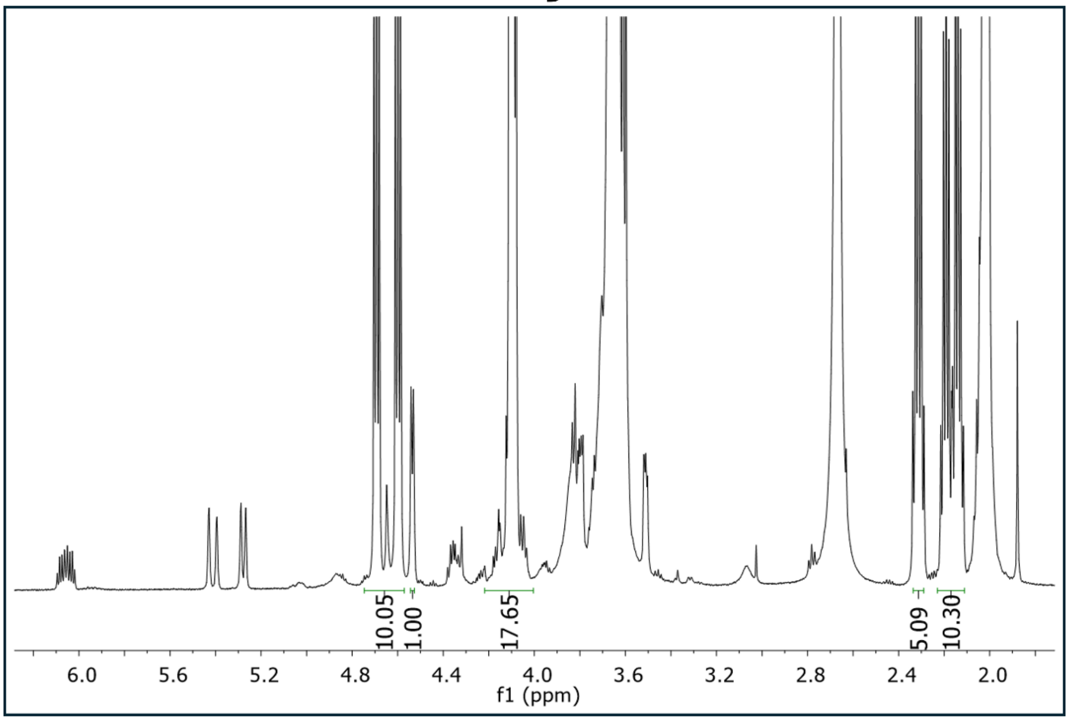

18C6 (1 mmol)
TBOH-F3 (3 mmol), MeCN, reflux, **8h**

Entry 19

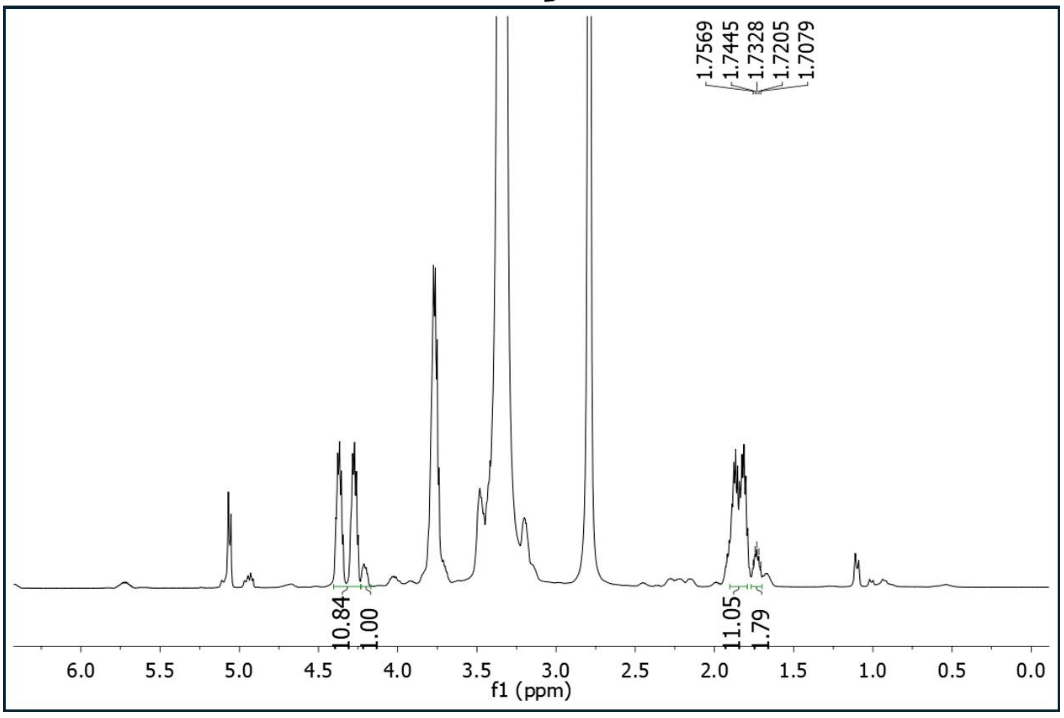

18C6 (1 mmol)
TBOH-F3 (3 mmol), MeCN, reflux, **24h**

Figure S5. ^{1}H NMR ($CDCl_3$, 500 MHz) spectra of fluorination reaction 5 (1 mmol of RBr, 1 mmol of 18C6, 3 mmol of TBOH-F3 and 2 mmol of KF) in 2, 4, 8 and 24 h, , Entries 16-19

Entry 20

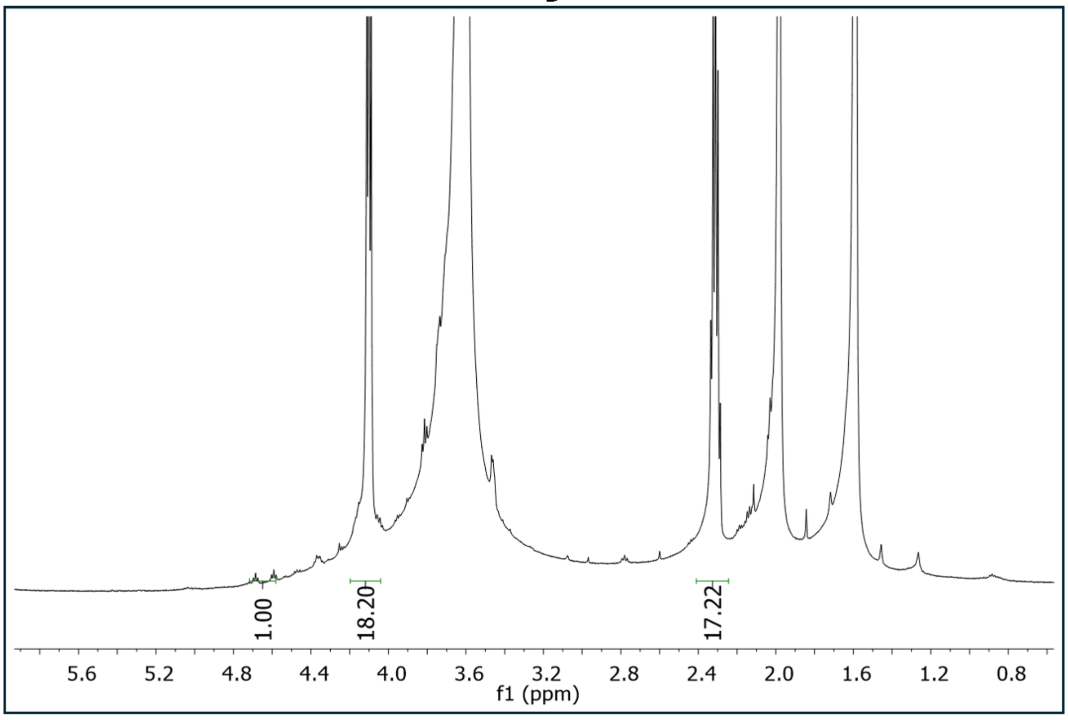

18C6 (1 mmol)

TBOH-F6 (3 mmol), MeCN, reflux, **2h**

Entry 21

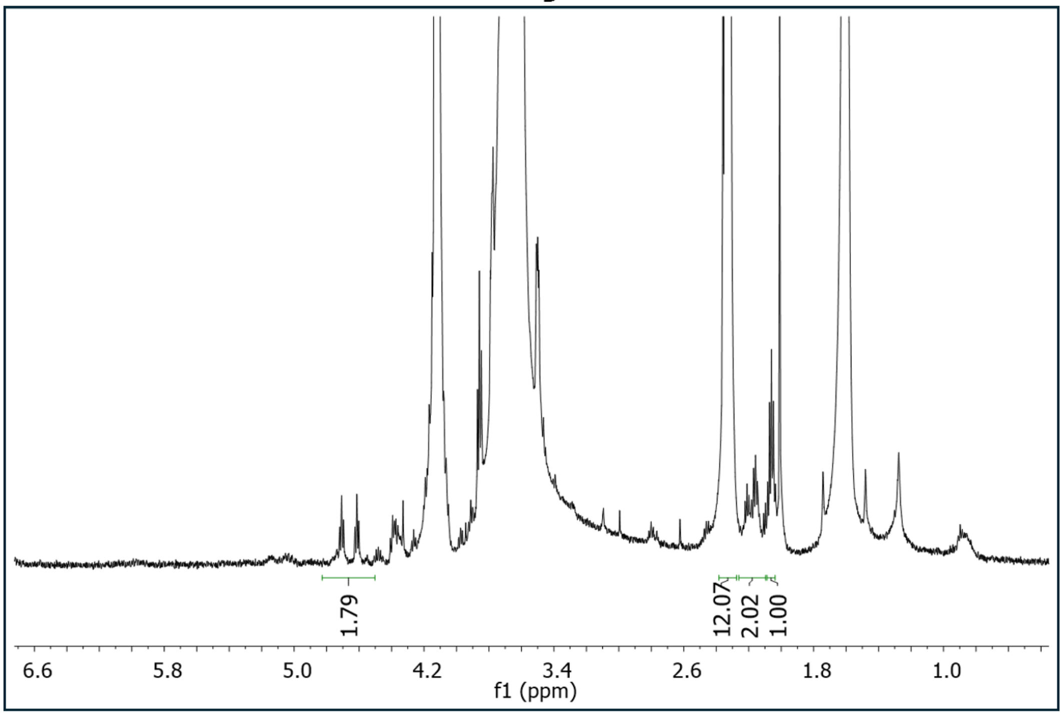

18C6 (1 mmol)

TBOH-F6 (3 mmol), MeCN, reflux, **4h**

Entry 22

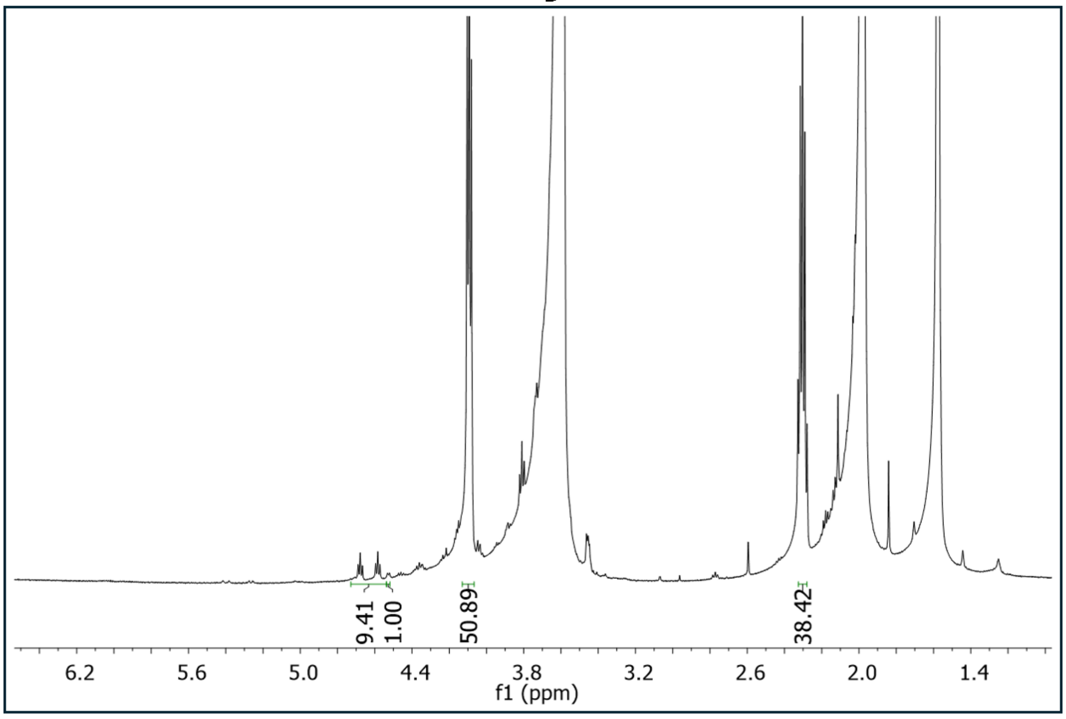

18C6 (1 mmol)
TBOH-F6 (3 mmol), MeCN, reflux, **6h**

Entry 23

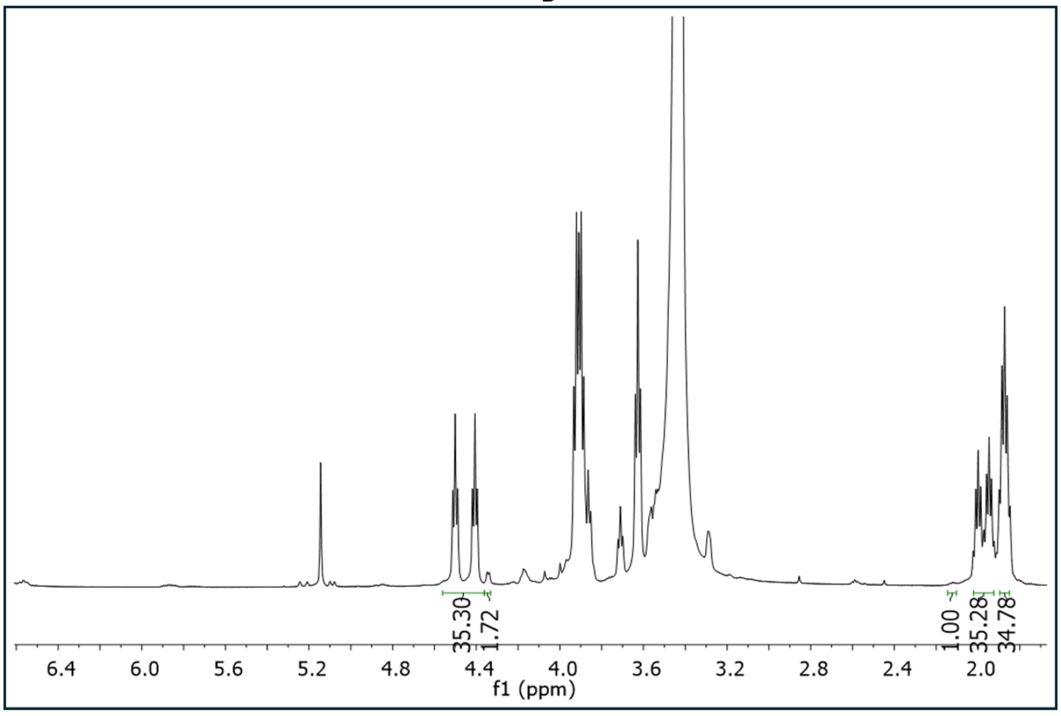

18C6 (1 mmol)
TBOH-F6 (3 mmol), MeCN, reflux, **24h**

Figure S6. ^{1}H NMR ($CDCl_3$, 500 MHz) spectra of fluorination reaction 6 (1 mmol of RBr, 1 mmol of 18C6, 3 mmol of TBOH-F6 and 2 mmol of KF) in 2, 4, 6 and 24 h, Entries 20-23

Entry 24a

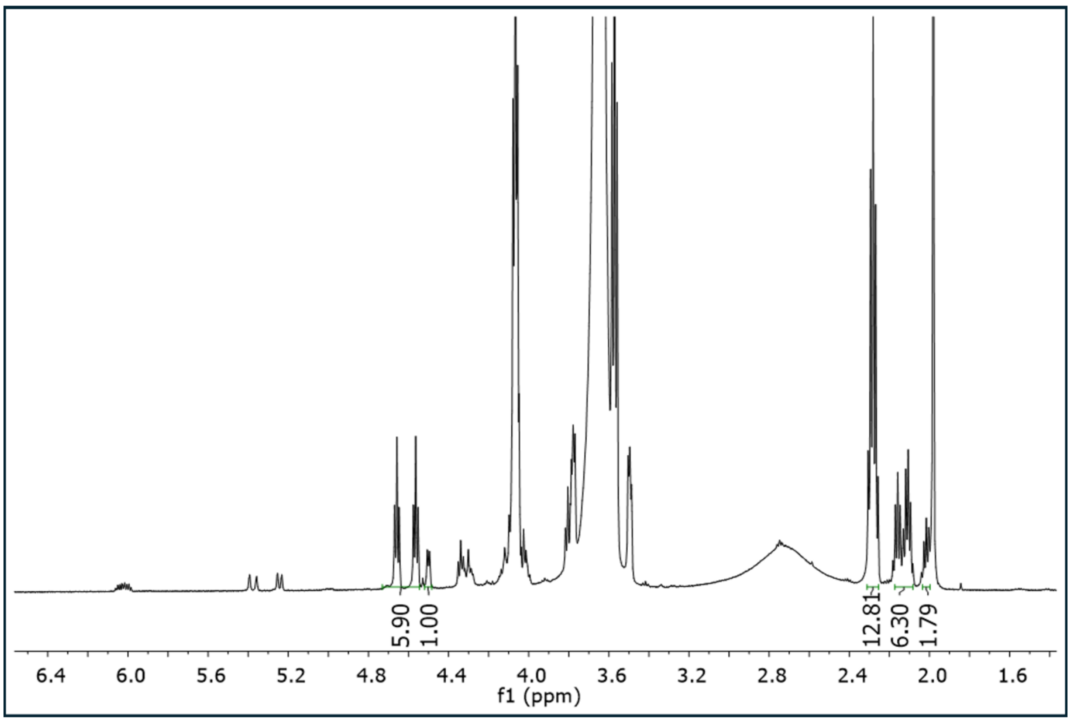

18C6 (2 mmol)
No alcohol, MeCN, reflux, **2h (A)**

Entry 24b

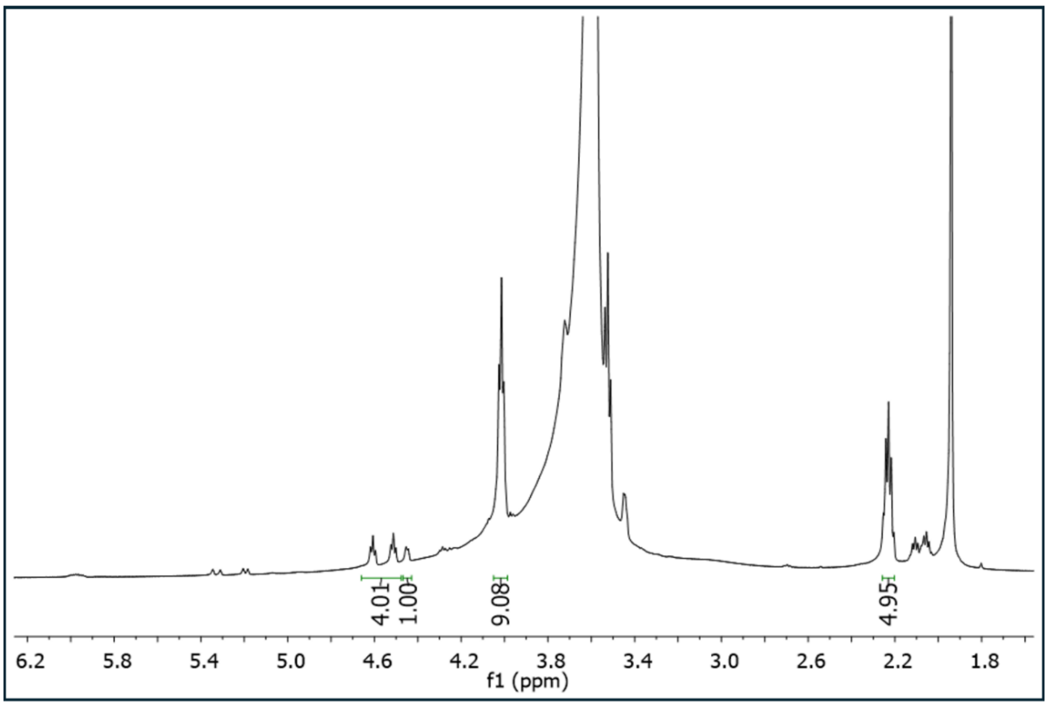

18C6 (2 mmol)
No alcohol, MeCN, reflux, **2h (B)**

Entry 25a

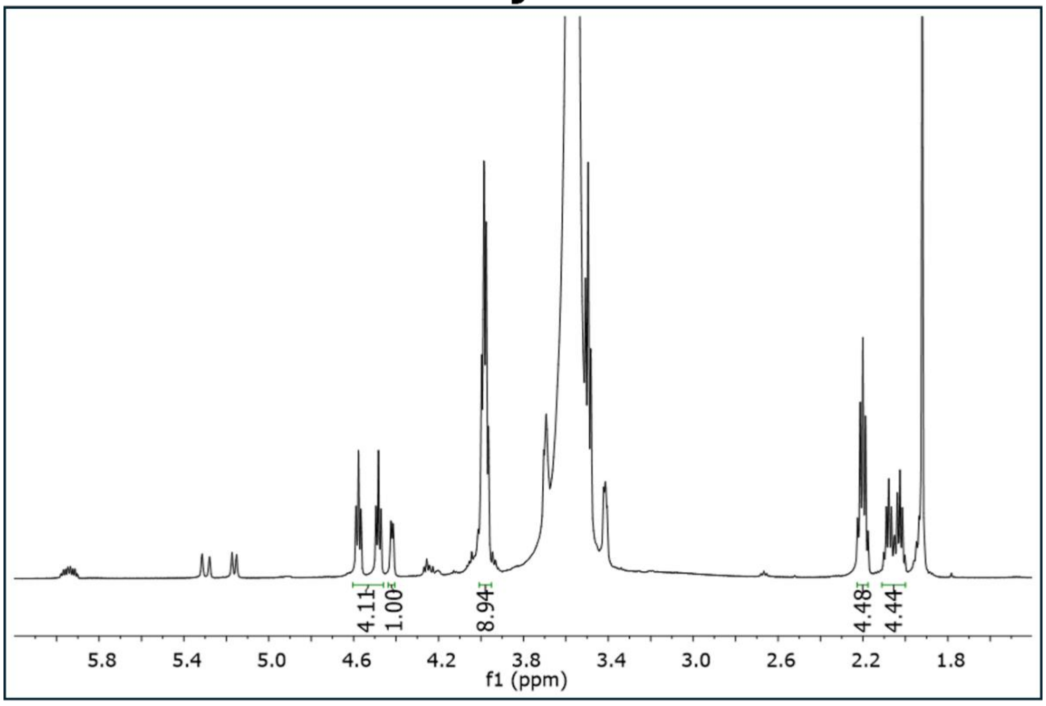

18C6 (2 mmol)

No alcohol, MeCN, reflux, **4h (A)**

Entry 25b

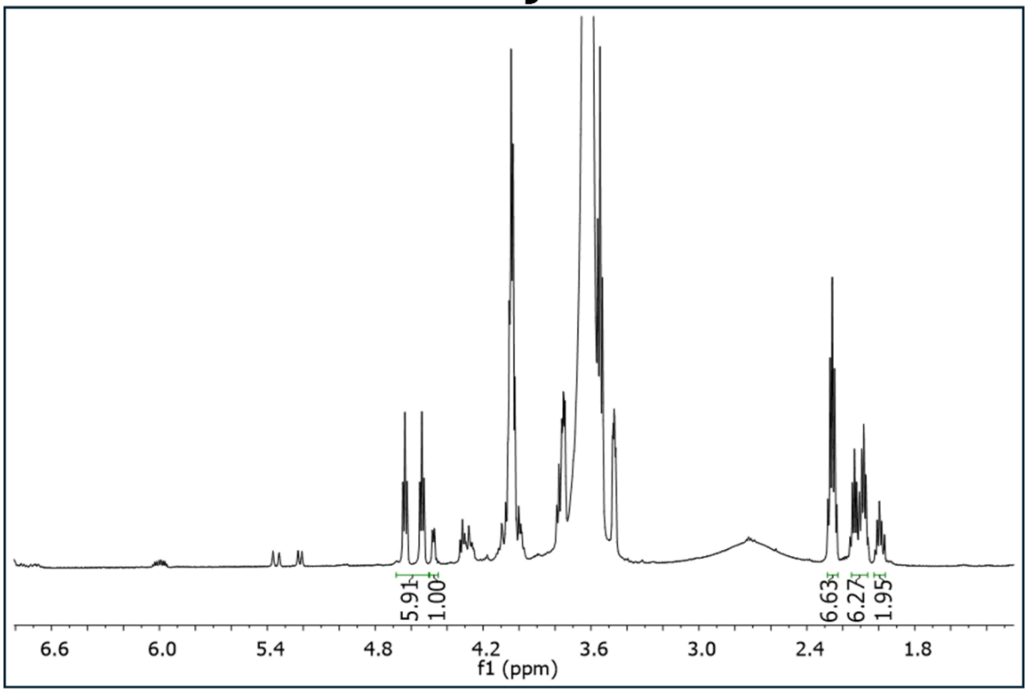

18C6 (2 mmol)

No alcohol, MeCN, reflux, **4h (B)**

Entry 26

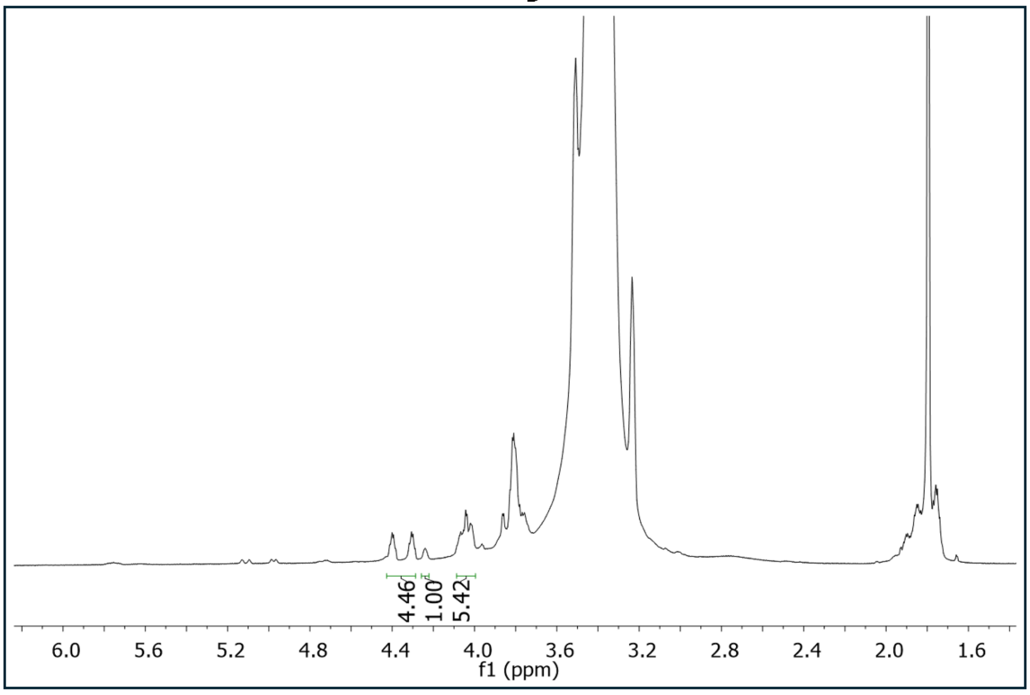

18C6 (2 mmol)
No alcohol, MeCN, reflux, **22h**

Figure S7. ^{1}H NMR ($CDCl_3$, 500 MHz)spectra of fluorination reaction 7 (1 mmol of RBr, 2 mmol of 18C6, and 2 mmol of KF) in 2, 4 and 22 h, Entries 24-26

Entry 27

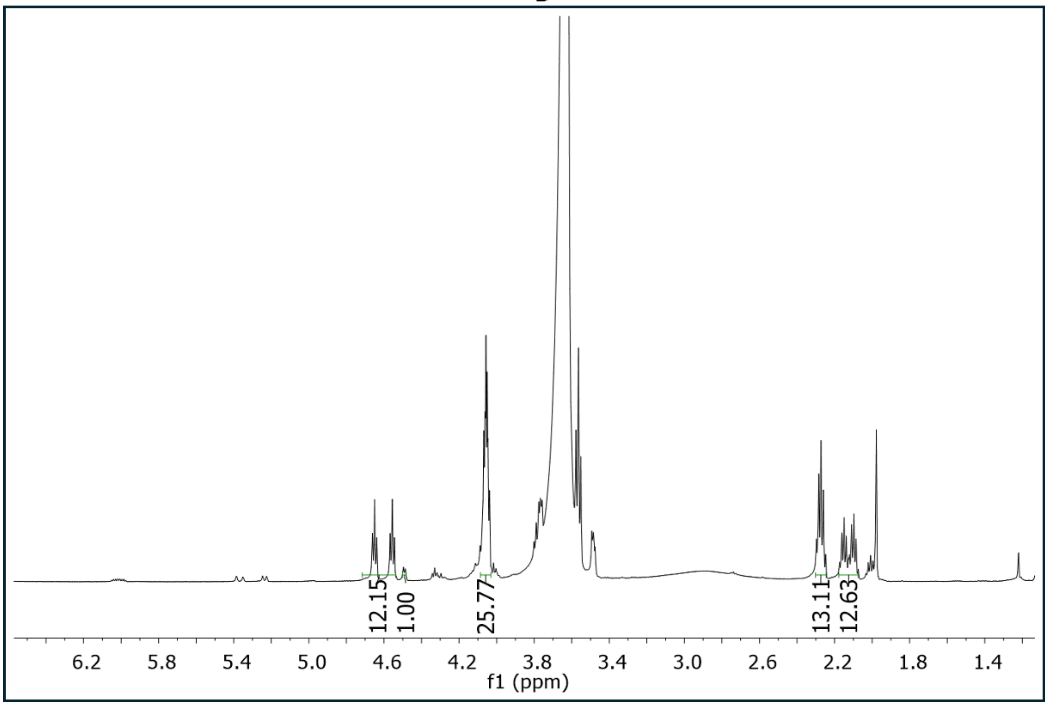

18C6 (2 mmol)
tBuOH (6 mmol), MeCN, reflux, **2h**

Entry 28

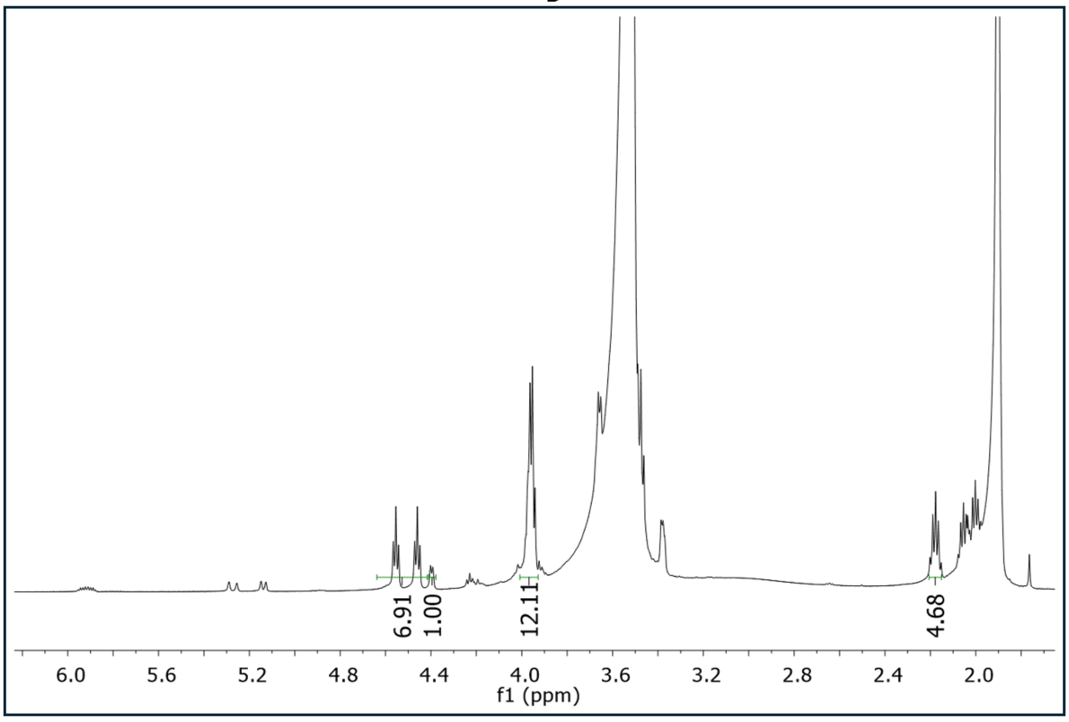

18C6 (2 mmol)
tBuOH (6 mmol), MeCN, reflux, **4h**

Entry 29

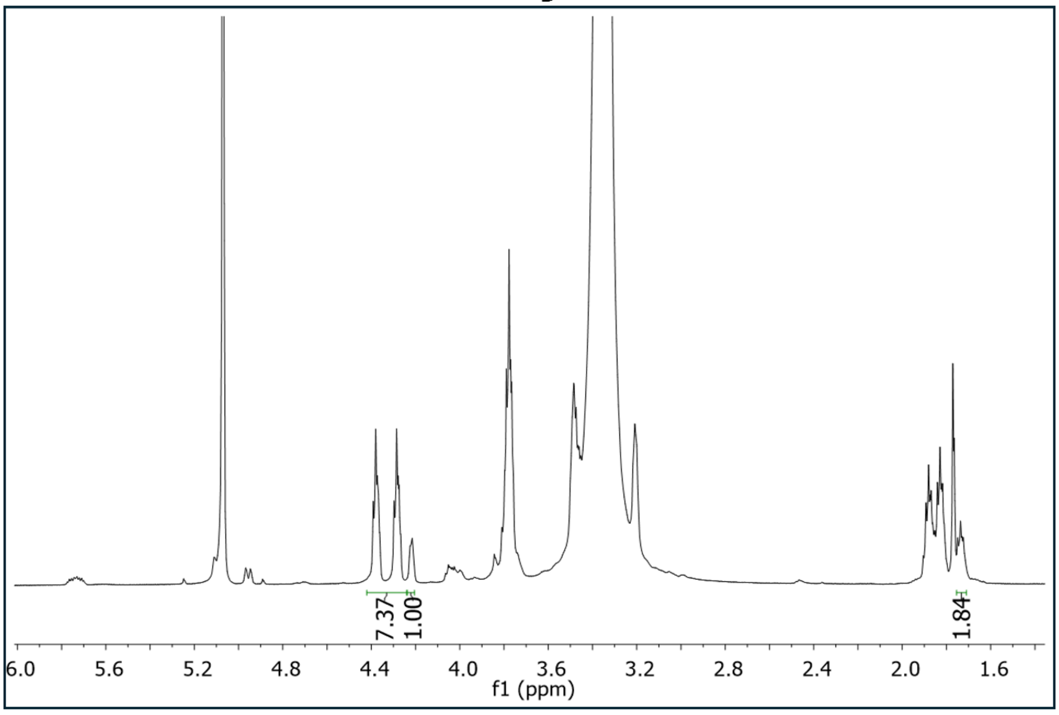

18C6 (2 mmol)
tBuOH (6 mmol), MeCN, reflux, **24h**

Figure S8. ^{1}H NMR ($CDCl_3$, 500 MHz)spectra of fluorination reaction 8 (1 mmol of RBr, 2 mmol of 18C6, 6 mmol of TBOH and 2 mmol of KF) in 2, 4 and 24 h. Entries 27-29

Entry 30

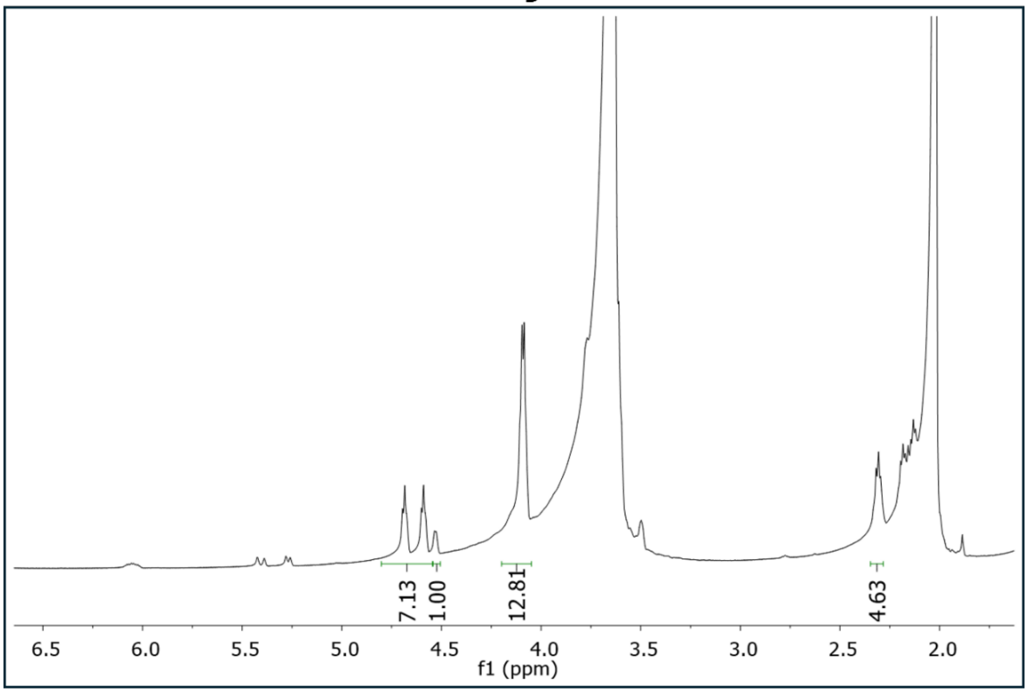

18C6 (2 mmol)
TBOH-F3 (3 mmol), MeCN, reflux, **2h**

Entry 31

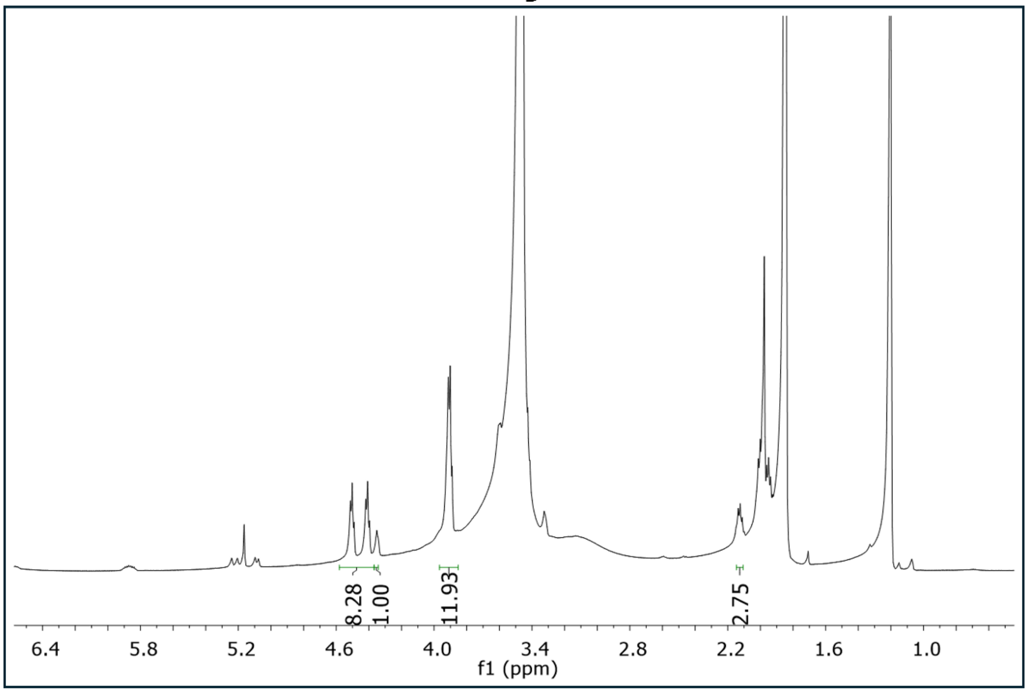

18C6 (2 mmol)
TBOH-F3 (3 mmol), MeCN, reflux, **4h**

Entry 32

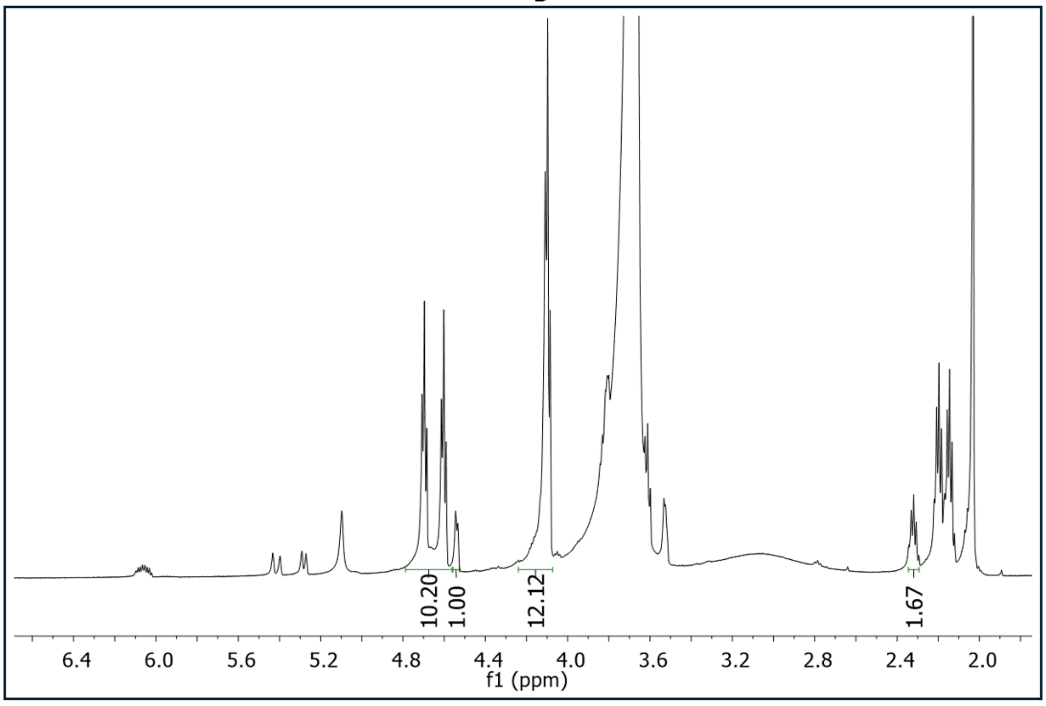

18C6 (2 mmol)
TBOH-F3 (3 mmol), MeCN, reflux, **6h**

Entry 33

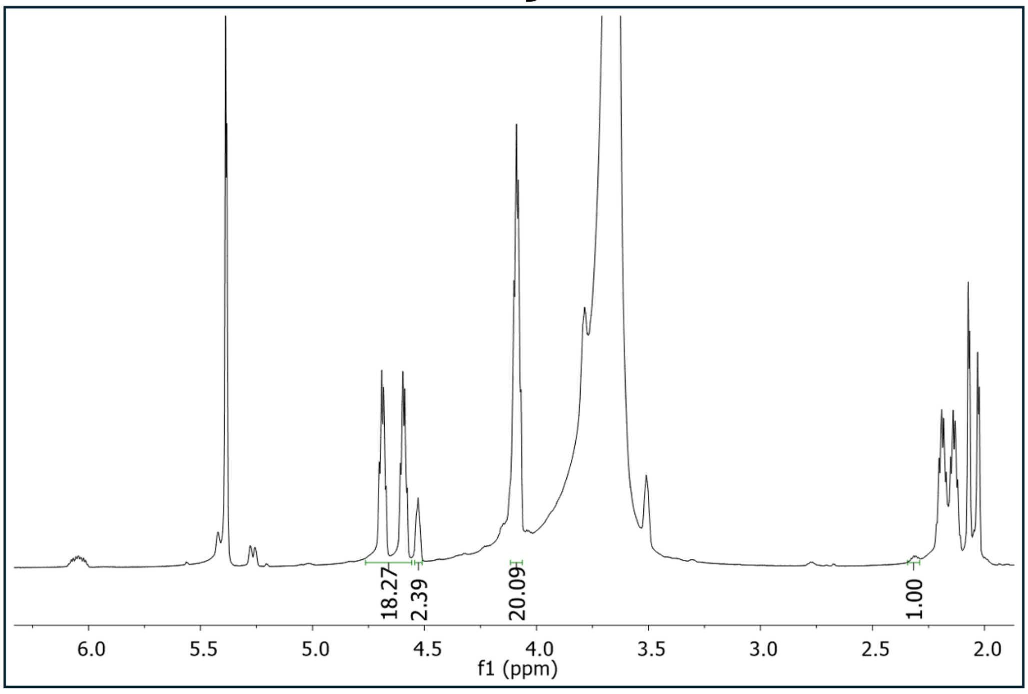

18C6 (2 mmol)
TBOH-F3 (3 mmol), MeCN, reflux, **24h**

Figure S9. ^{1}H NMR ($CDCl_3$, 500 MHz)spectra of fluorination reaction 9 (1 mmol of RBr, 2 mmol of 18C6, 3 mmol of TBOH-F3 and 2 mmol of KF) in 2, 4, 6 and 24 h, Entries 30-33

Entry 34

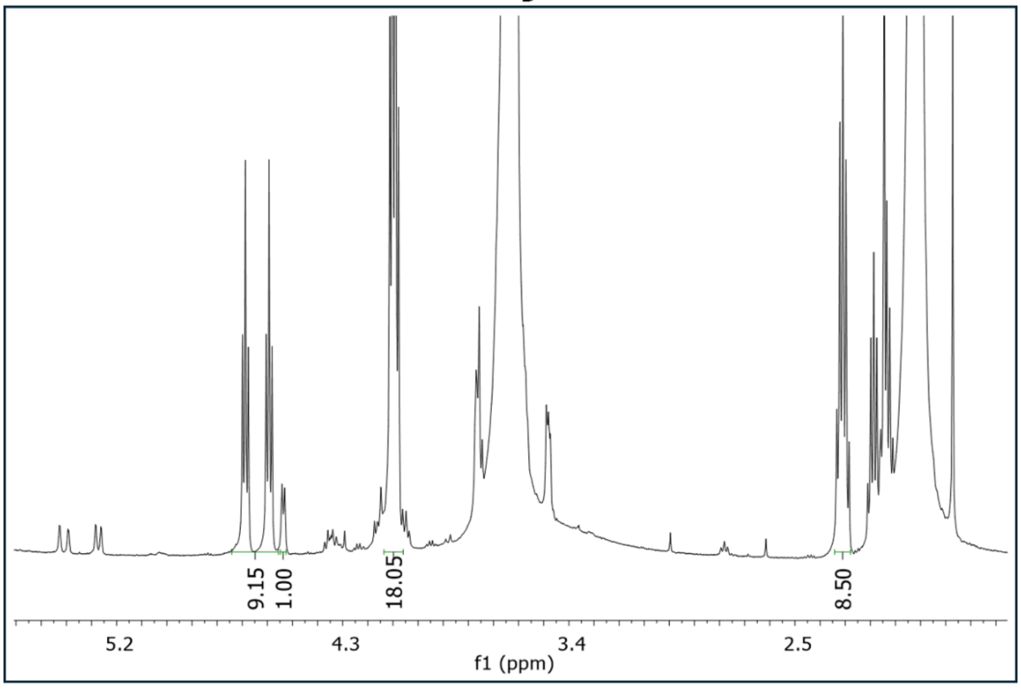

18C6 (2 mmol)
TBOH-F3 (6mmol), MeCN, reflux, **2h**

Entry 35

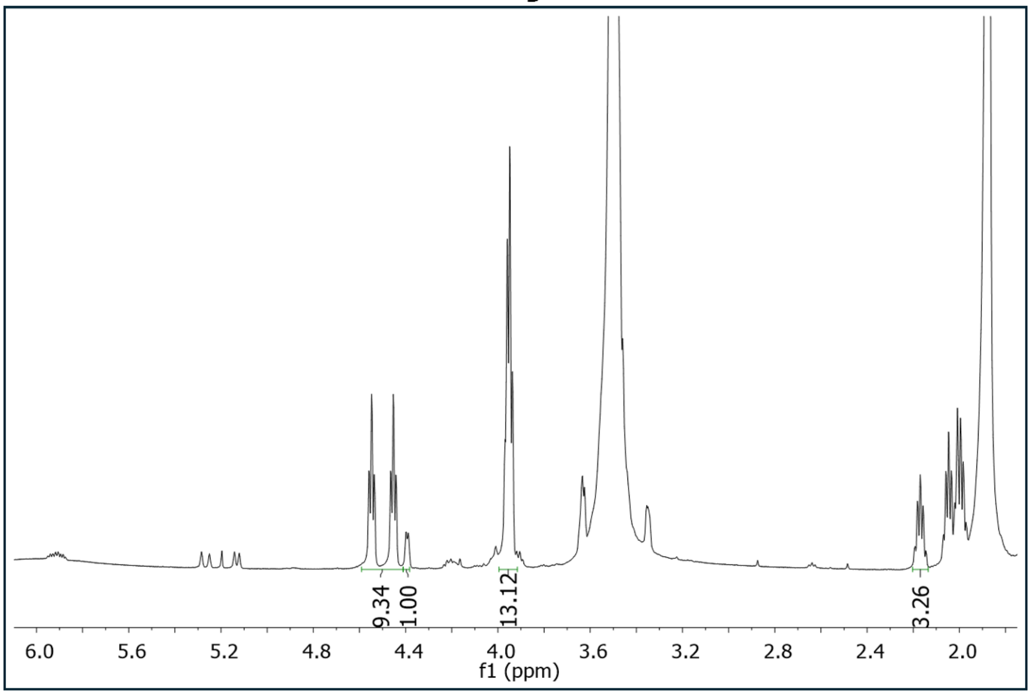

18C6 (2 mmol)
TBOH-F3 (6mmol), MeCN, reflux, **4h**

Entry 36

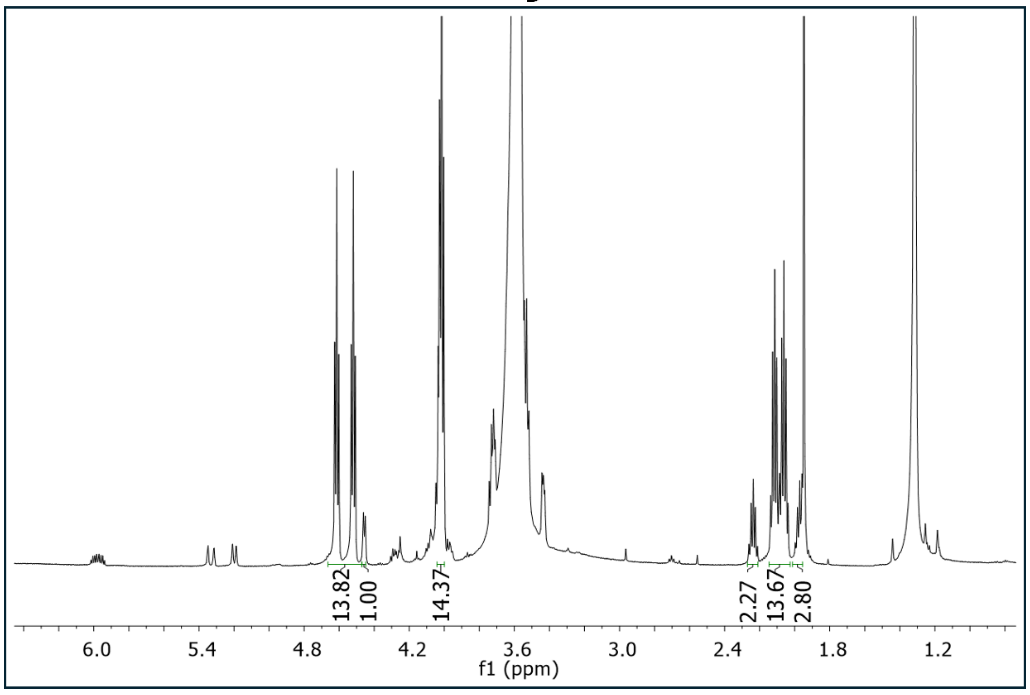

18C6 (2 mmol)
TBOH-F3 (6mmol), MeCN, reflux, **6h**

Entry 37

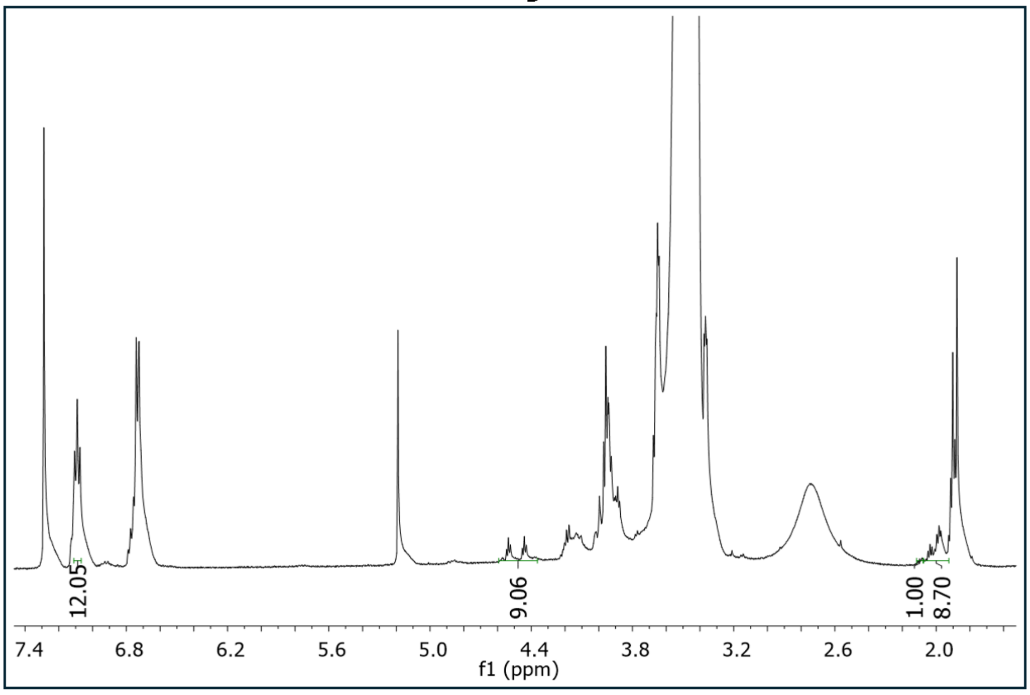

18C6 (2 mmol)
TBOH-F3 (6mmol), MeCN, reflux, **24h**

Figure S10. ^{1}H NMR ($CDCl_3$, 500 MHz) spectra of fluorination reaction 10 (1 mmol of RBr, 2 mmol of 18C6, 6 mmol of TBOH-F3 and 2 mmol of KF) in 2, 4, 6 and 24 h, Entries 34-37

Entry 38

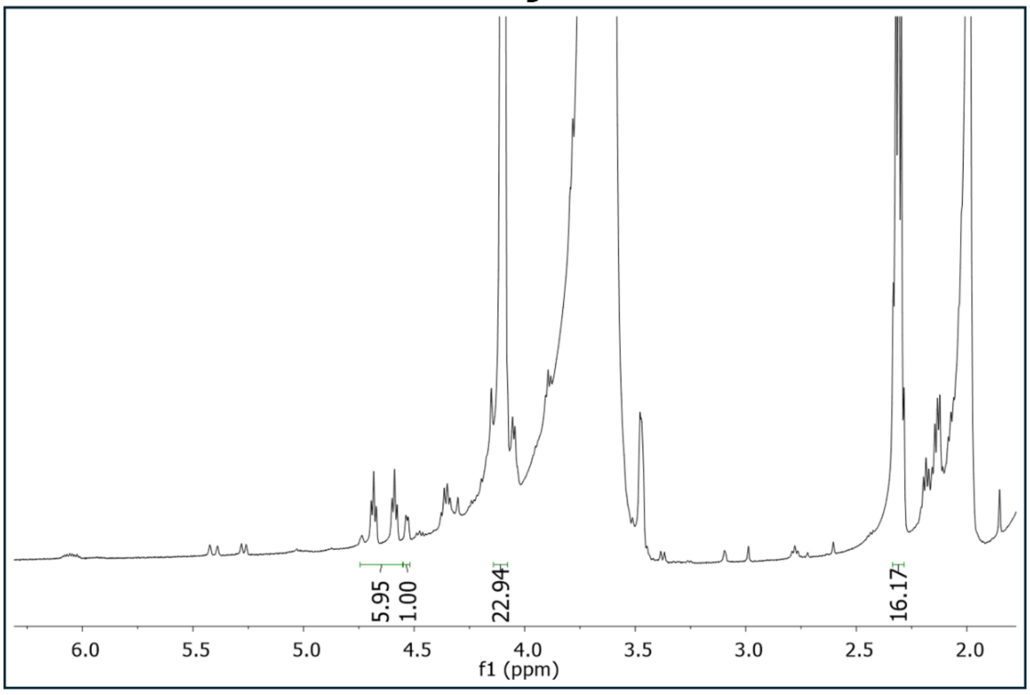

18C6 (2 mmol)
TBOH-F6 (3 mmol), MeCN, reflux, **2h**

Entry 39

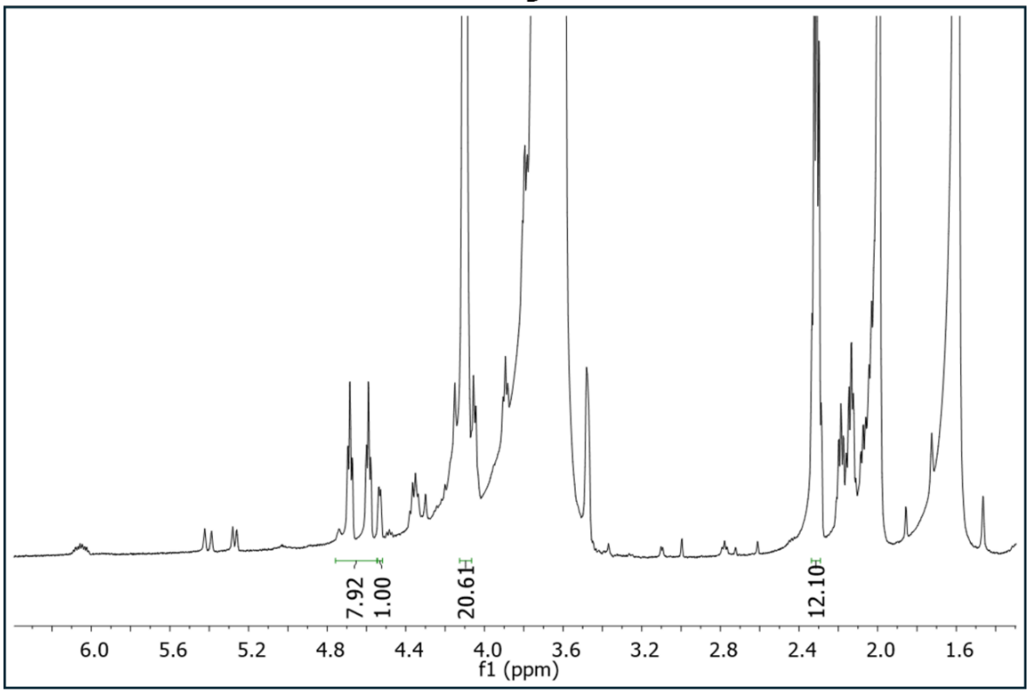

18C6 (2 mmol)
TBOH-F6 (3 mmol), MeCN, reflux, **4h**

Entry 40

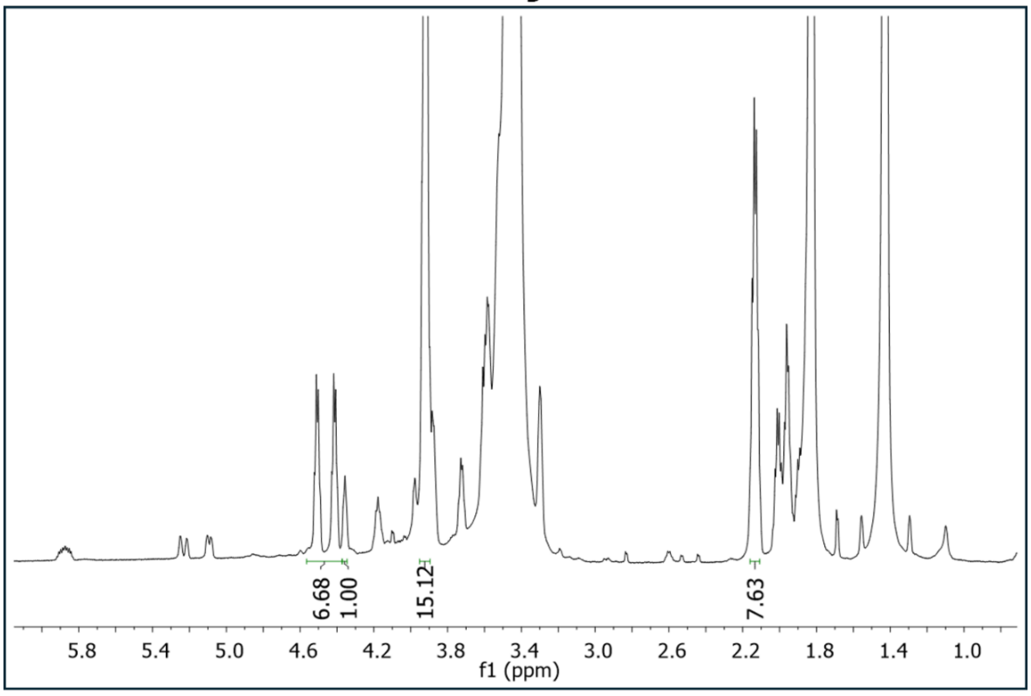

18C6 (2 mmol)
TBOH-F6 (3 mmol), MeCN, reflux, **6h**

Entry 41

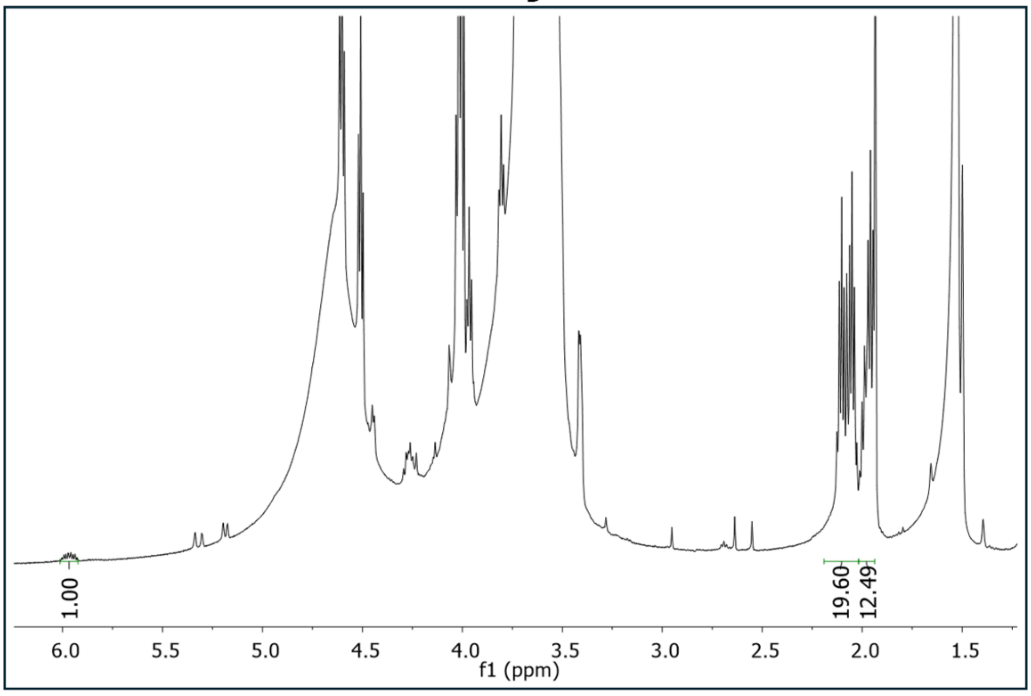

18C6 (2 mmol)
TBOH-F6 (3 mmol), MeCN, reflux, **24h**

Figure S11. ^{1}H NMR ($CDCl_3$, 500 MHz) spectra of fluorination reaction 11 (1 mmol of RBr, 2 mmol of 18C6, 3 mmol of TBOH-F6 and 2 mmol of KF) in 2, 4, 6 and 24 h, Entries 38-41

Entry 42

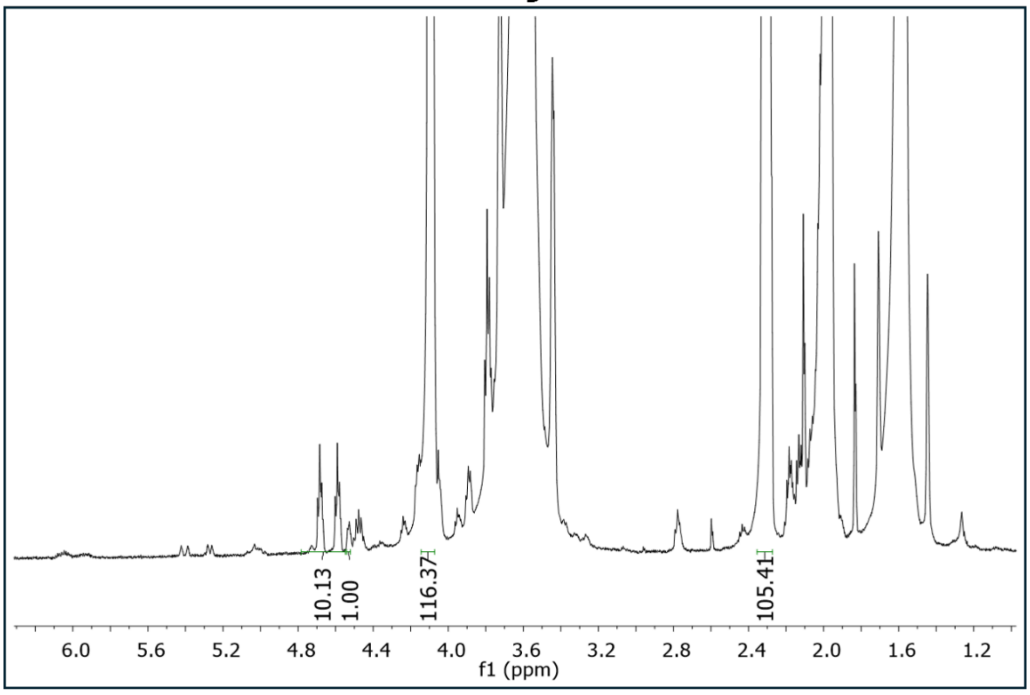

18C6 (2 mmol)
TBOH-F6 (6 mmol), MeCN, reflux, **4h**

Entry 43

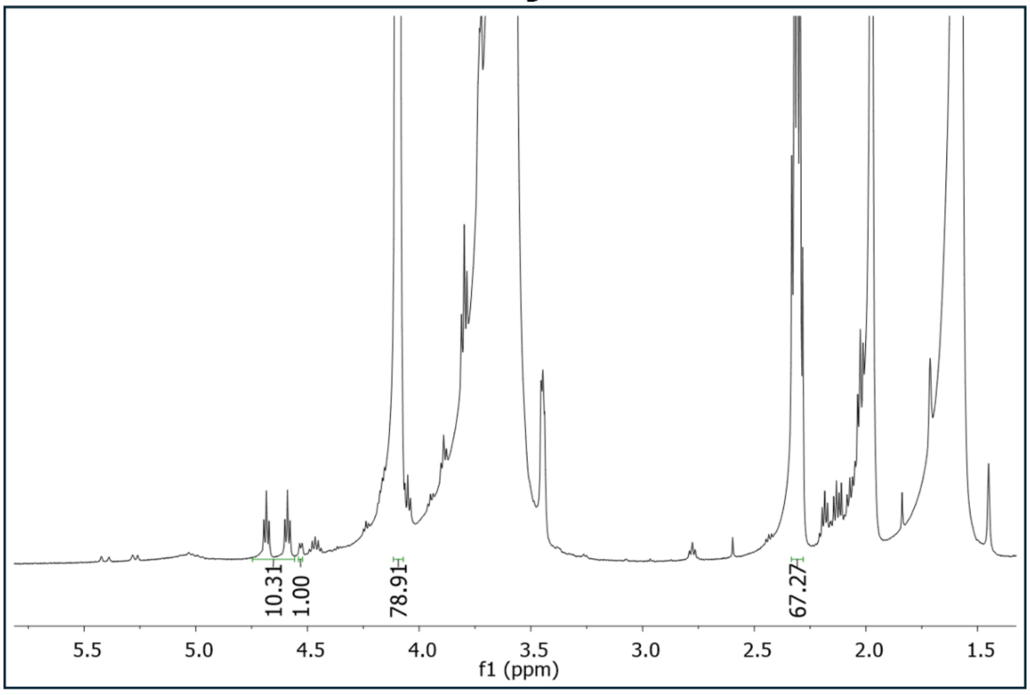

18C6 (2 mmol)
TBOH-F6 (6 mmol), MeCN, reflux, **6h**

Entry 44

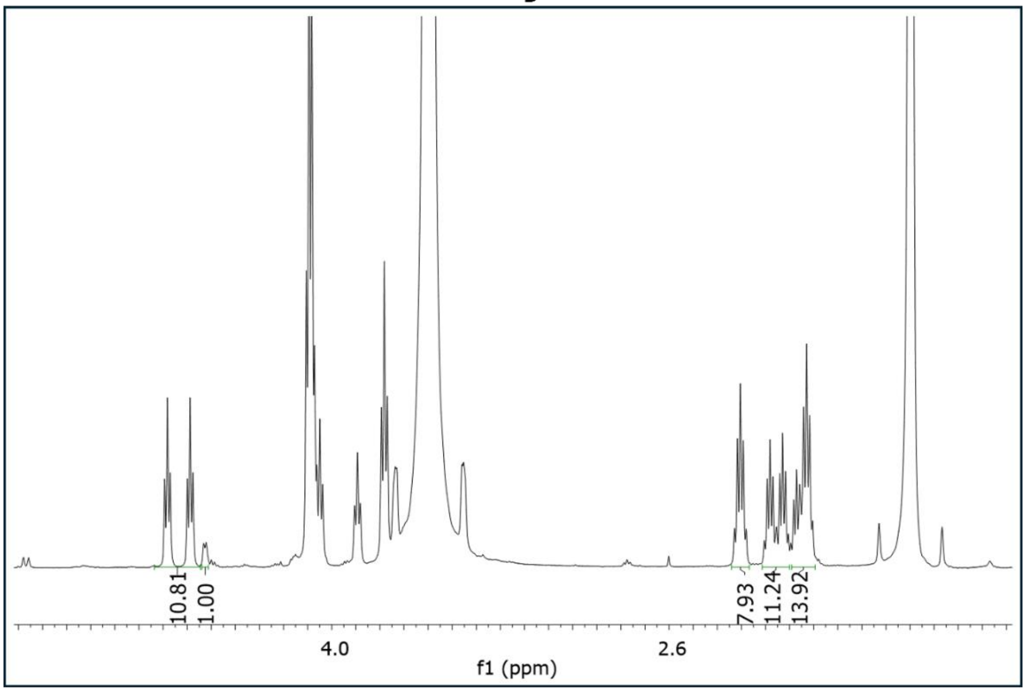

18C6 (2 mmol)
TBOH-F6 (6 mmol), MeCN, reflux, **24h**

Figure S12. ^{1}H NMR ($CDCl_3$, 500 MHz)spectra of fluorination reaction 12 (1 mmol of RBr, 2 mmol of 18C6, 6 mmol of TBOH-F6 and 2 mmol of KF) in 4, 6 and 24 h, Entries 42-44

1.3.2. ^{1}H NMR spectra of the crude reactions of Table S2

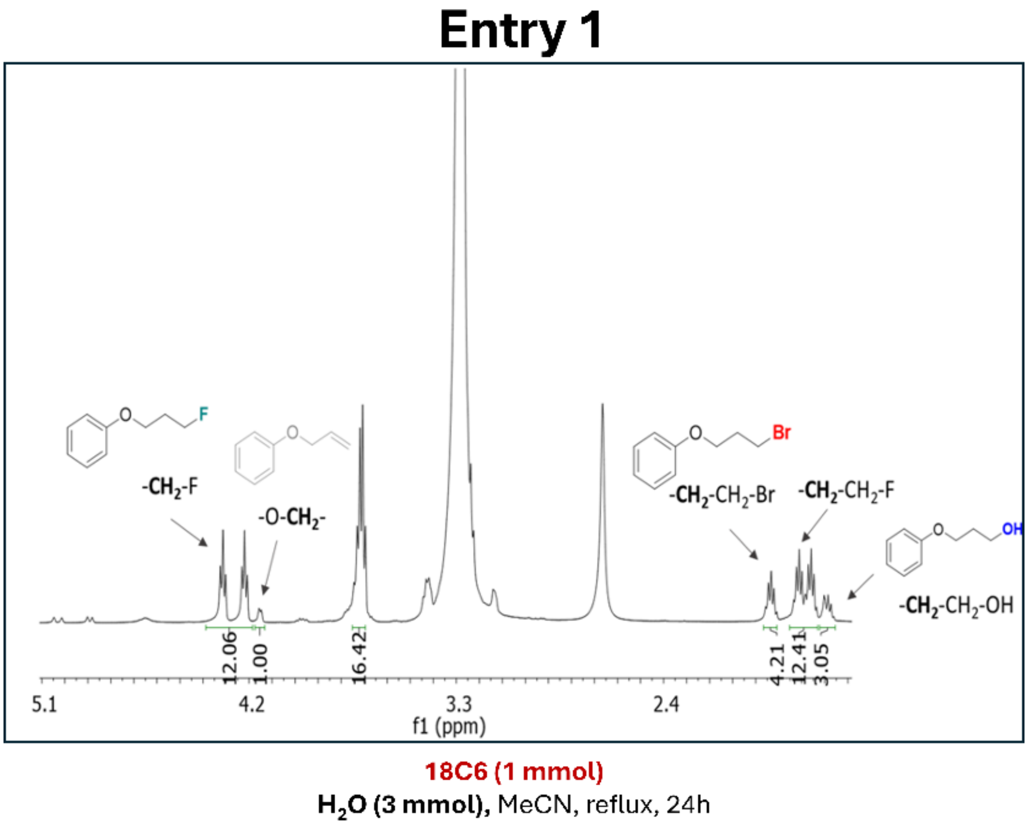

Figure S13. ^{1}H NMR ($CDCl_3$, 500 MHz) spectra of fluorination reaction 13 (1 mmol of RBr, 1 mmol of 18C6, 3 mmol of H_2O and 2 mmol of KF), 24 h

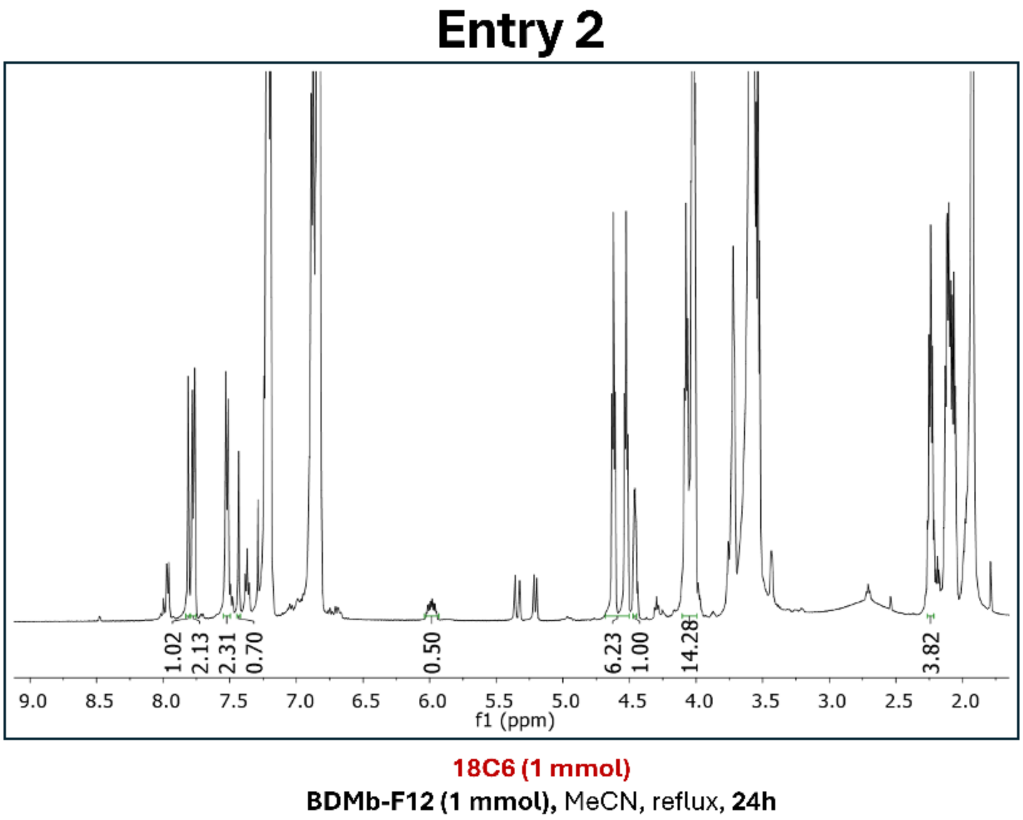

Figure S14. ^{1}H NMR ($CDCl_3$, 500 MHz) spectra of fluorination reaction 14 (1 mmol of RBr, 1 mmol of 18C6, 1 mmol of BDMb-F12 and 2 mmol of KF), 24 h

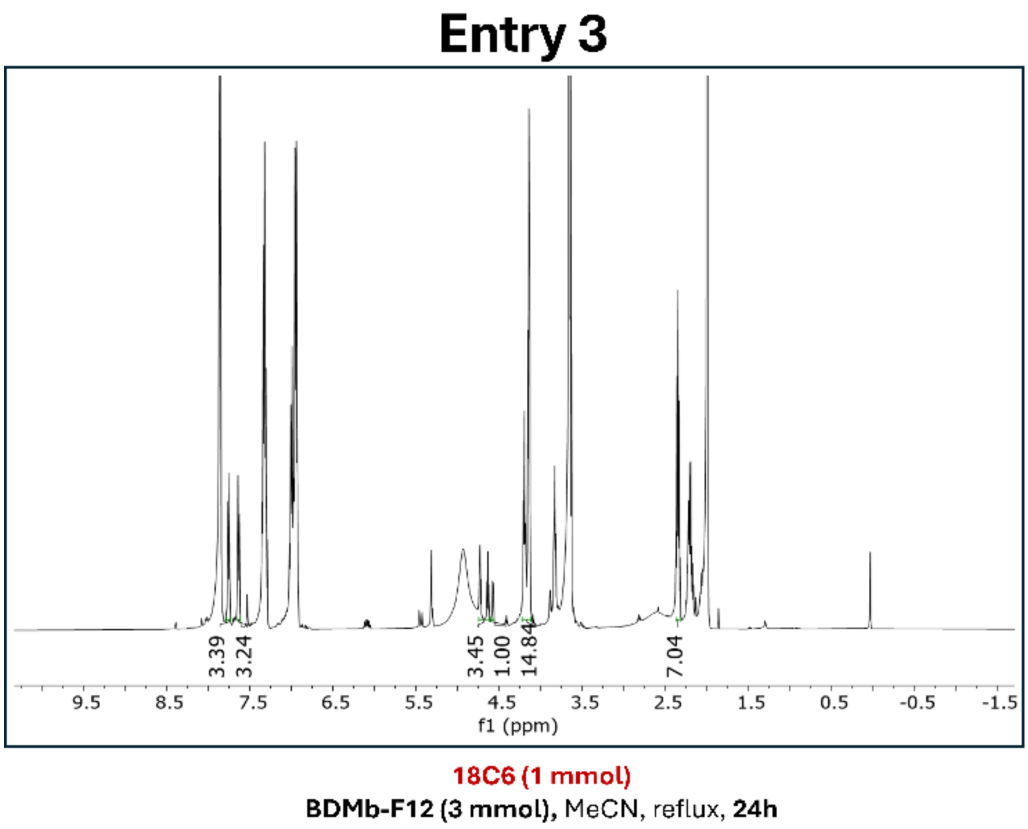

Figure S15. ^{1}H NMR ($CDCl_3$, 500 MHz) spectra of fluorination reaction 15 (1 mmol of RBr, 1 mmol of 18C6, 3 mmol of BDMb-F12 and 2 mmol of KF), 24 h

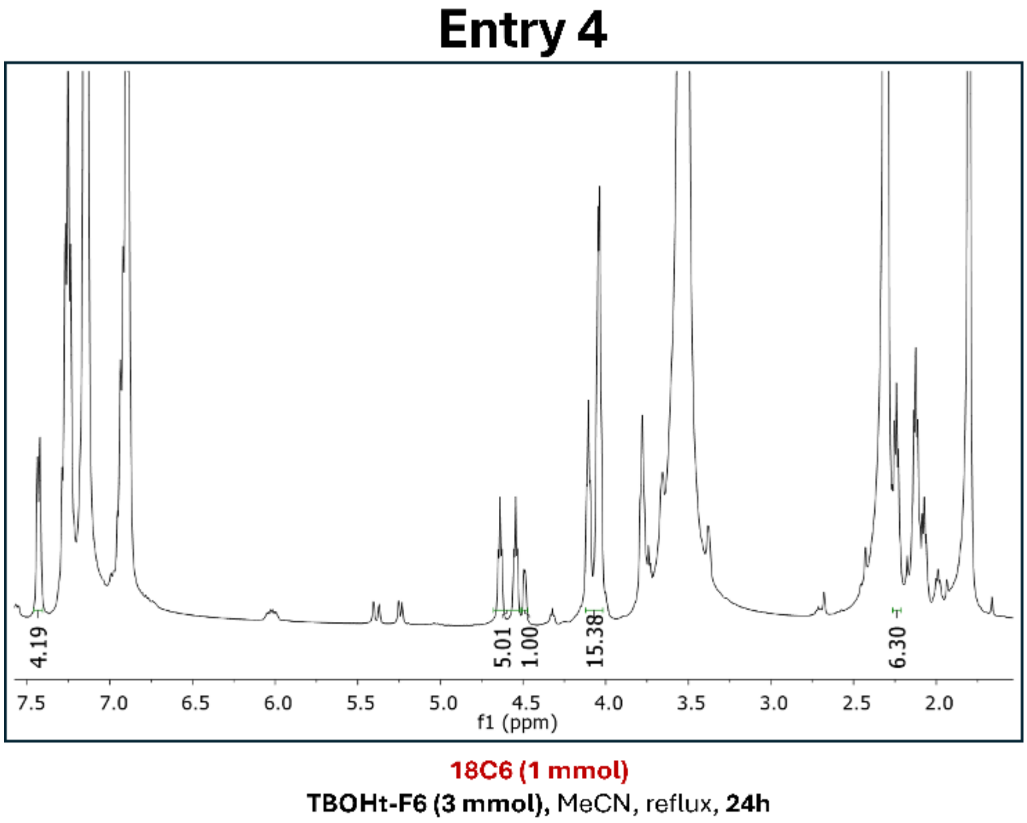

Figure S16. ^{1}H NMR ($CDCl_3$, 500 MHz) spectra of fluorination reaction 16 (1 mmol of RBr, 1 mmol of 18C6, 3 mmol of TBOHt-F6 and 2 mmol of KF), 24 h

Entry 5

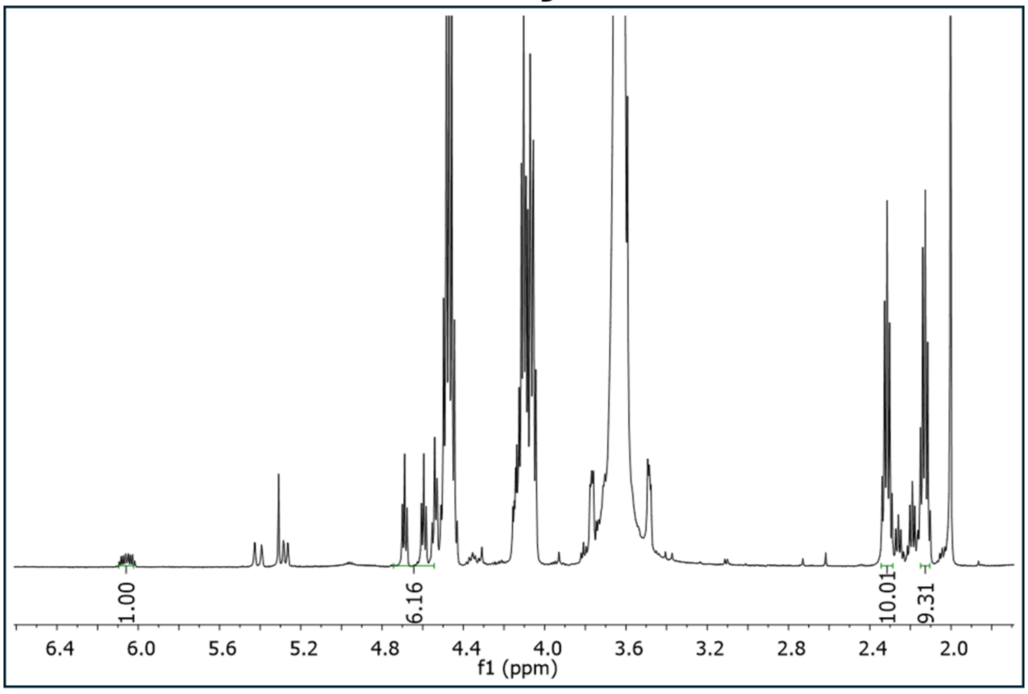

18C6 (2 mmol)
HFIP (3 mmol), MeCN, reflux, **3h**

Figure S17. ^{1}H NMR ($CDCl_3$, 500 MHz) spectra of fluorination reaction 16 (1 mmol of RBr, 1 mmol of 18C6, 3 mmol of HFIP and 2 mmol of KF), 3 h

1.3.3. ^{1}H NMR spectra of the crude reaction of the secondary substrate

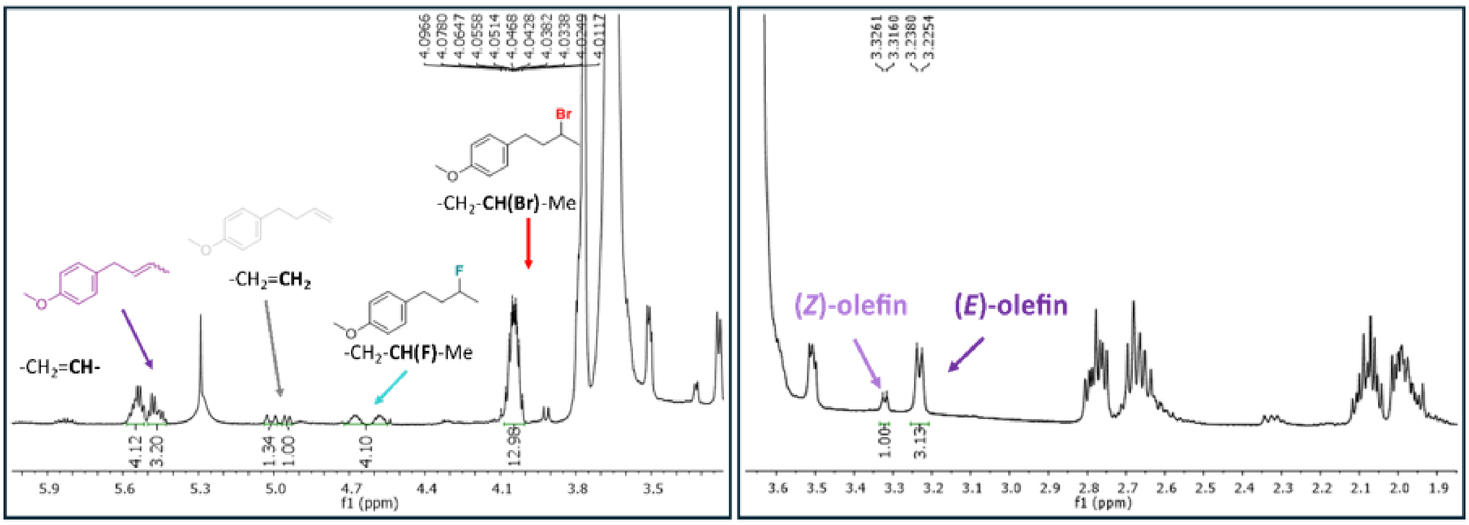

Secondary bromide, 18C6 (2 mmol)
TBOH-F3 (6 mmol), MeCN, reflux, 2h

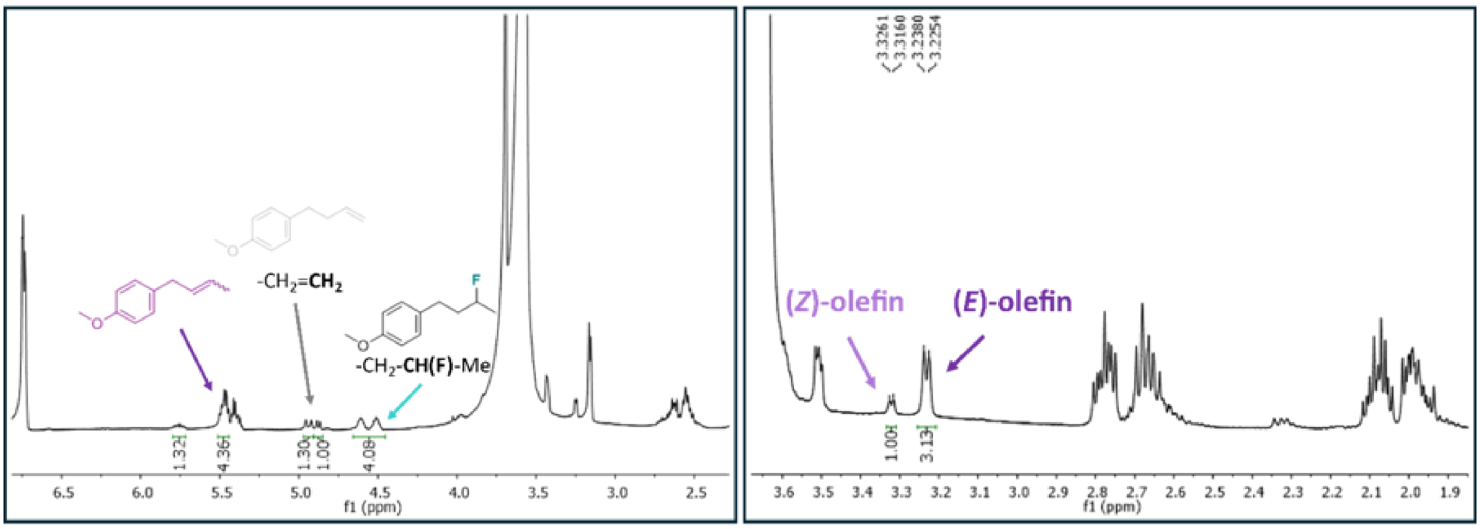

Secondary bromide, 18C6 (2 mmol)
TBOH-F3 (6 mmol), MeCN, reflux, 18h

Figure S18. ^{1}H NMR ($CDCl_3$, 500 MHz) spectra of fluorination reaction of secondary substrate (1 mmol of **7**, 2.0 mmol of 18C6 and 2 mmol of KF) in 2 and 18 h

1.3.4. HRMS spectra of the fluorination reaction mixtures

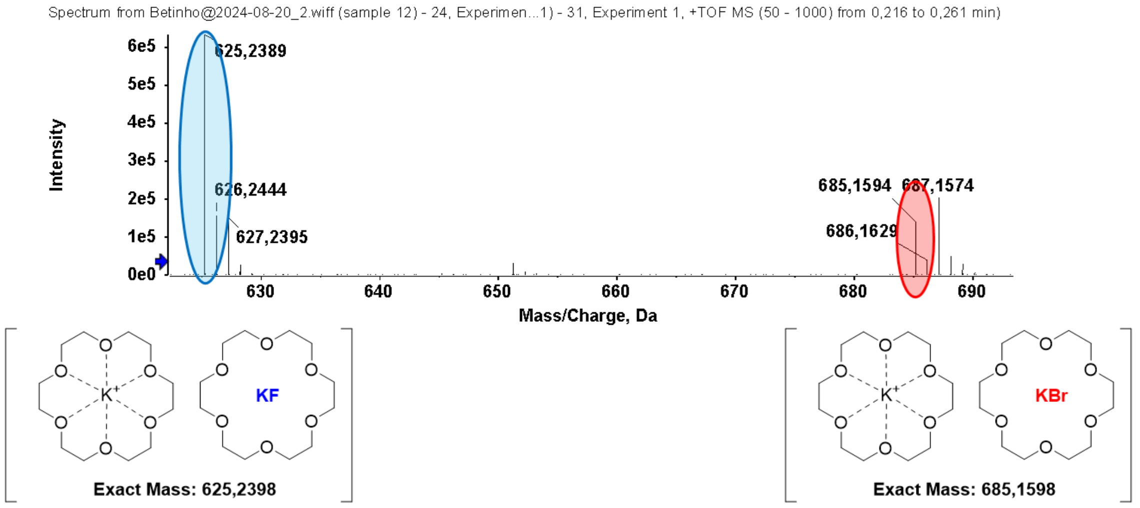

Comment: Both salts, KF and KBr were detected caged and adducted with 18C6, which corroborates with the initial proposal of the output KBr to inhibit the solubilization of KF

Figure S19. HRMS (ESI) m/z: $[M + K + (18C6+KF)]^+$ Calcd for $C_{24}H_{48}FK_2O_{12}^+$ 625.2398; Found 625.2389 (blue), and m/z: $[M + K + (18C6+KBr)]^+$ Calcd for $C_{24}H_{48}FK_2O_{12}^+$ 685.1598; Found 685.1594 (red).

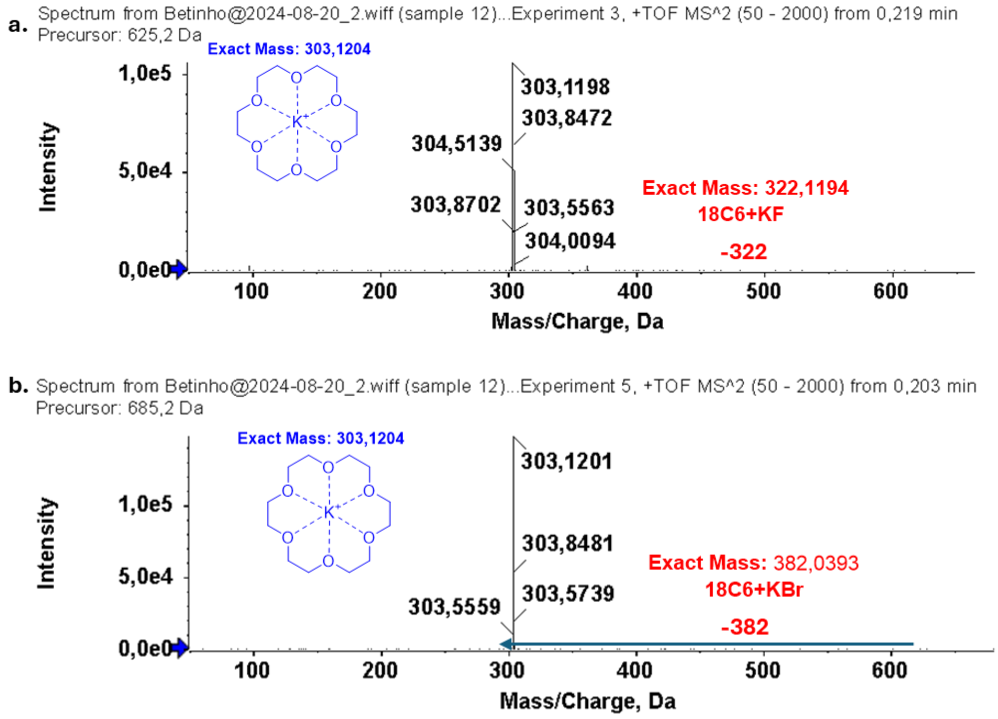

Figure S20. ESI(+)-MS/MS of the signals of *m/z* 625 (a) and *m/z* 685 (b)

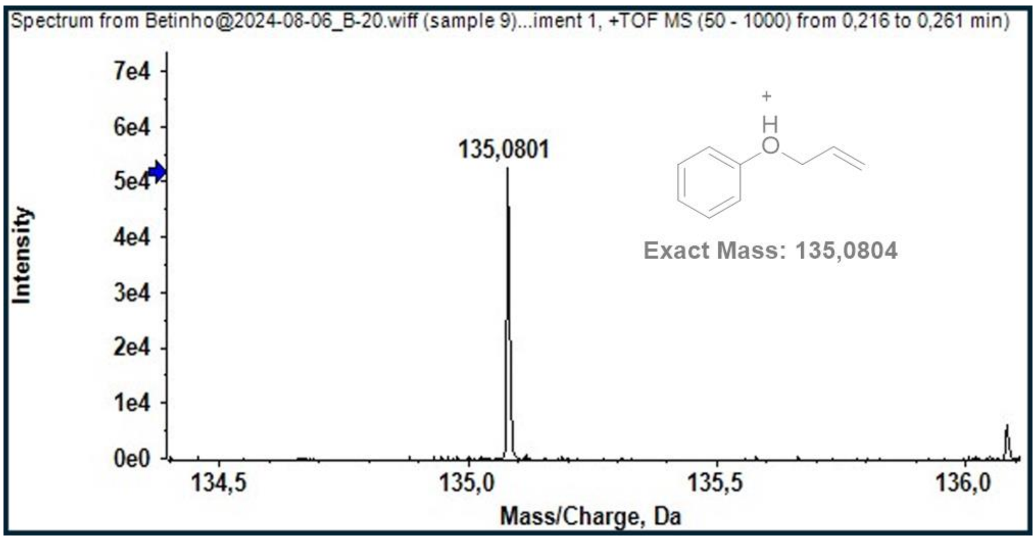

Figure S21. HRMS (ESI) m/z: [M+H]+ Calcd for $C_9H_{11}O^+$ 135.0804; Found 135.0801 spectrum expanded spectrum of **3**

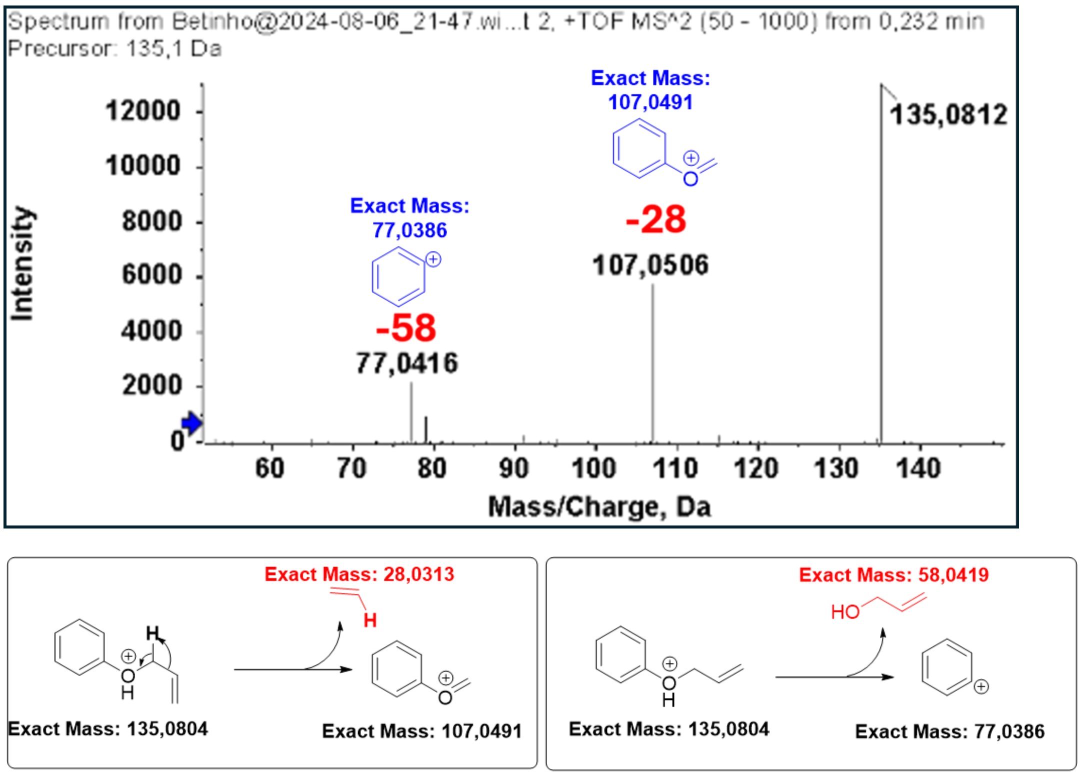

Figure S22. ESI(+)-MS/MS of the signal of *m/z* 135 and its respective fragmentation reactions (**3**)

1.4. NMR and HRMS spectra of the isolated compounds

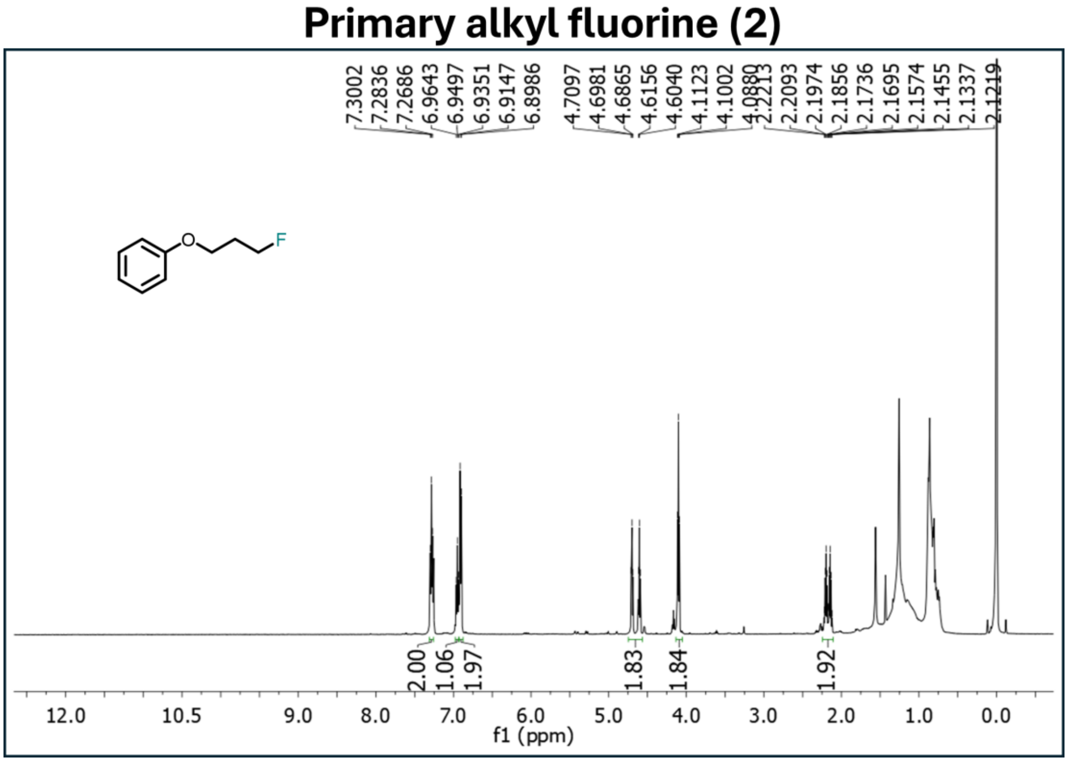

Figure S23. ^{1}H ($CDCl_3$, 500 MHz) spectrum of primary alkyl fluorine **2**

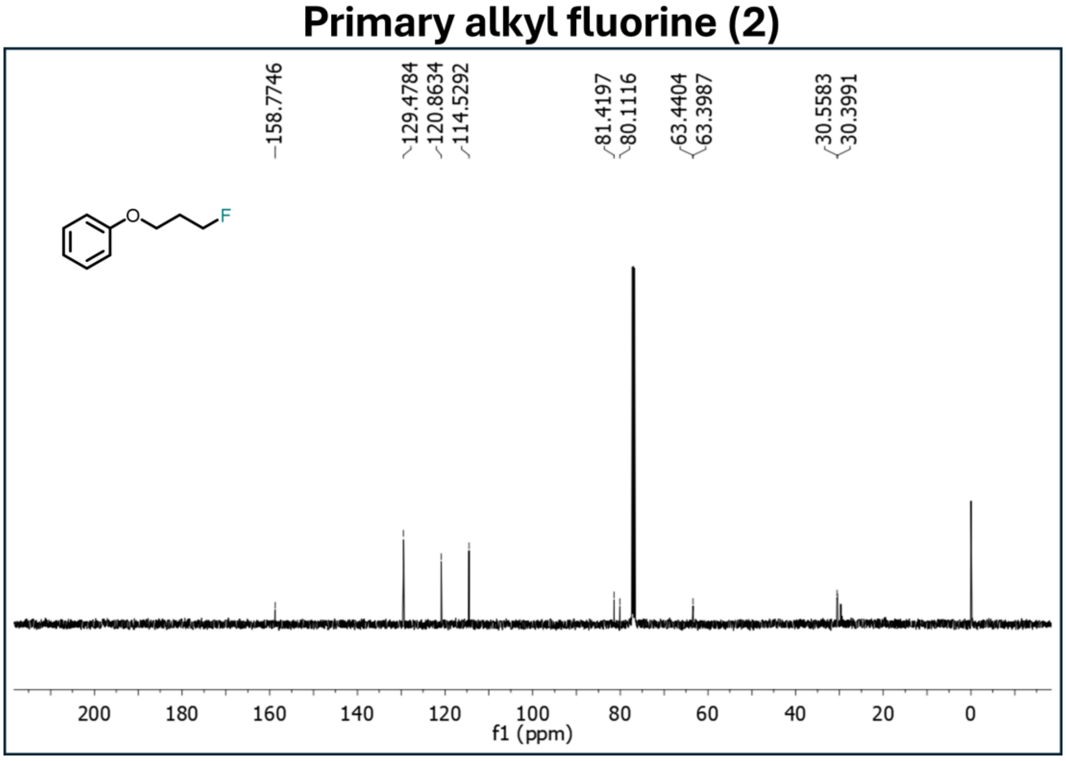

Figure S24. ^{13}C{^{1}H} NMR ($CDCl_3$, 125 MHz) spectrum of primary alkyl fluorine **2**

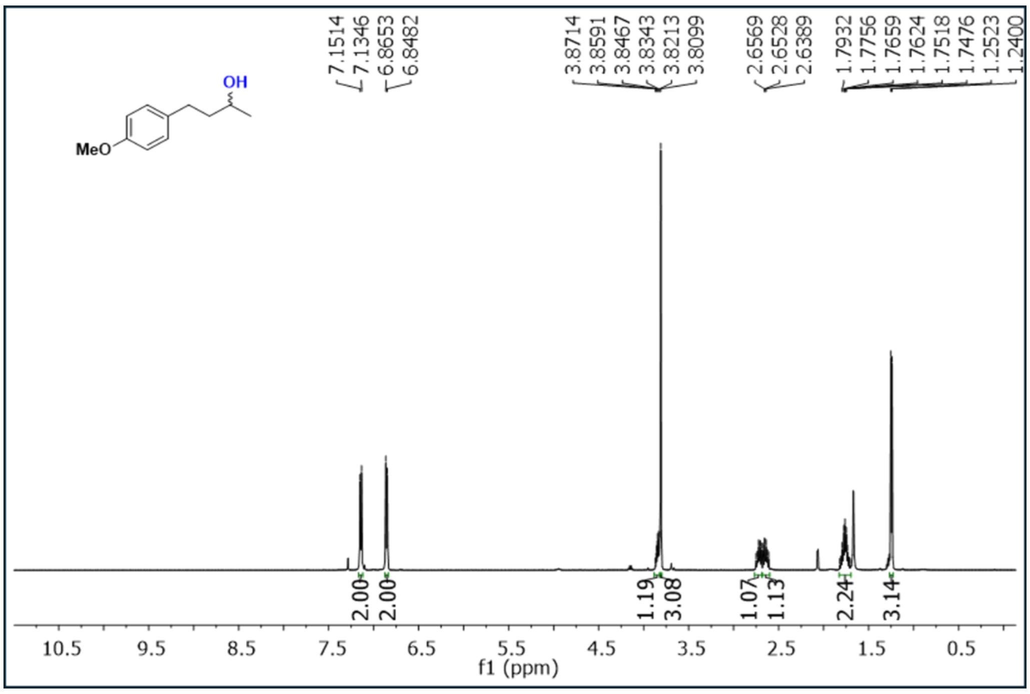

Figure S25. ^{1}H NMR ($CDCl_3$, 500 MHz) spectrum of the intermediate (secondary alcohol).

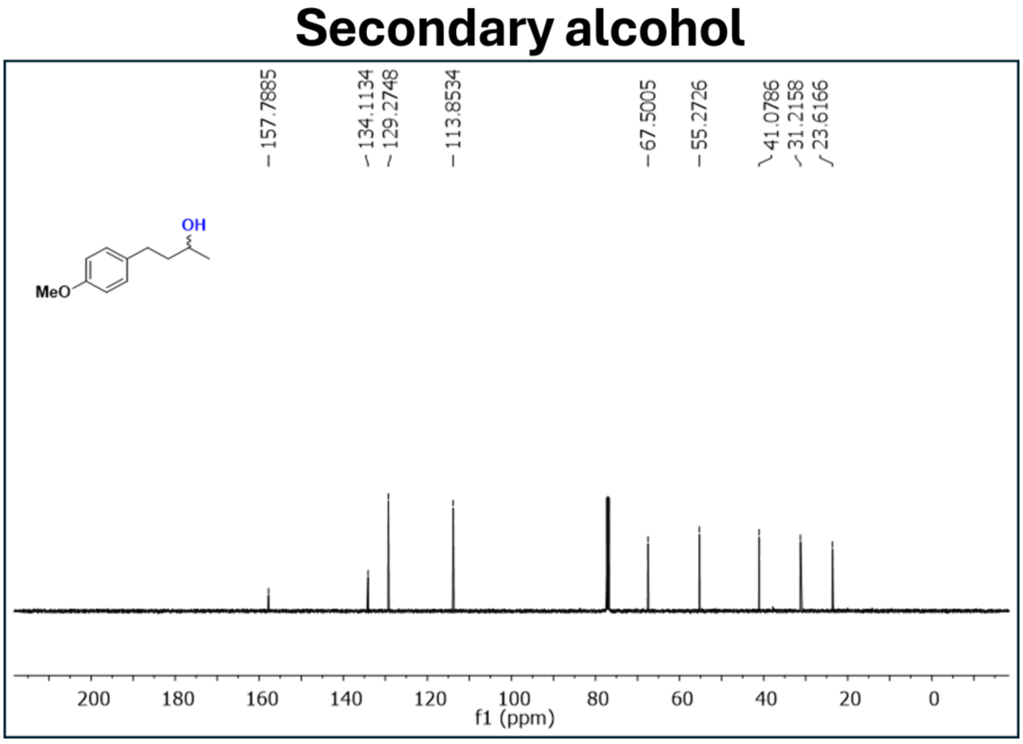

Figure S26. $^{13}C\{^{1}H\}$ NMR ($CDCl_3$, 125 MHz) spectra of the intermediate (secondary alcohol)

Secondary alkyl bromide (Compound 7)

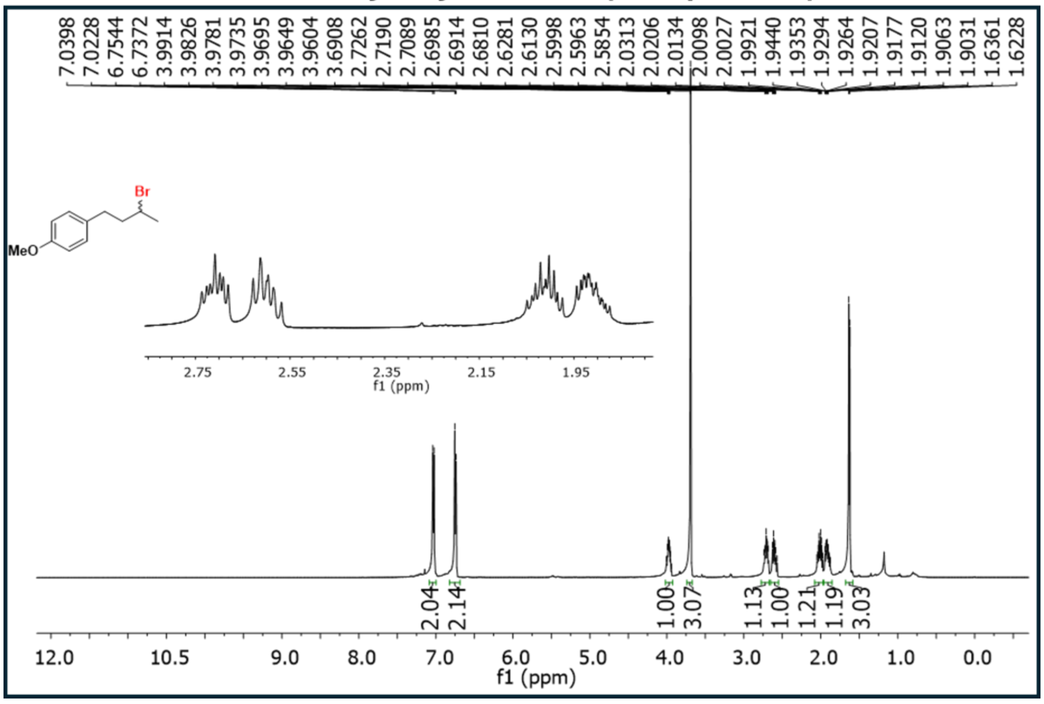

Figure S27. ^{1}H NMR ($CDCl_3$, 500 MHz) spectrum of **7**

Secondary alkyl bromide (Compound 7)

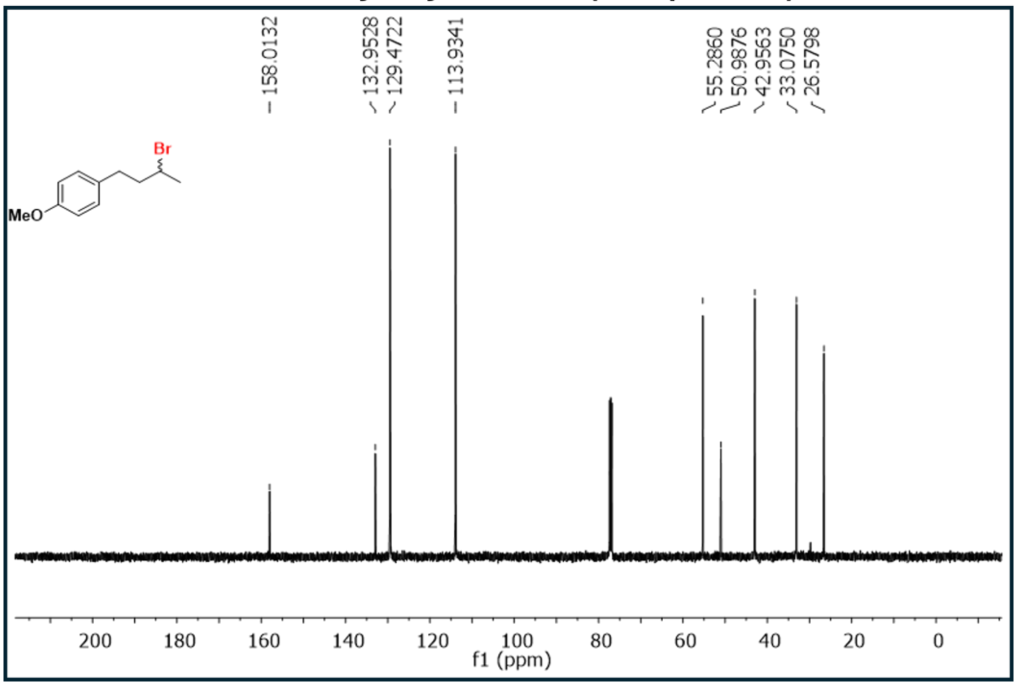

Figure S28. $^{13}C\{^{1}H\}$ NMR ($CDCl_3$, 125 MHz) spectrum of **7**

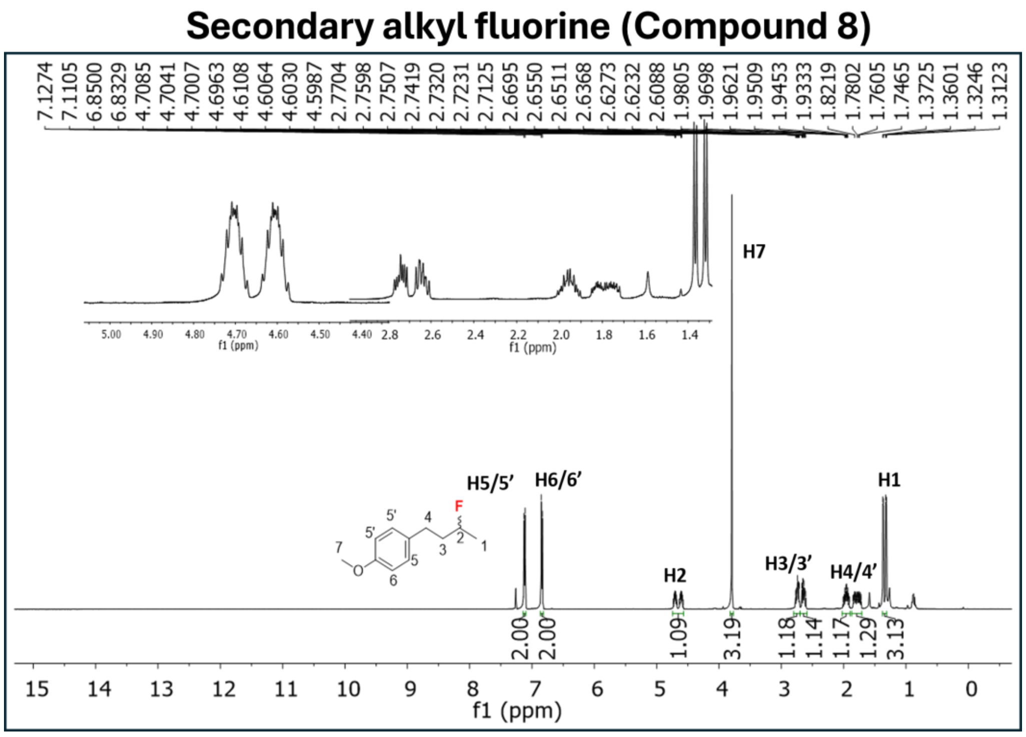

Figure S29. ^{1}H NMR ($CDCl_3$, 500 MHz) spectrum of **8**

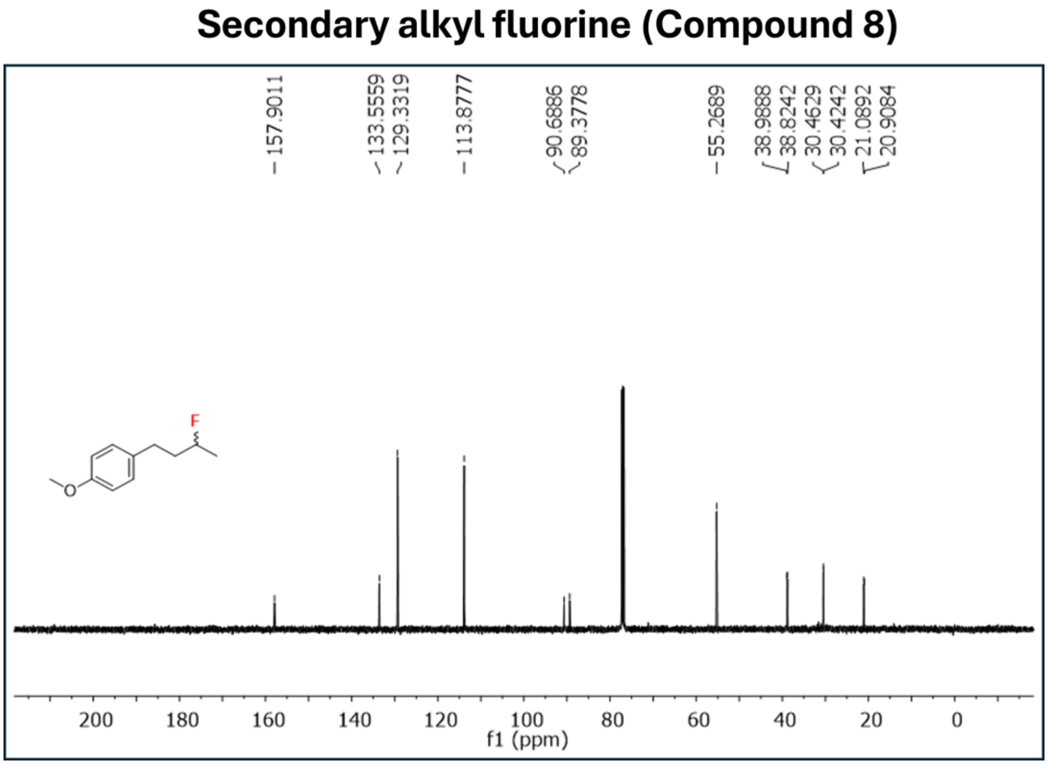

Figure S30. $^{13}C\{^{1}H\}$ NMR ($CDCl_3$, 125 MHz) spectrum of **8**

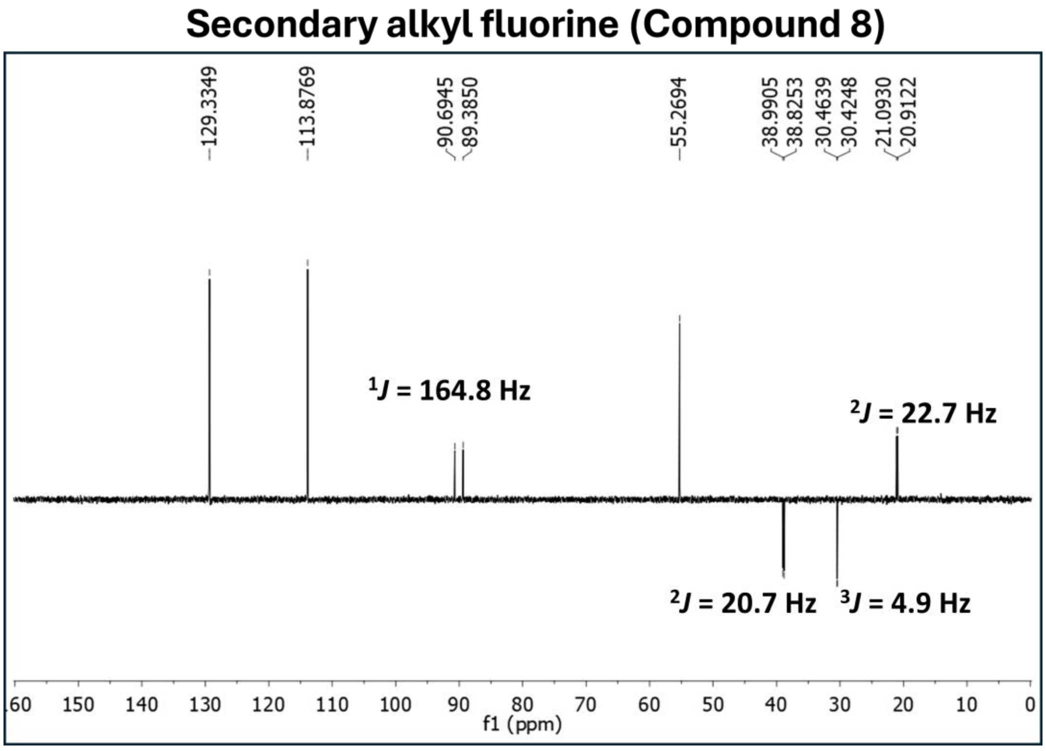

Figure S31. DEPT135 of **8**

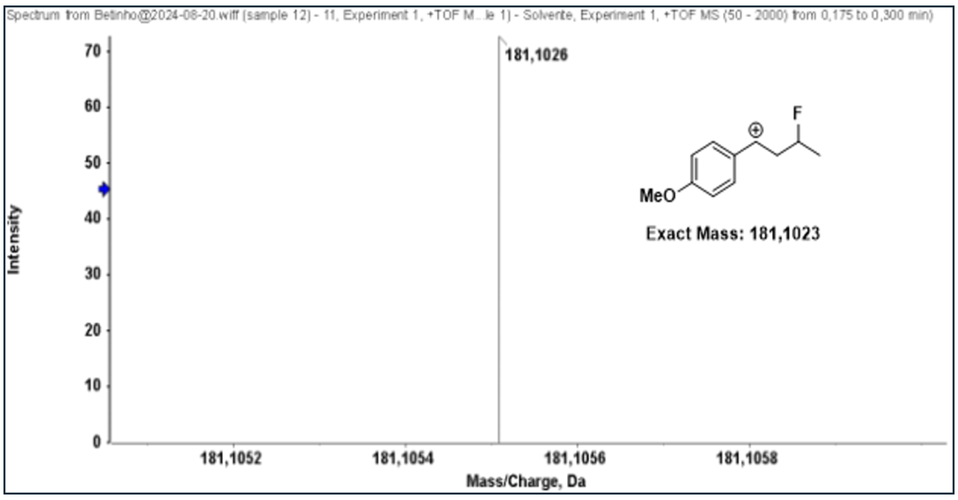

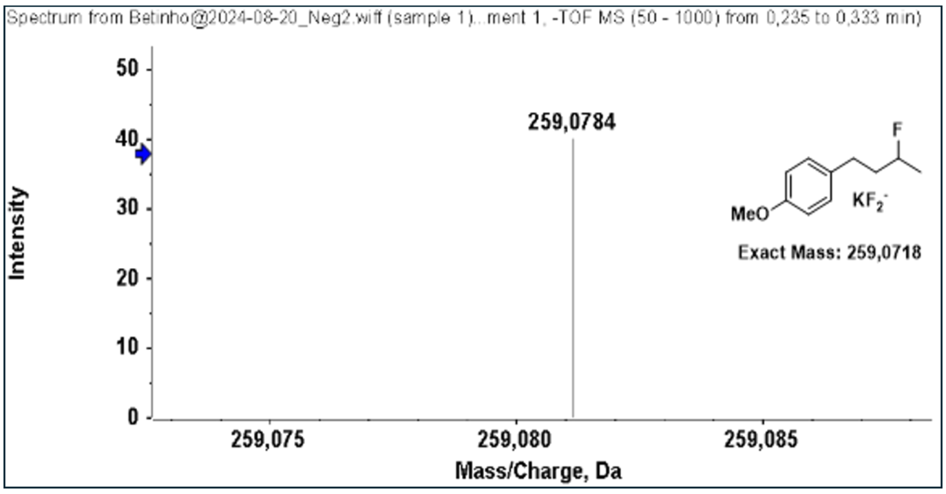

Figure S32. HRMS (ESI) m/z: $[M]^+$ Calcd for $C_{11}H_{14}FO^+$ 181.1023; Found 181.1026 (a) and HRMS (ESI) m/z: $[M+KF_2]^-$ Calcd for $C_{11}H_{15}F_3KO^-$ 259.0718; Found 259.0784 (b) expanded spectra of Compound **8**

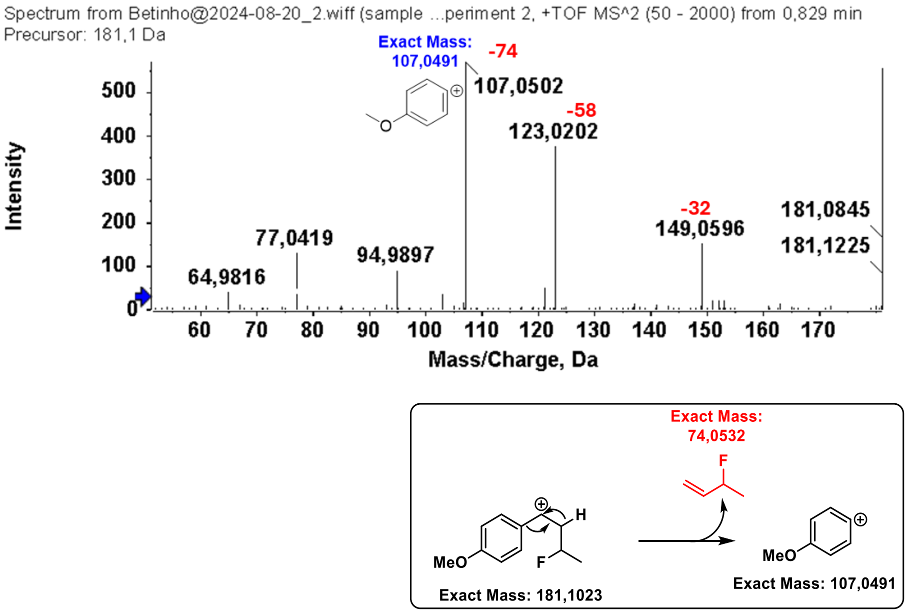

Figure S33. ESI(+)-MS/MS of the signal of *m/z* 181 and its respective fragmentation reaction

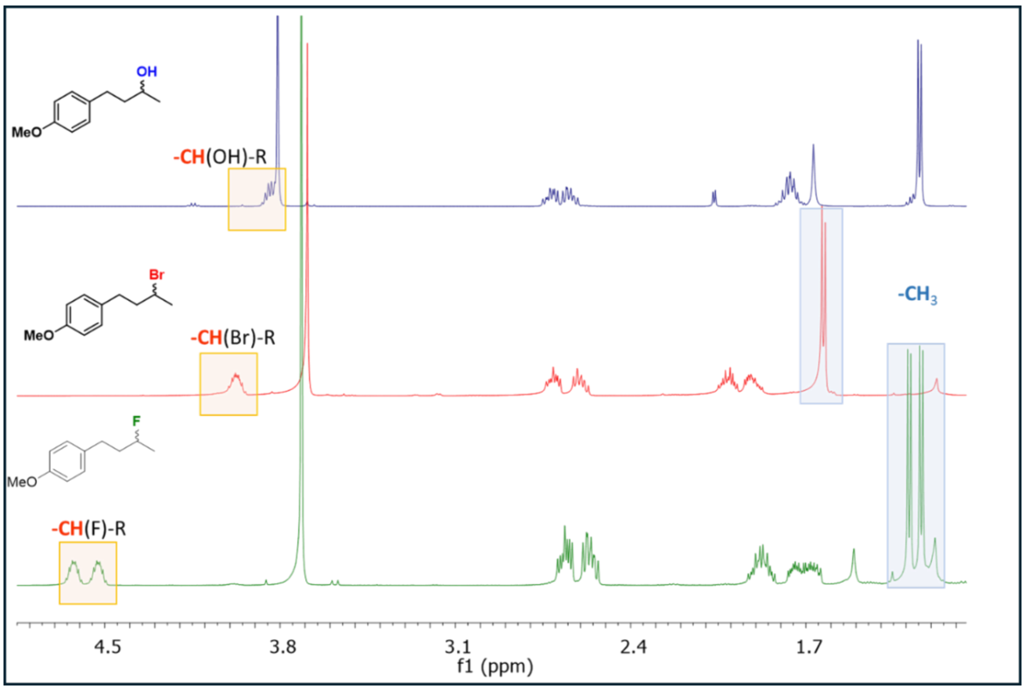

Figure S34.^{1}H NMR spectra of ketone reduction, bromination, and fluorination reactions

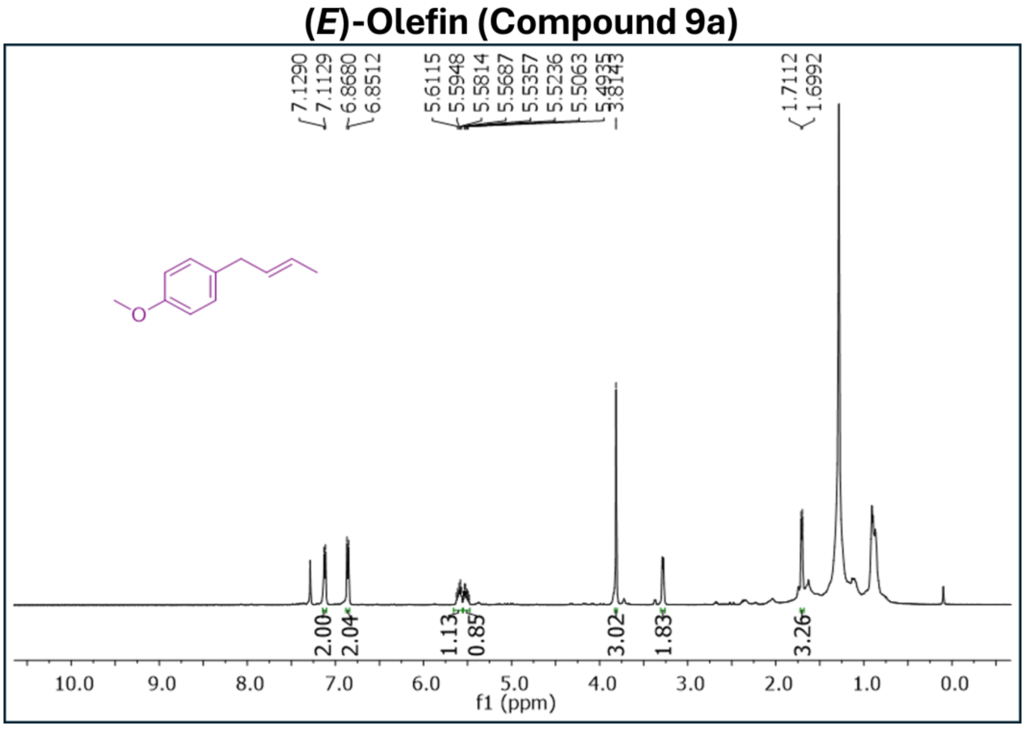

Figure S35. ^{1}H NMR ($CDCl_3$, 500 MHz) spectrum of **9a**

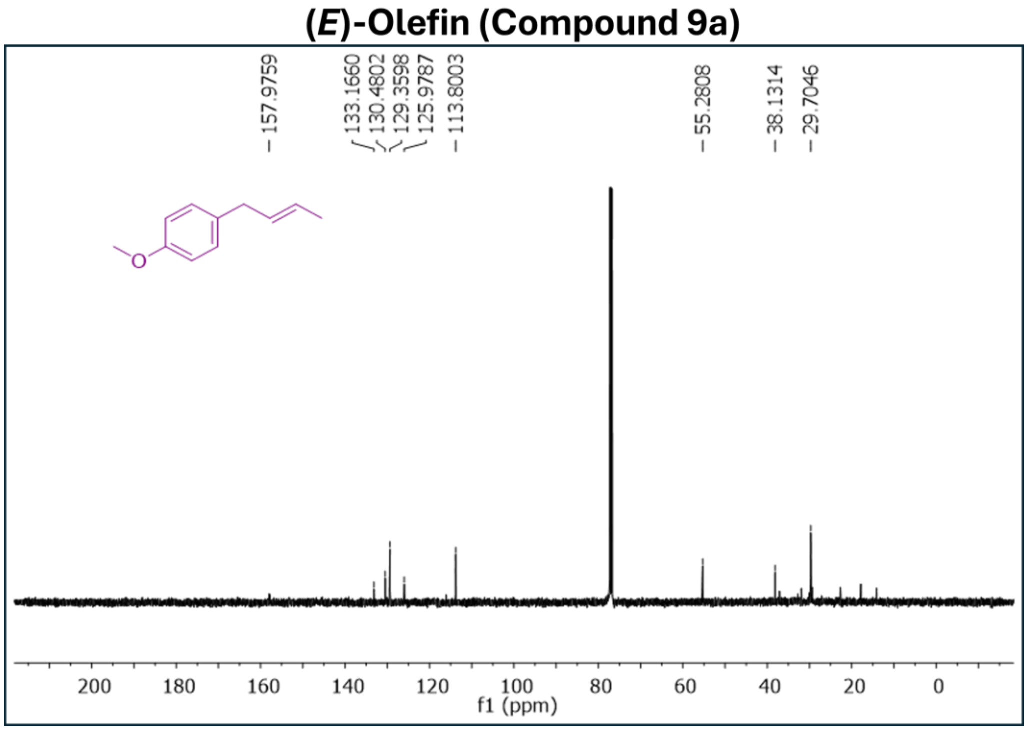

Figure S36. $^{13}C\{^{1}H\}$ NMR ($CDCl_3$, 125 MHz) spectrum of **9a**

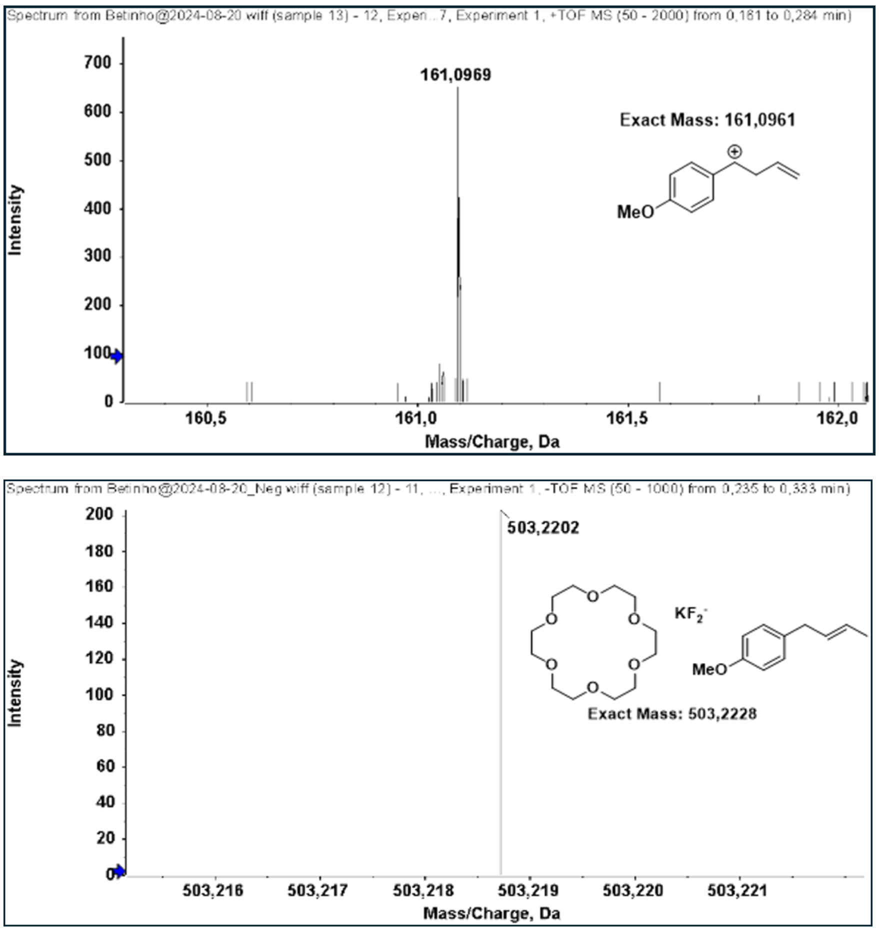

Figure S37. HRMS (ESI) m/z: of the $[M]^+$ Calcd for C11H13O+ Calcd for 161.0961; Found 161.0969; and HRMS (ESI) m/z: $[M+18C6+KF_2]^-$ $C_{23}H_{38}F_2KO_7^-$

Calcd for 503.2228; Found 503.2202 expanded spectra of **9**

Isolated olefin moieties (9)

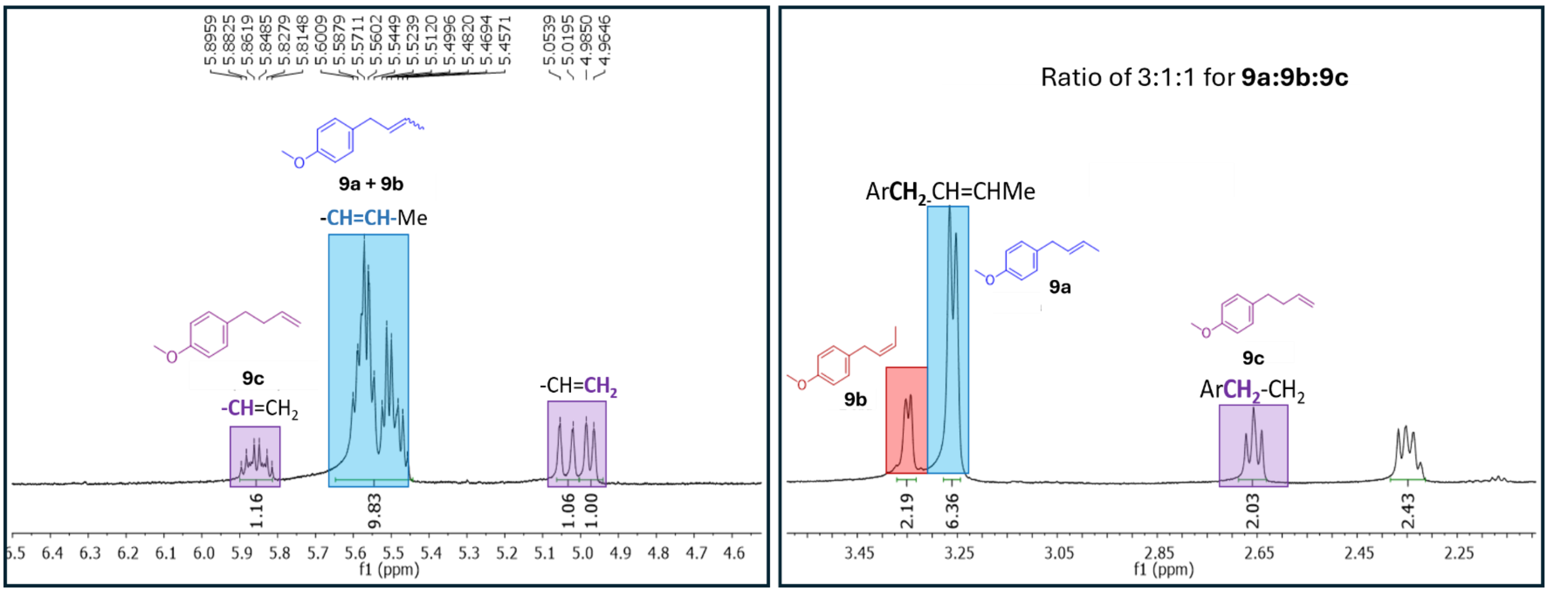

Comment: The ratio of E2 products was measured after isolating the olefins from the reaction mixture. However, neither (*Z*)-olefin nor the monosubstituted one could not be isolated.

Figure S38. ^{1}H NMR spectra of isolated olefin products

2. Control experiments: background reactions of C-O bond formation under basic media - hydrolysis and etherification reactions

The control experiments were performed in the presence of enough strong bases to generate the respective alkoxides. After isolating and characterizing the ethers, we proceeded with the analysis of the basicity profile of the fluoride anion.

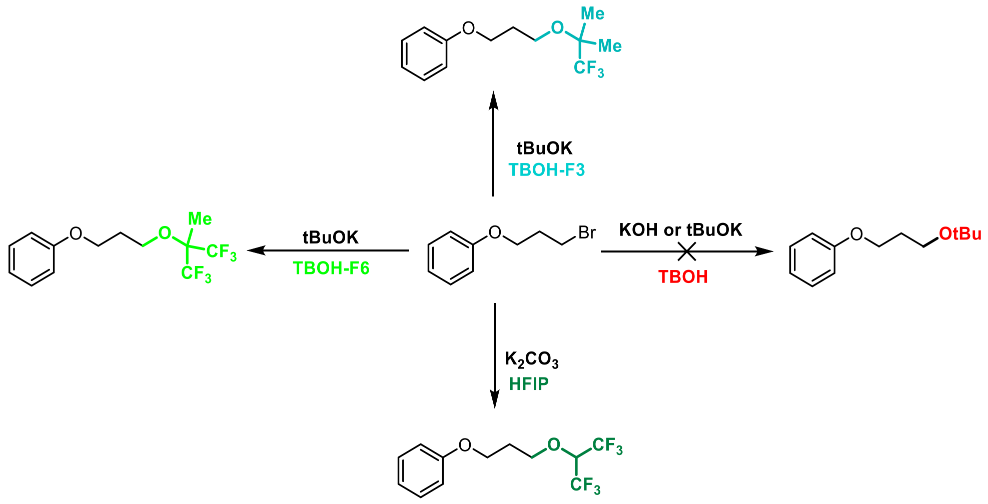

Scheme S4. General scheme of ether formation reactions

2.1. Experimental procedures

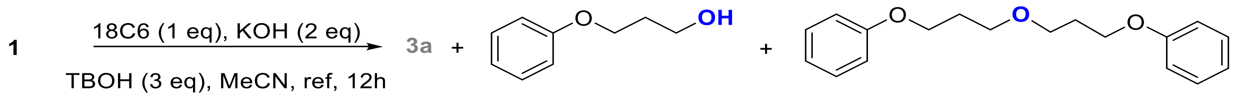

Alcohol (4) Dimer (6)

Scheme S5. Control experiment 1: Hydrolysis reaction to afford alcohol **4** dimer **6**

Hydrolysis reaction (Control experiment 1): In a round-bottomed flask, coupled to a condenser, the primary alkyl bromide (0.1076 g, 0.5 mmol), 18C6 (0.2640 g, 1.0 mmol), KOH (0.056 g, 1 mmol) and TBOH (0.111 g, 0.15 mL, 1.5 mmol) were added to 2 mL of acetonitrile. The reaction was stirred under reflux for 12 hours and the solvent was removed under reduced pressure. The yields were measured only by ^{1}H NMR analysis. The isolated yields were not determined due to difficult complete separation, which would lead to reduced yield values. The pure spectrum of **4** and **6** was obtained after column chromatography of the crude reaction, solvent hexane:AcOEt from 99:1 to 95: 5.

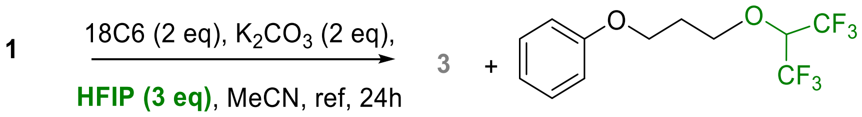

HFIP-ether (5a)

Scheme S6. Control experiment 2: Etherification reaction to afford the ether **5a**

HFIP-ether reaction (Control experiment 2): In a round-bottomed flask, coupled to a condenser, 18C6 (0.2640 g, 1.0 mmol), K_2CO_3 (0.056 g, 1.0 mmol) and HFIP (0.252 g, 0.15 mL, 1.5 mmol) were

added to 1 mL of acetonitrile. After, a solution of 1 mL containing the primary alkyl bromide (0.1076 g, 0.5 mmol), was added dropwise. The reaction was stirred under reflux for 12 hours and the solvent was removed under reduced pressure. The yield was measured only by ^{1}H NMR analysis. The isolated yield was not determined due to difficult complete separation, which would lead to reduced yield values. The pure spectrum of **5a** was obtained after column chromatography of the crude reaction, solvent hexane:AcOEt from 99:1 to 90:10.

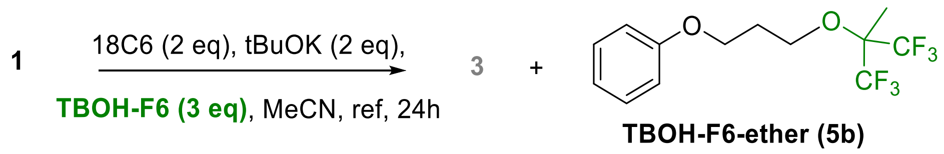

Scheme S7. Control experiment 3: Etherification reaction to afford the ether **5b**

TBOH-F6-ether reaction (Control experiment 3): In a round-bottomed flask, coupled to a condenser, 18C6 (0.2640 g, 1.0 mmol), tBuOK (0.112 g, 1.0 mmol) and TBOH-F6 (0.2730 g, 0.18 mL, 1.5 mmol) were added to 1 mL of acetonitrile. After, a solution of 1 mL containing the primary alkyl bromide (0.1076 g, 0.5 mmol), was added dropwise. The reaction was stirred under reflux for 12 hours and the solvent was removed under reduced pressure. The yield was measured only by ^{1}H NMR analysis. The isolated yield was not determined due to difficult complete separation, which would lead to reduced yield values. The pure spectrum of **5b** was obtained after column chromatography of the crude reaction, solvent hexane:AcOEt from 99:1 to 90:10.

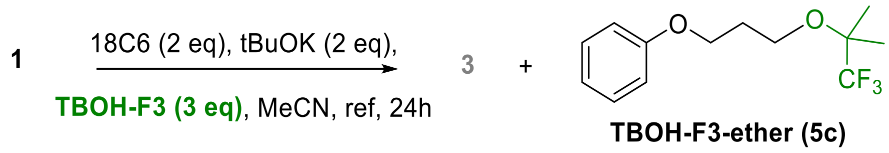

Scheme S8. Control experiment 4: Etherification reaction to afford the ether **5c**

TBOH-F3-ether reaction (Control experiment 4): In a round-bottomed flask, coupled to a condenser, 18C6 (0.1584 g, 0.6 mmol), tBuOK (0.0.067 g, 0.6 mmol) and TBOH-F3 (0.1152 g, 0.09 mL, 0.9 mmol) were added to 1 mL of acetonitrile. After, a solution of 1 mL containing the primary alkyl bromide (0.0717 g, 0.3 mmol), was added dropwise. The reaction was stirred under reflux for 12 hours and the solvent was removed under reduced pressure. The yield was measured only by ^{1}H NMR analysis. The isolated yield was not determined due to difficult complete separation, which would lead to reduced yield values. The pure spectrum of **5c** was obtained after column chromatography of the crude reaction, solvent hexane:AcOEt from 99:1 to 90:10.

2.2. Characterization data of isolated alcohol, alkyl and aryl ether derivatives

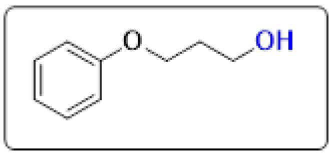

3-phenoxypropan-1-ol (4): The product **4** was obtained as a colorless oil. ^{1}H NMR (500 MHz, $CDCl_3$) δ 7.32 – 7.28 (m, 1H), 6.95 (t, *J* = 7.5 Hz, 1H), 6.91 (d, *J* = 7.5 Hz, 2H), 4.14 (t, *J* = 5.9 Hz, 2H), 3.88 (t, *J* = 5.8 Hz, 2H), 2.06 (qui, *J* = 6.0 Hz, 2H), 1.57 (s, 1H). ^{13}C{1H} NMR (125 MHz, $CDCl_3$) δ 149.8, 129.5, 120.9, 114.5, 65.8, 60.6, 32.0. HRMS (ESI) *m/z*: $[M+H]^+$ Calcd for $C_9H_{13}O_2^+$ 153.0910; Found 153.0905; $[M+Na]^+$ Calcd for $C_9H_{12}O_2Na^+$ 175.0730; Found 175.0736.

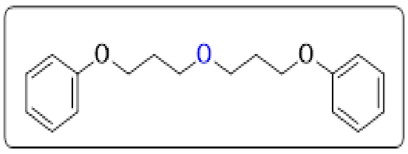

((oxybis(propane-3,1-diyl))bis(oxy))dibenzene (6): The product **6** was obtained as a colorless oil. ^{1}H NMR (500 MHz, $CDCl_3$) δ 7.31 – 7.26 (m, 2H), 6.95 (t, *J* = 7.3 Hz, 1H), 6.90 (d, *J* = 8.3 Hz, 2H), 4.07 (t, *J* = 6.2 Hz, 2H), 3.65 (t, *J* = 6.1 Hz, 2H), 2.07 (qui, *J* = 6.2 Hz, 2H). ^{13}C{1H} NMR (125 MHz, $CDCl_3$) δ 159.0, 129.4, 120.6, 114.6, 67.4, 64.8, 29.7. HRMS (ESI) *m/z*: $[M]^+$ Calcd for $C_{18}H_{22}NaO_3^+$ 309.146; Found 309.1464.

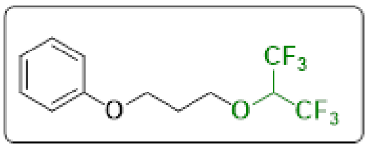

(3-((1,1,1,3,3,3-hexafluoropropan-2-yl)oxy)propoxy)benzene (5a): The product **5a** was obtained as a colorless oil. ^{1}H NMR (500 MHz, $CDCl_3$) δ 7.21 (t, *J* = 7.6 Hz, 2H), 6.88 (t, *J* = 7.6 Hz, 1H), 6.82 (d, *J* = 7.6 Hz, 2H), 4.00-3.94 (m, 5H), 2.05 (qui, *J* = 6.0 Hz, 2H). ^{13}C{1H} NMR (125 MHz, $CDCl_3$) δ 158.7, 129.5, 121.6 (q, ^1J =284 Hz, CF_3), 120.9, 114.5, 76.7 (hept, ^2J = 34 Hz, -$C(CF_3)_2$), 72.1, 63.5, 29.6. HRMS (ESI) *m/z*: $[M]^+$ Calcd for $C_{12}H_{11}F_6O_2^+$ 301.0658; Found 301.0674.

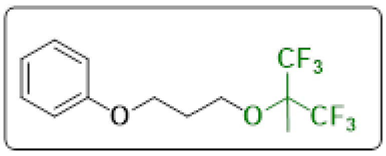

(3-((1,1,1,3,3,3-hexafluoro-2-methylpropan-2-yl)oxy)propoxy)benzene (5b): The product **5b** was obtained as a colorless oil. ^{1}H NMR (500 MHz, $CDCl_3$) δ 7.33 (t, *J* = 7.6 Hz, 2H), 7.00 (t, *J* = 7.6 Hz, 1H), 6.95 (d, *J* = 7.6Hz, 2H), 4.11 (t, *J* = 6.0 Hz, 2H), 3.94 (t, *J* = 5.9 Hz, 2H), 2.13 (dqui, *J* = 5.9 Hz, 2H), 1.63 (s, 3H). ^{13}C{1H} NMR (125 MHz, $CDCl_3$) δ 158.8, 129.5, 123.1 (q, ^1J = 288 Hz, CF_3), 120.8, 114.5, 78.1 (hept, ^2J = 29 Hz, -$C(CF_3)_2$), 63.7, 62.3, 29.7, 12.6. HRMS (ESI) *m/z*: $[M+H]^+$ Calcd for $C_{13}H_{15}F_6O_2^+$ 317.0971; Found 317.0992.

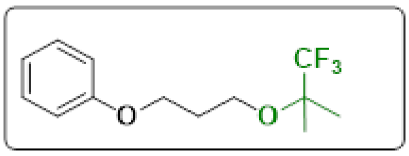

(3-((1,1,1-trifluoro-2-methylpropan-2-yl)oxy)propoxy)benzene (**5c**): The product **5b** was obtained as a colorless oil. ^{1}H NMR (500 MHz, $CDCl_3$) δ 7.31 (d, *J* = 7.6 Hz, 2H), 6.98 – 6.91 (m, 3H), 4.07 (t, *J* = 6.1 Hz, 2H), 3.72-3.71 (m, 3H), 2.08 – 2.01 (m, 2H), 1.65 (s, 6H). ^{13}C{1H} NMR (125 MHz, $CDCl_3$) δ 150.5, 147.6, 129.5, 124.8, 114.6, 59.6, 30.0, 20.3. HRMS (ESI) *m/z*: $[M+H]^+$ Calcd for $C_{13}H_{17}F_3NaO_2^+$ 285.1073; Found 285.1106.

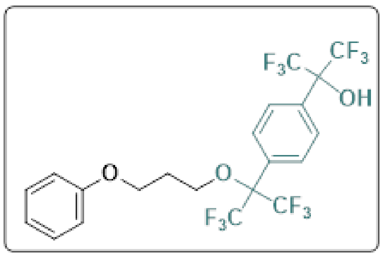

1,1,1,3,3,3-hexafluoro-2-(4-(1,1,1,3,3,3-hexafluoro-2-(3-phenoxypropoxy)propan-2-yl)phenyl)propan-2-ol (5e): The product **5e** was obtained as a colorless oil. ^{1}H NMR (500 MHz, $CDCl_3$) δ 7.72 (d, *J* = 8.2 Hz, 2H), 7.63 (d, *J* = 8.2 Hz, 2H), 7.34 (t, *J* = 7.7 Hz, 2H), 7.01 (t, *J* = 7.7 Hz, 1H), 6.93 (d, *J* = 7.7 Hz, 2H), 4.20 (t, *J* = 5.8 Hz, 2H), 3.83 (t, *J* = 5.7 Hz, 2H), 2.21 (qui, *J* = 5.7 Hz, 2H). ^{13}C{1H} NMR (125 MHz, $CDCl_3$) δ 158.6, 131.7, 130.4, 129.5, 128.4, 127.0, 120.9, 114.4, 70.4, 63.1, 29.6. HRMS (ESI) *m/z*: $[M+H]^+$ Calcd for $[C_{21}H_{17}F_{12}O_3]^+$ 545.0981; Found 545.0977, $[M+Na]^+$ Calcd for $[C_{21}H_{16}F_{12}NaO_3]^+$ 567.0800; Found 567.0795, $[M]^-$ Calcd for $C_{21}H_{15}F_{12}O_3^-$ 543.0835; Found 543.0870.

2.3. NMR and HRMS spectra of alcohol, alkyl and aryl ether derivatives

Hydrolysis product (4)

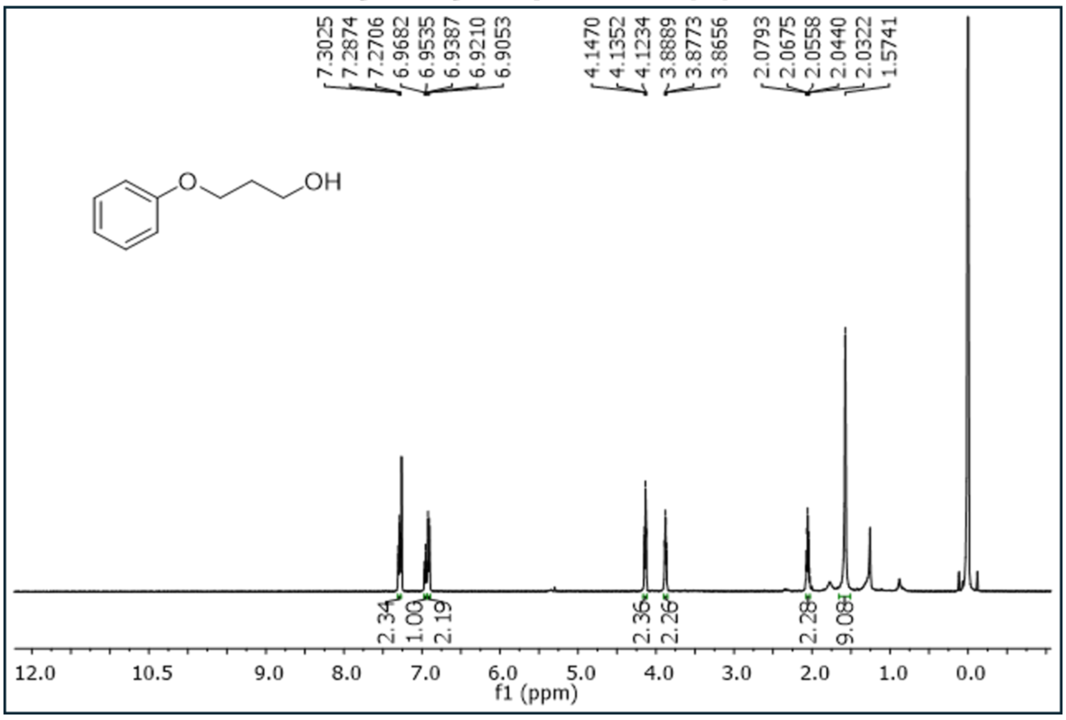

Figure S39. ^{1}H NMR ($CDCl_3$, 500 MHz) spectrum of **4**

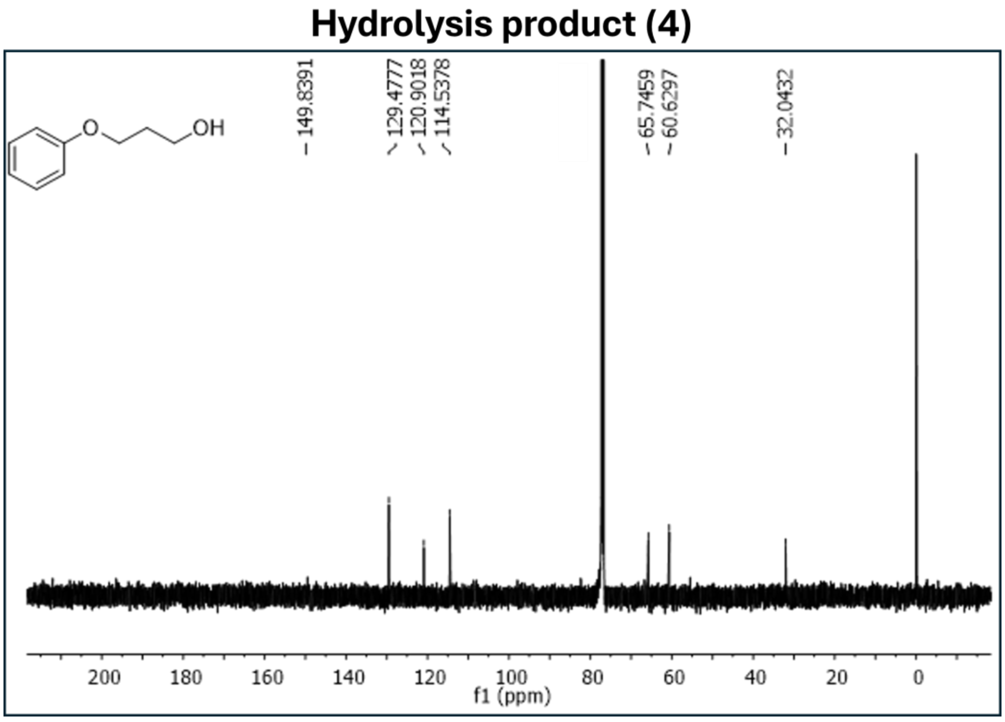

Figure S40. $^{13}C\{^{1}H\}$ NMR ($CDCl_3$, 125 MHz) spectrum of **4**

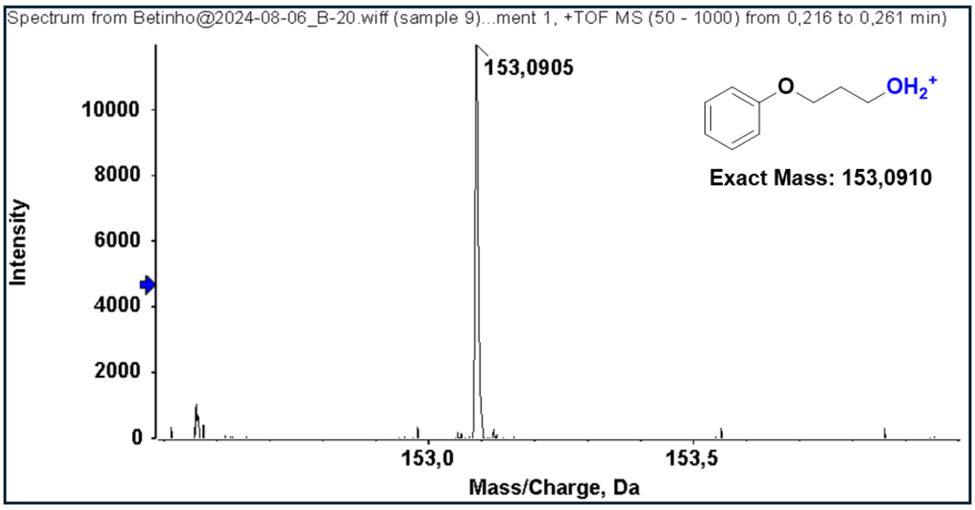

Figure S41. HRMS (ESI) m/z: [M + H]+ Calcd for $C_9H_{13}O_2^+$ 153.0910; Found 153.0905 expanded spectrum of **4**

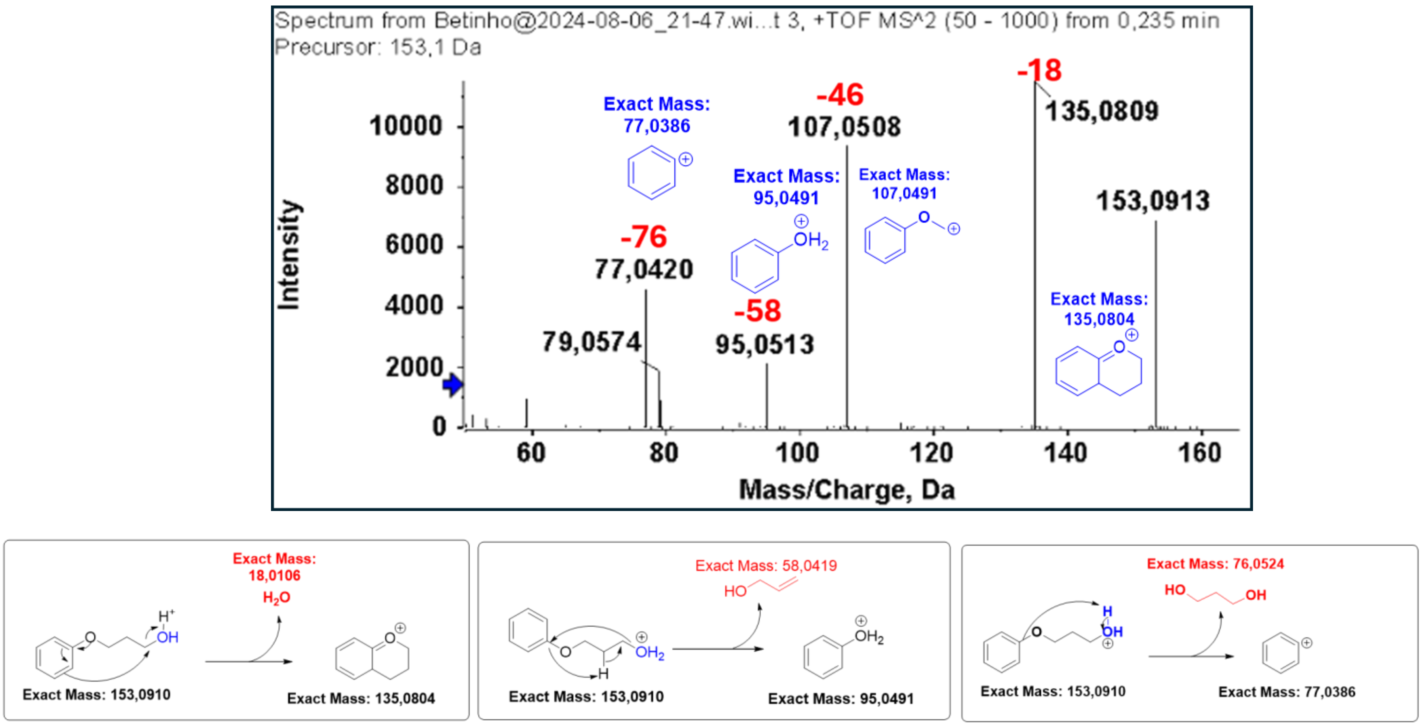

Figure S42. ESI(+)-MS/MS of the signal of *m/z* 153 and its respective fragmentation reactions

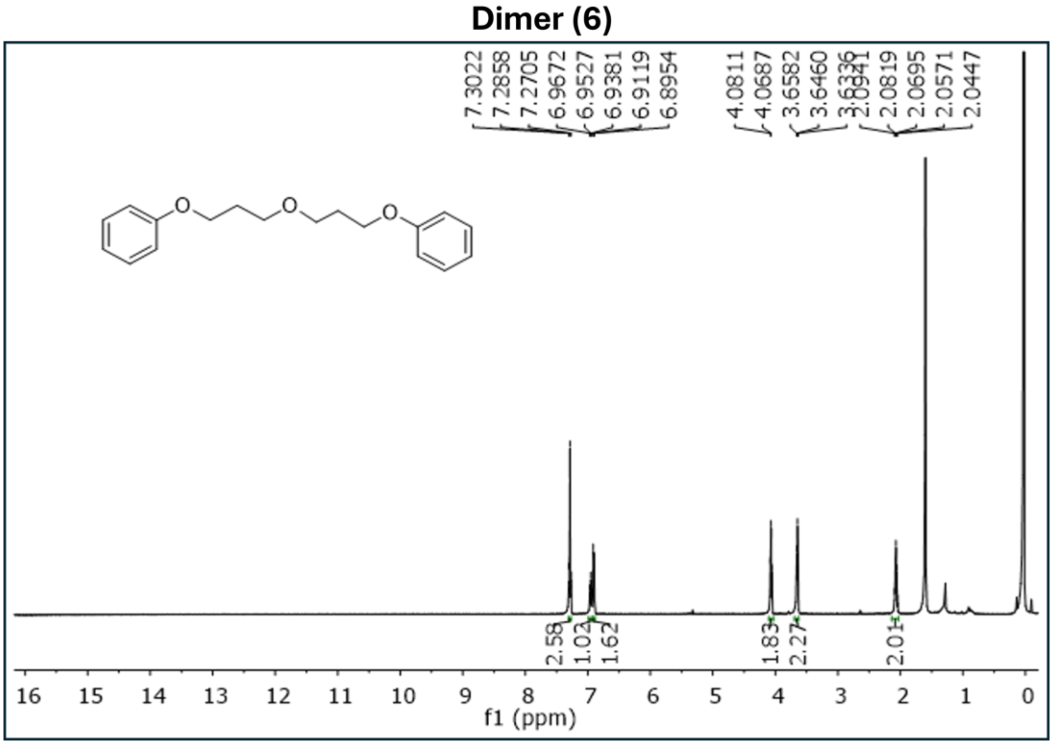

Figure S43. ^{1}H NMR ($CDCl_3$, 500 MHz) spectrum of **6**

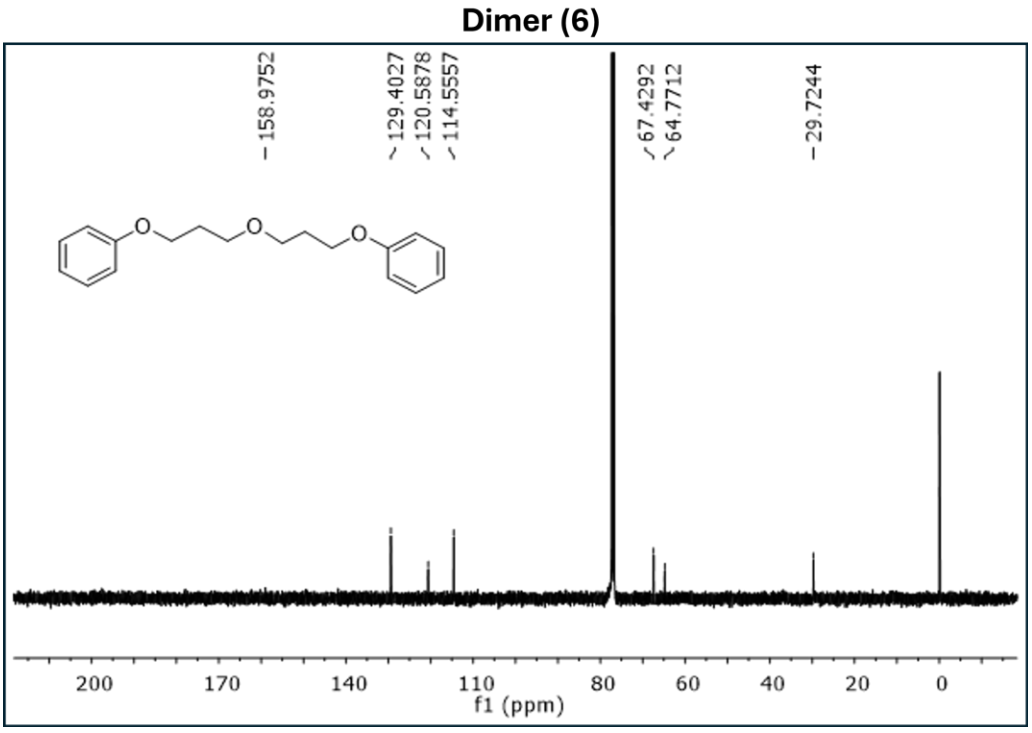

Figure S44. $^{13}C\{^{1}H\}$ NMR ($CDCl_3$, 125 MHz) spectrum of **6**

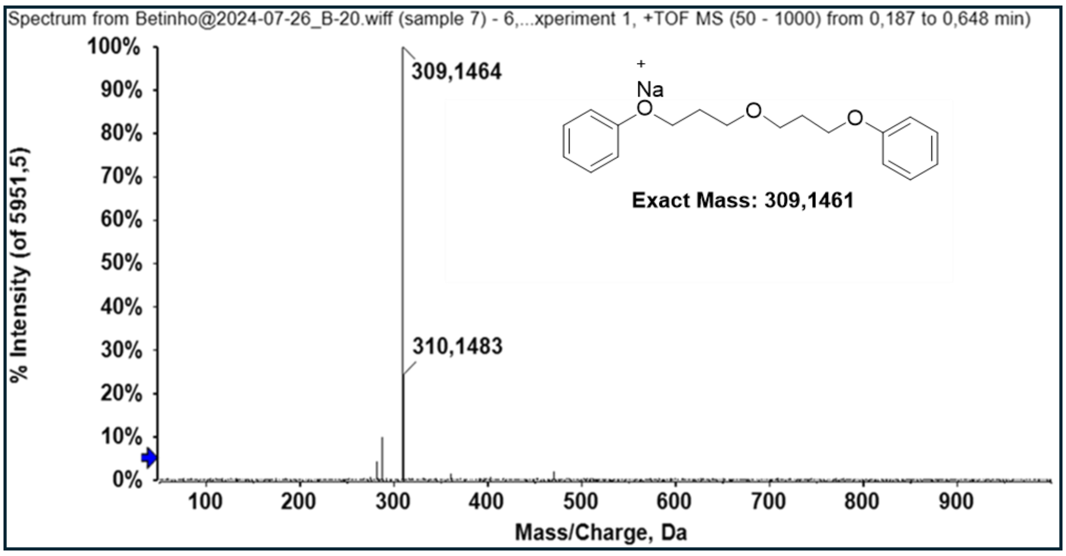

Figure S45. HRMS (ESI) m/z: [M + Na]+ Calcd for C18H22NaO3+ 309.1461; Found 309.1464 spectrum of **6**.

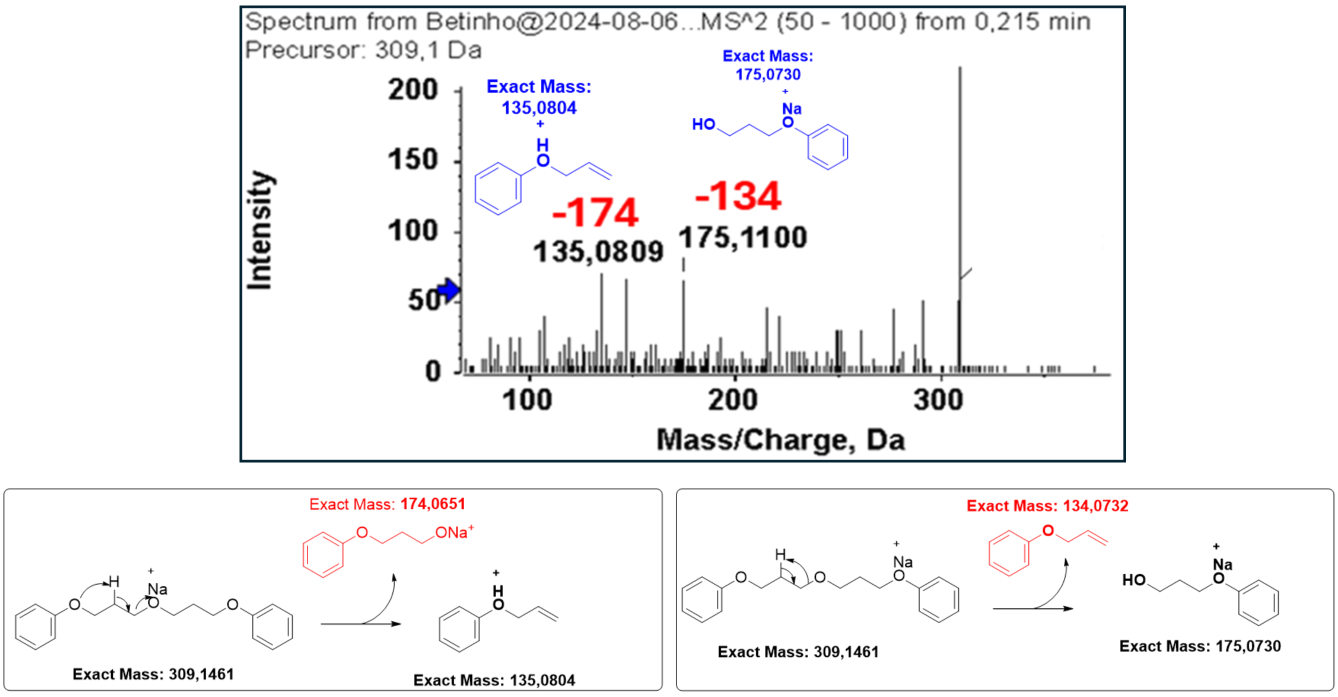

Figure S46. ESI(+)-MS/MS of the signal of *m/z* 309 and its respective fragmentation reactions

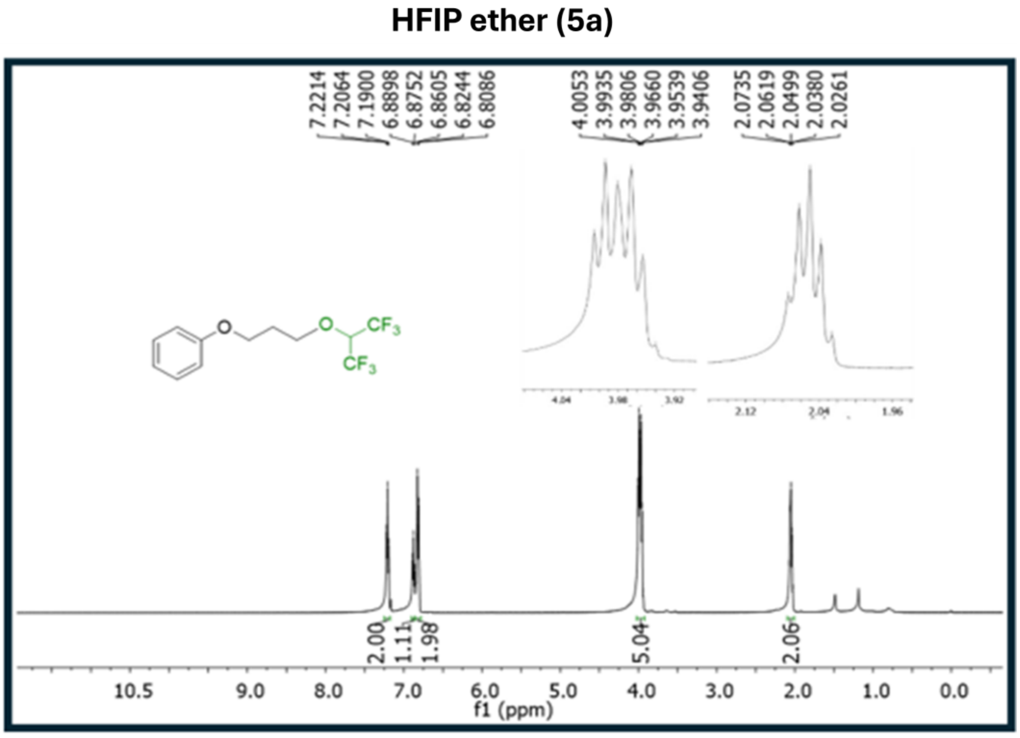

Figure S47. ^{1}H NMR ($CDCl_3$, 500 MHz) spectrum of **5a**

HFIP ether (5a)

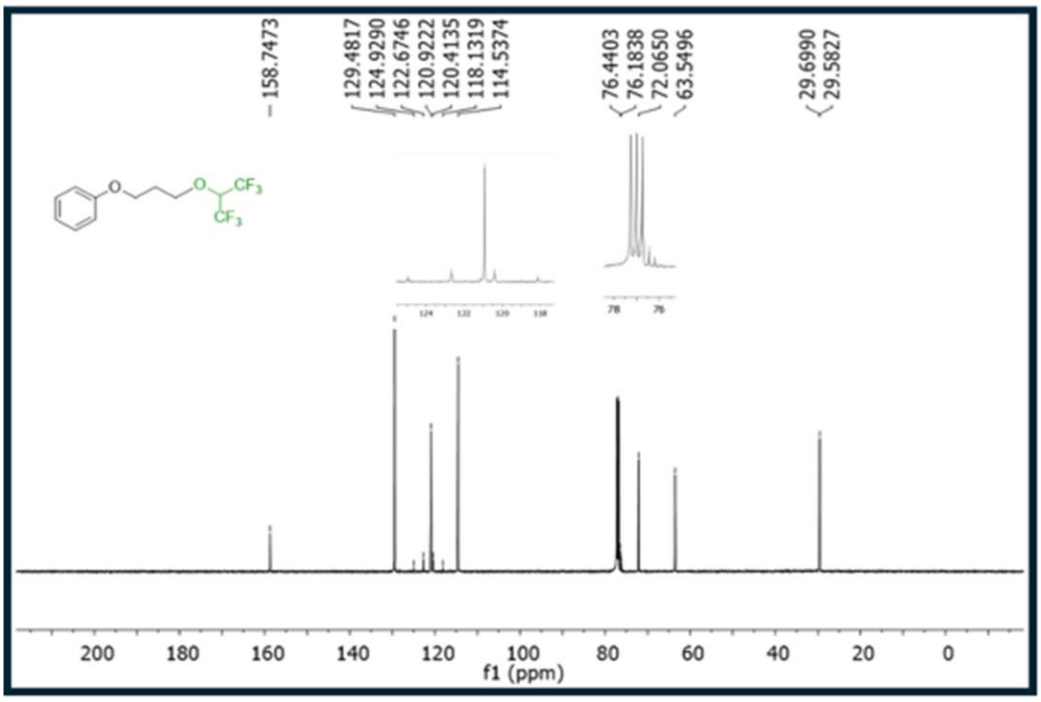

Figure S48. $^{13}C\{^1H\}$ NMR ($CDCl_3$, 125 MHz) spectrum of **5a**

HFIP ether (5a)

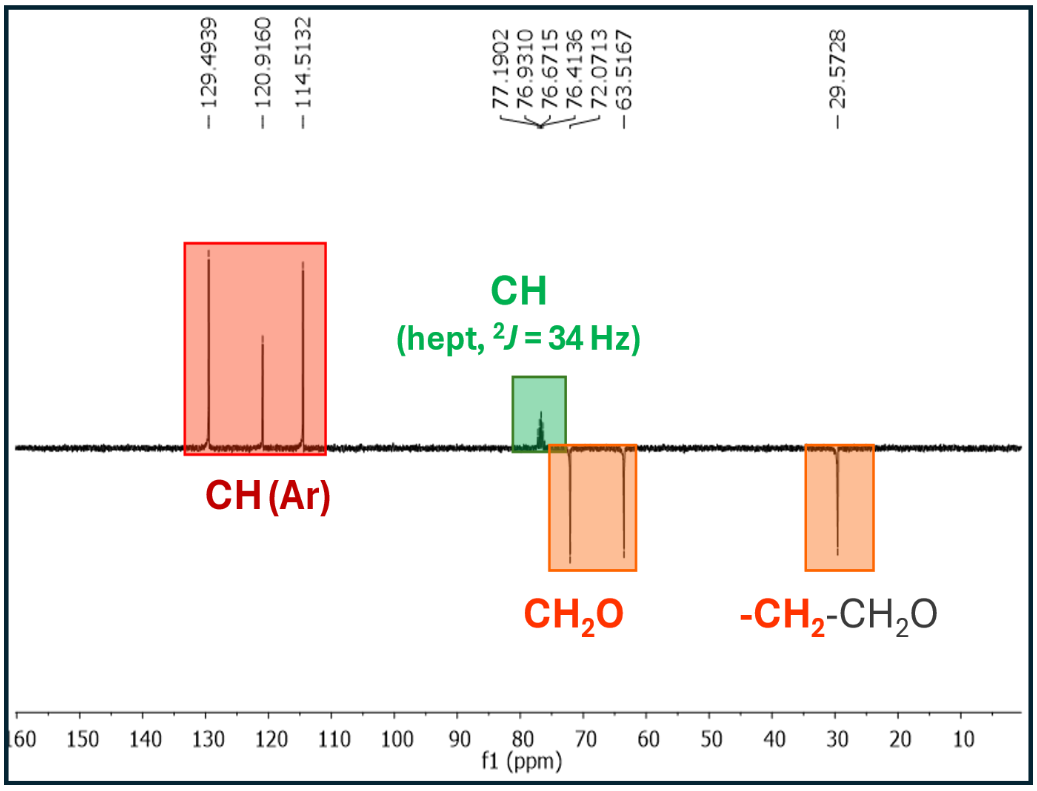

Figure S49. DEPT135 of **5a**

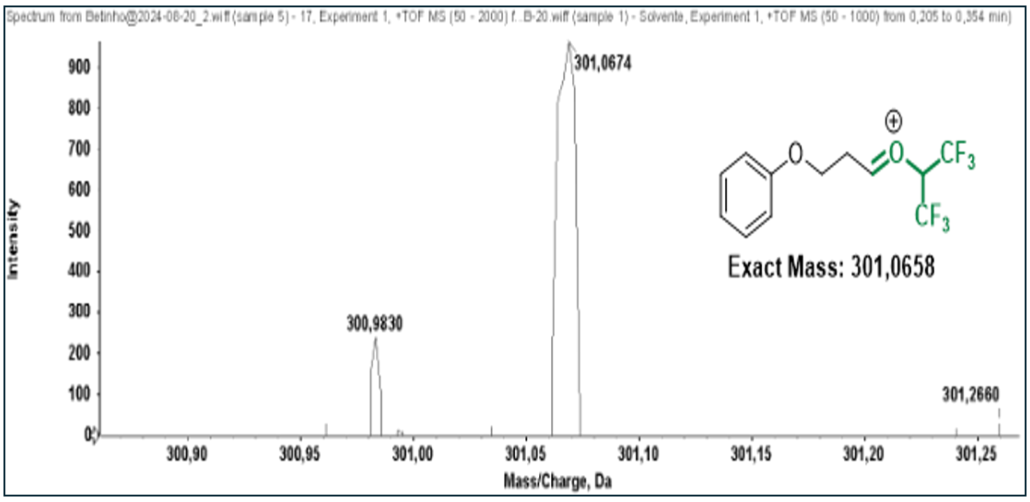

Figure S50. HRMS (ESI) *m/z*: $[M]^+$ Calcd for $C_{12}H_{11}F_6O_2^+$ 301.0674; Found 301.0658 expanded spectrum of **5a**

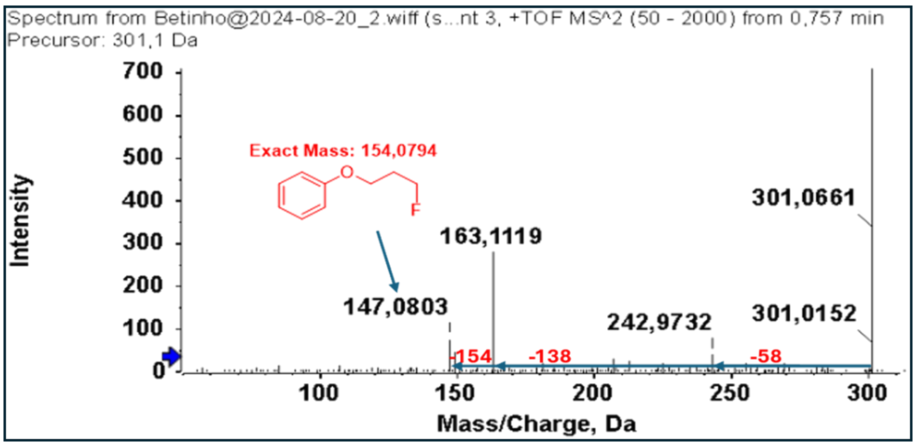

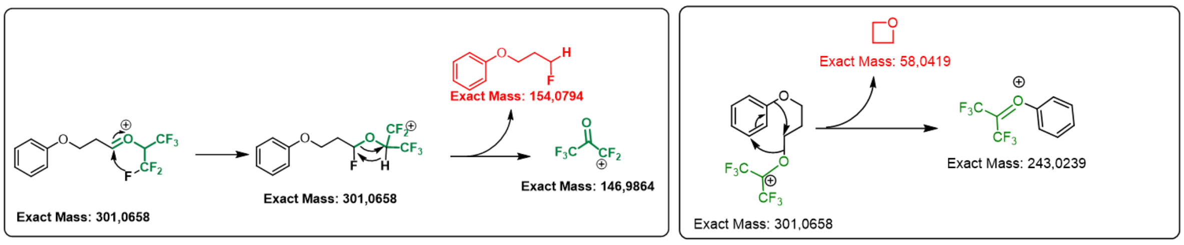

Figure S51. ESI(+)-MS/MS of the signal of *m/z* 301 and its respective fragmentation reactions (**5a**)

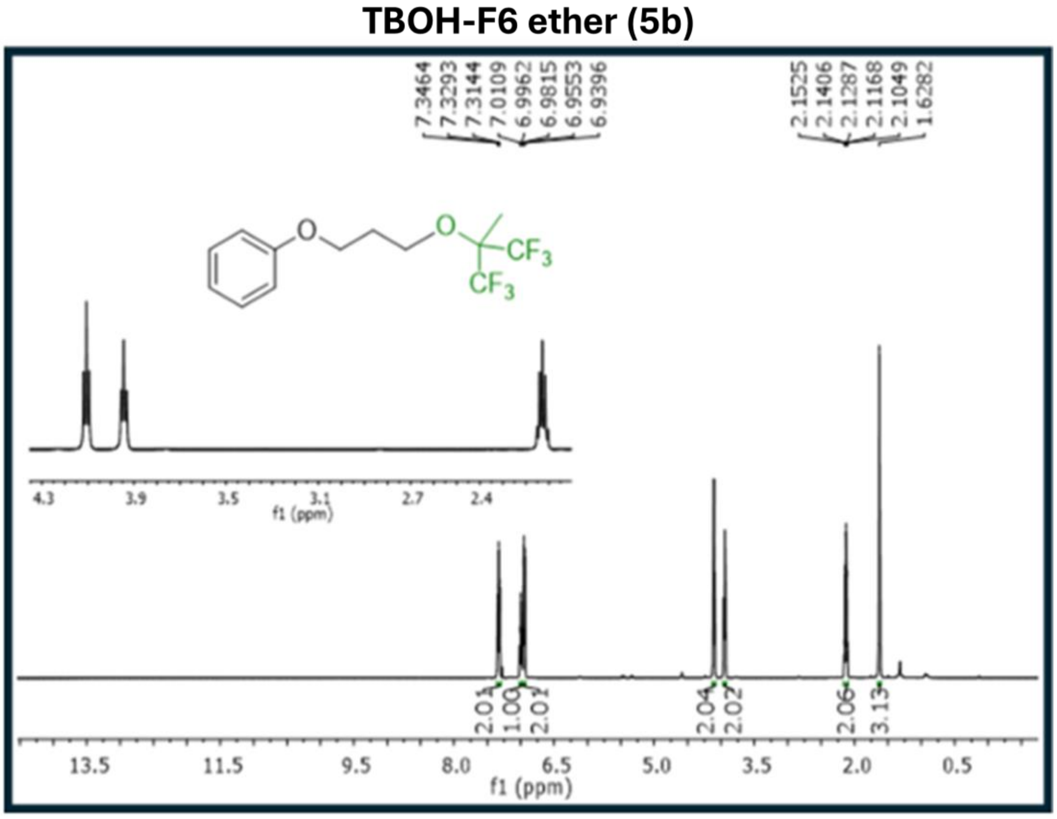

Figure S52. ^{1}H NMR ($CDCl_3$, 500 MHz) spectrum of **5b**

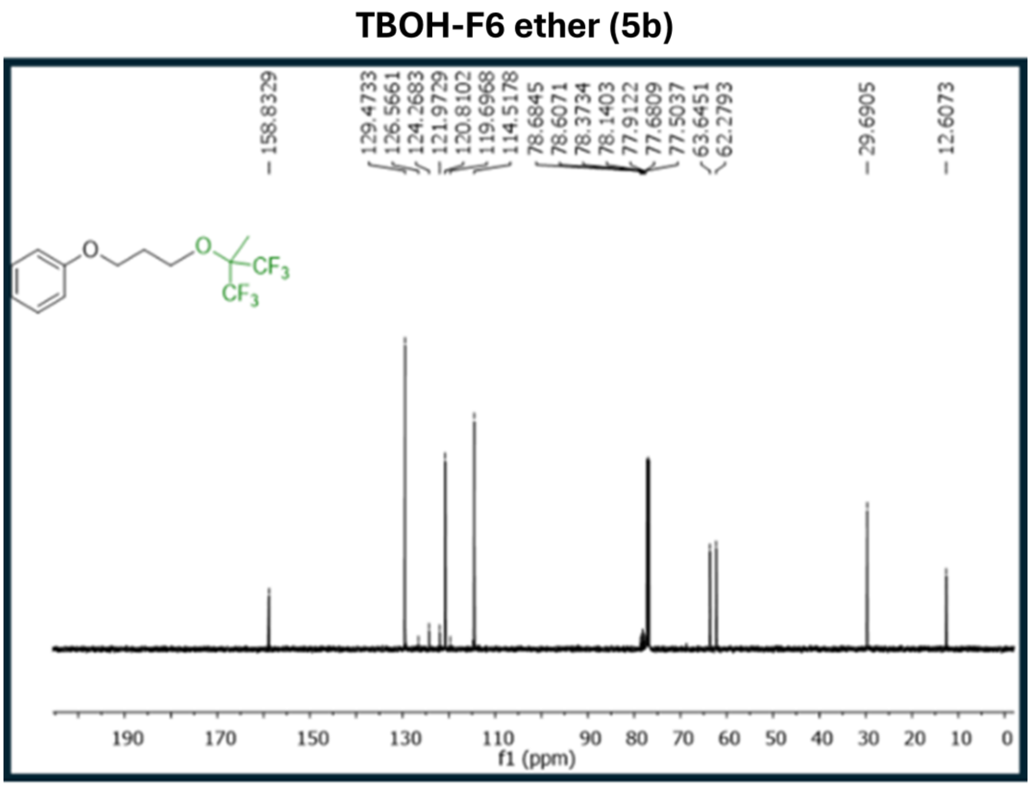

Figure S53. $^{13}C\{^{1}H\}$ NMR ($CDCl_3$, 125 MHz) spectrum of **5b**

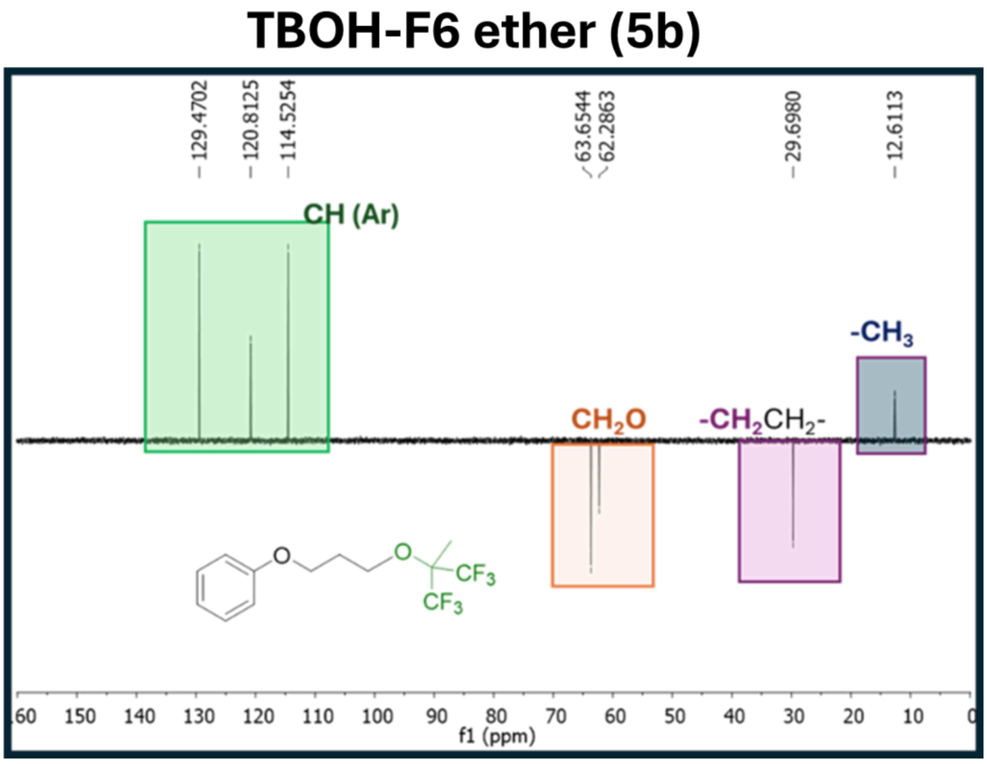

Figure S54. DEPT135 of **5b**

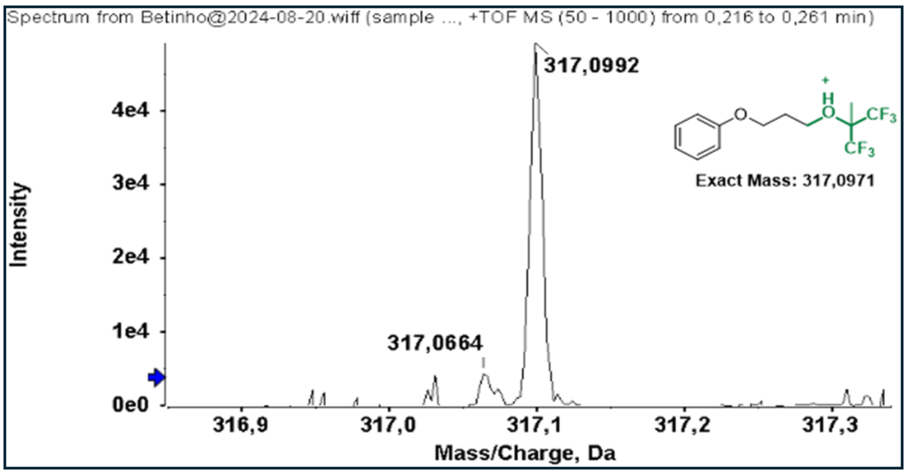

Figure S55. HRMS (ESI) *m/z*: $[M+H]^+$ Calcd for $C_{13}H_{15}F_6O_2^+$ 317.0971; Found 317.0992 expanded spectrum of **5a**

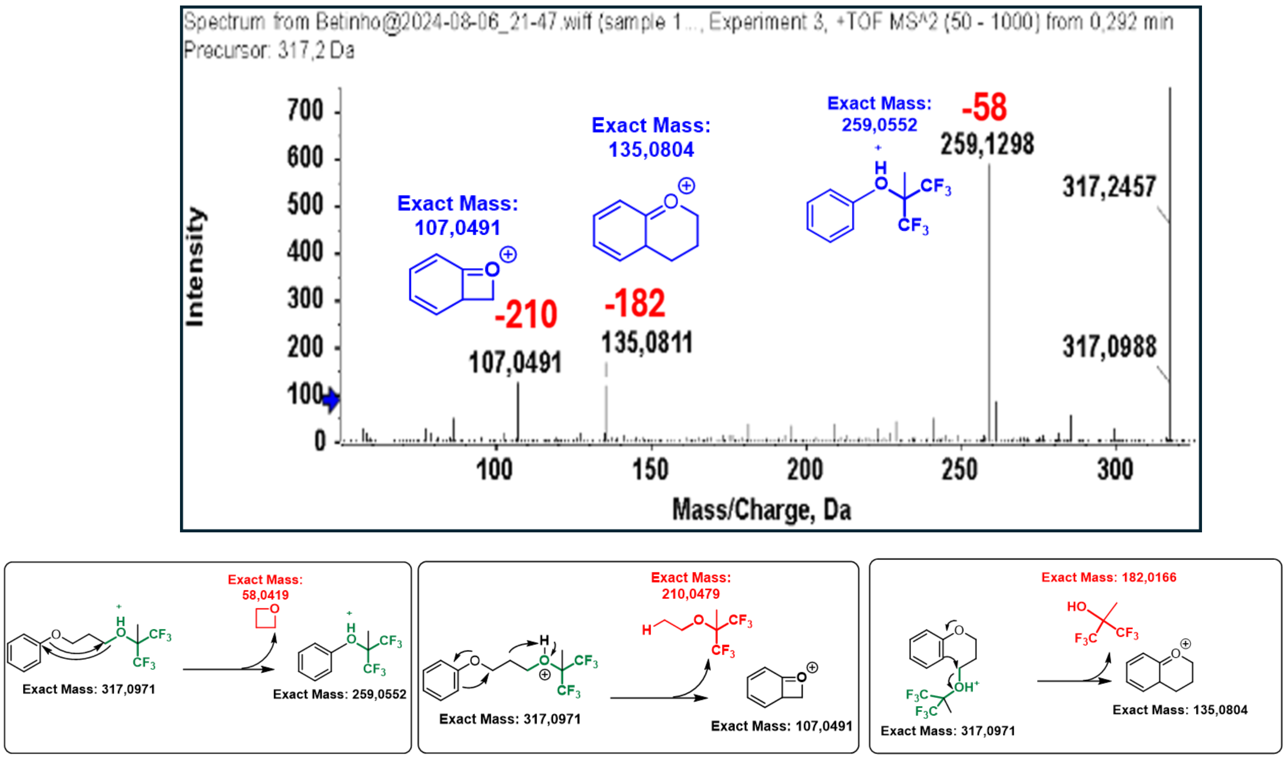

Figure S56. ESI(+)-MS/MS of the signal of *m/z* 317 and its respective fragmentation reactions (**5b**)

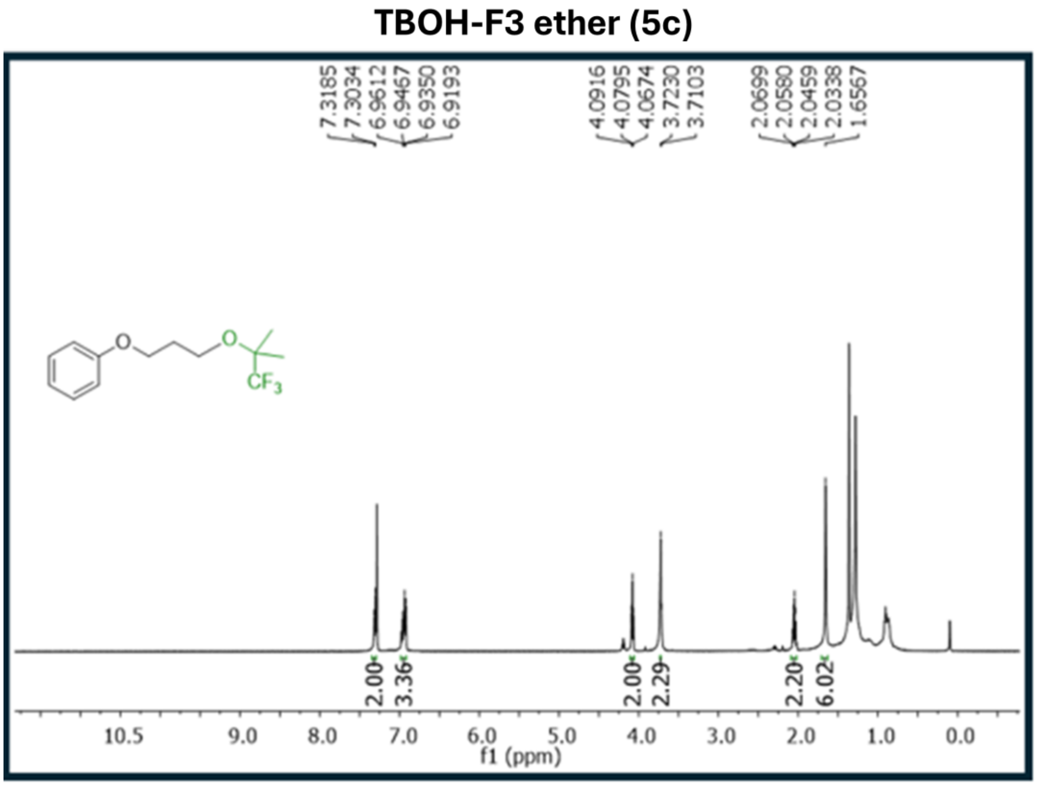

Figure S57. ^{1}H NMR ($CDCl_3$, 500 MHz) spectrum of **5c**

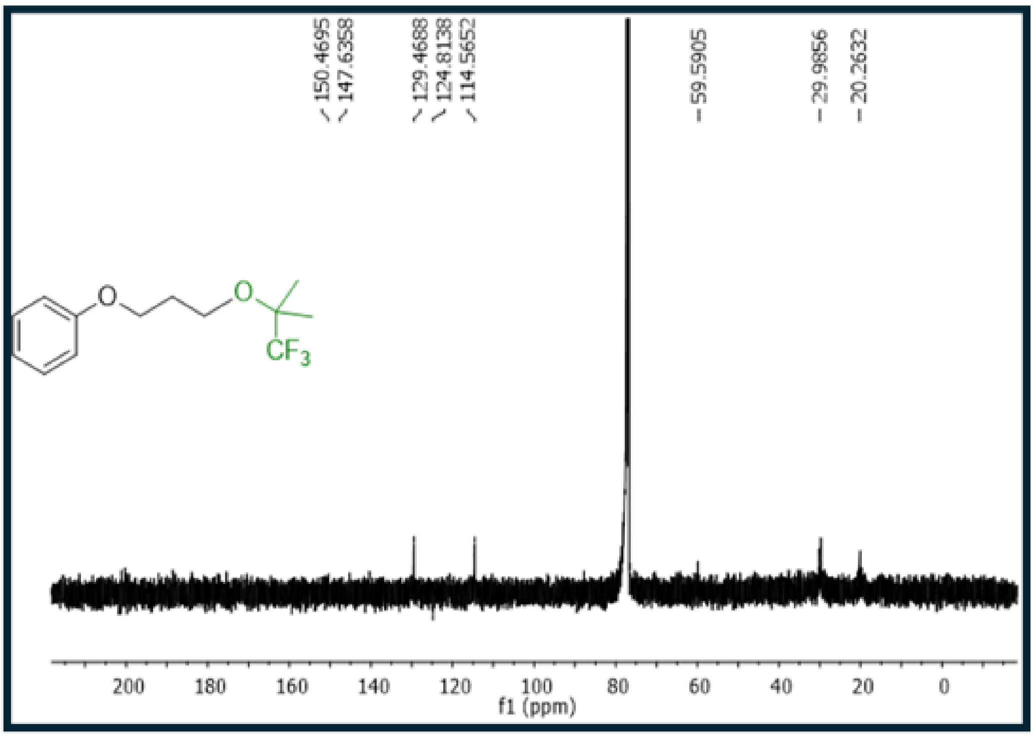

Figure S58. $^{13}C\{^{1}H\}$ NMR ($CDCl_3$, 125 MHz) spectrum of **5c**

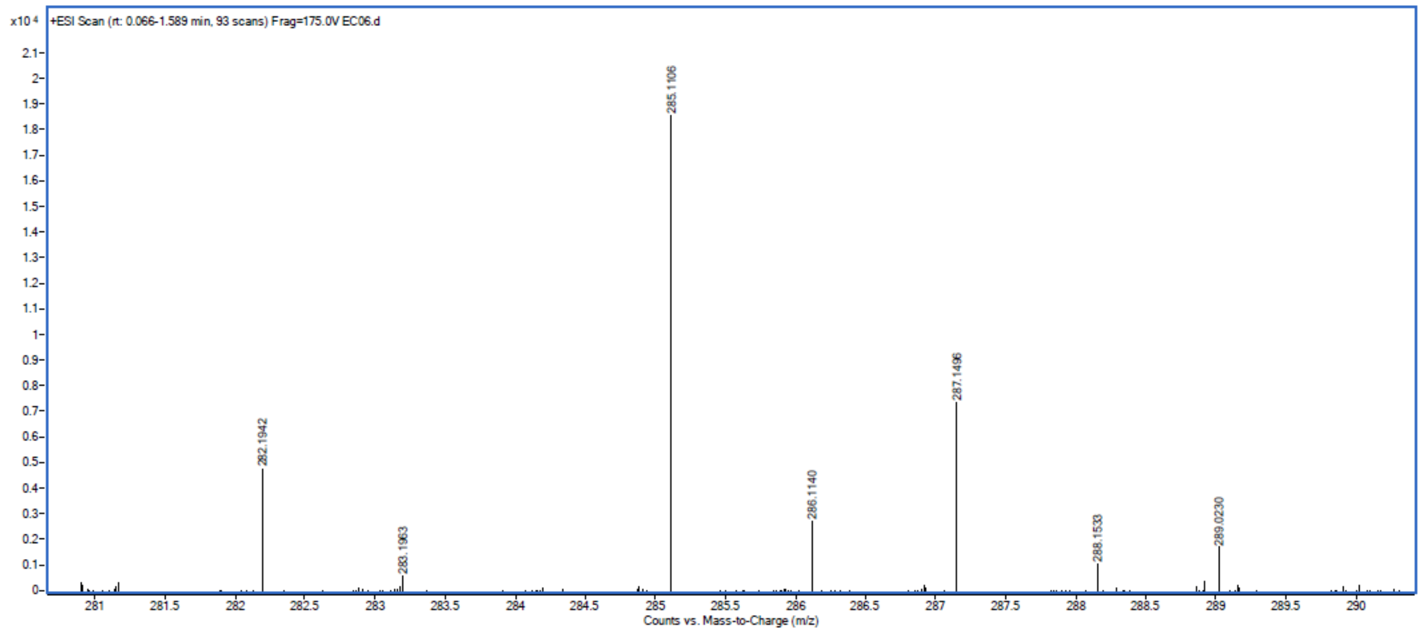

Figure S59. HRMS (ESI) *m/z*: $[M+Na]^+$ Calcd for $C_{13}H_{13}F_6O_2^+$ 285.1073; Found 285.1106 expanded spectrum of **5c**

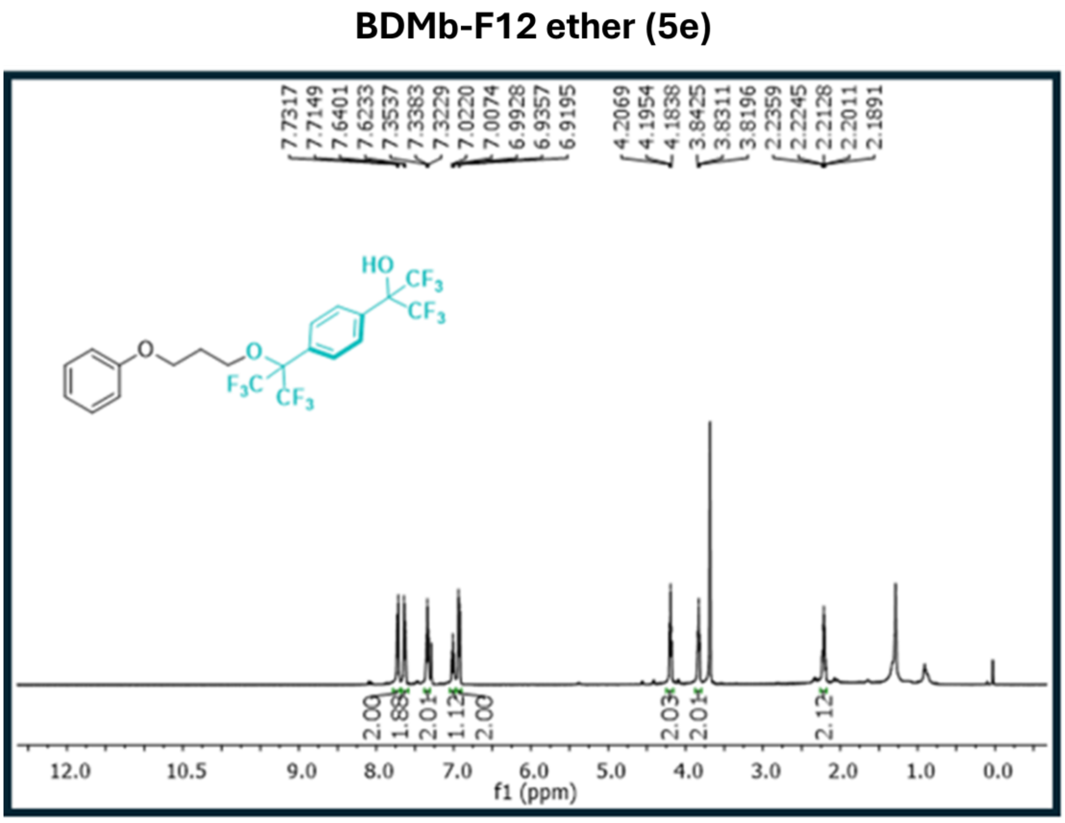

Figure S60. ^1H NMR ($CDCl_3$, 500 MHz) spectrum of **5e**

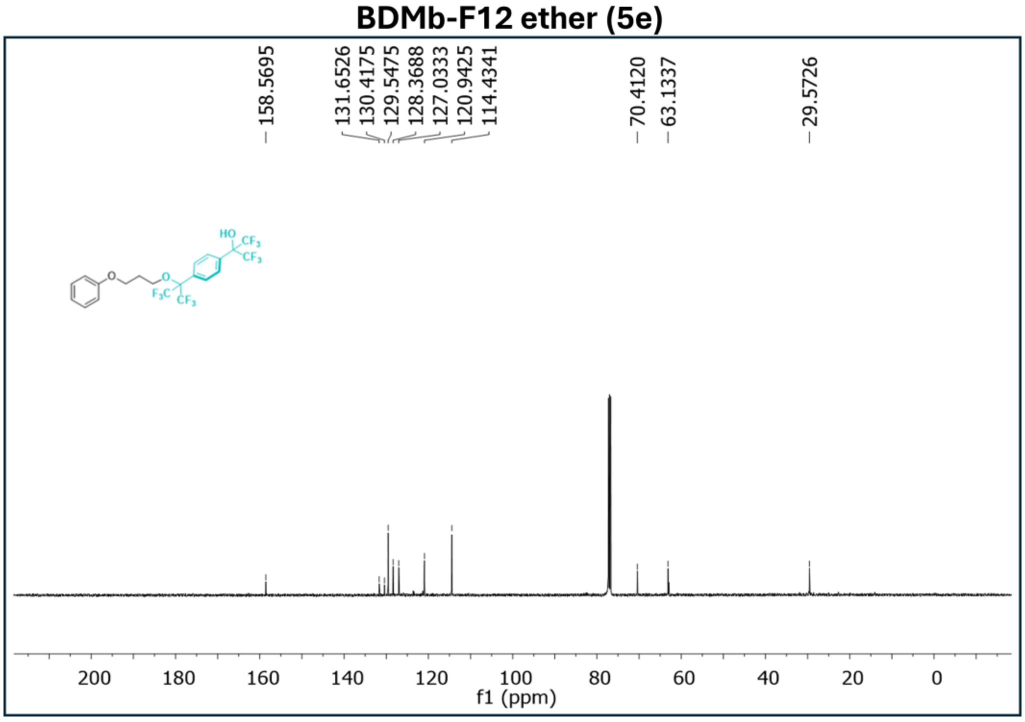

Figure S61. $^{13}C\{^{1}H\}$ NMR ($CDCl_3$, 125 MHz) spectrum of **5e**

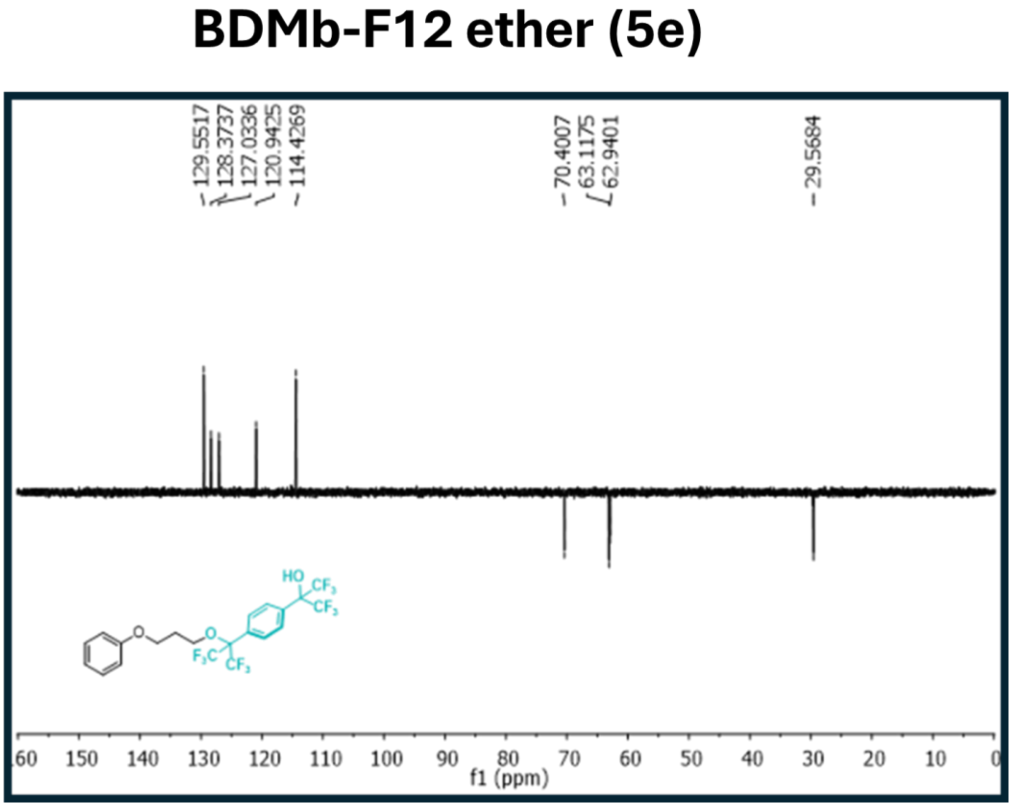

Figure S62. DEPT135 of **5e**

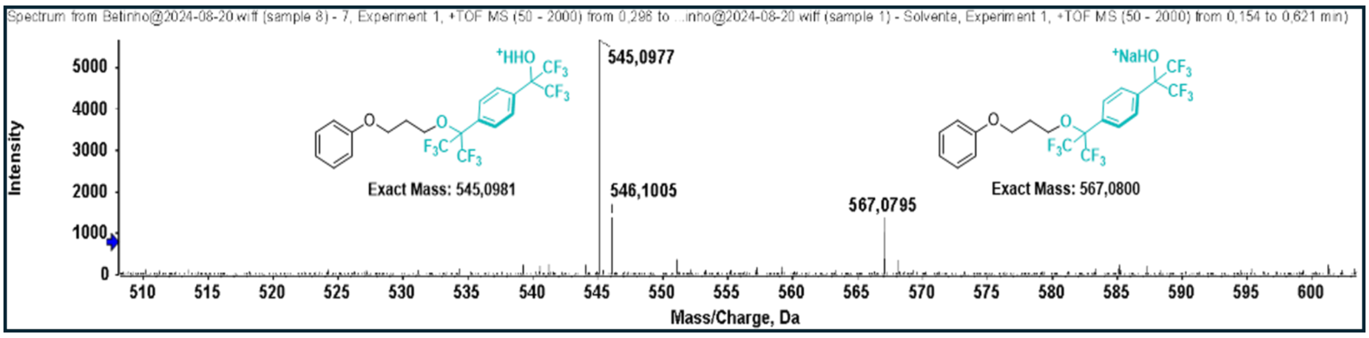

Figure S63. HRMS (ESI) *m/z*: $[M+H]^+$ Calcd for $C_{21}H_{17}F_{12}O_3^+$ 545.0981; Found 545.0977 and $[M+Na]^+$ Calcd for $C_{21}H_{16}F_{12}NaO_3^+$ 567.0800; Found 567.0795 expanded spectrum of **5e**

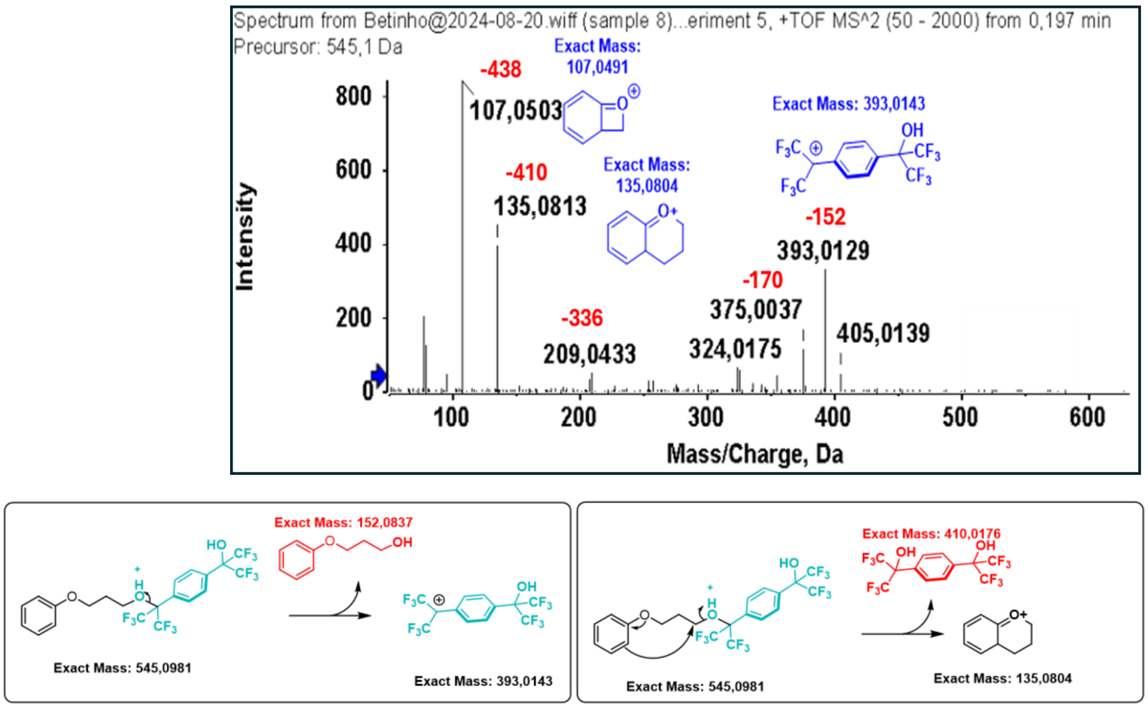

Figure S64. ESI(+)-MS/MS of the signal of *m/z* 545 and its respective fragmentation reactions (**5e**)

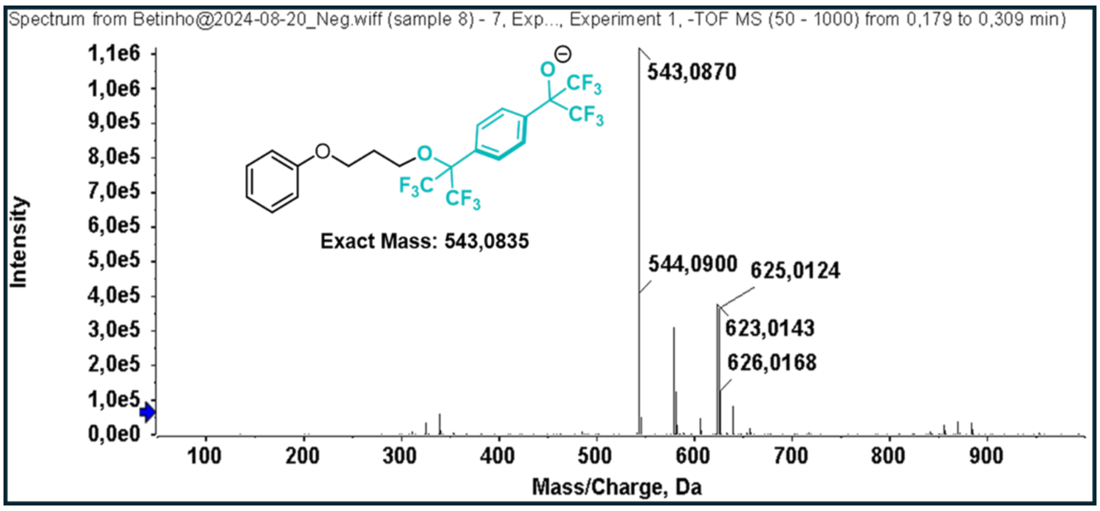

Figure S65. HRMS (ESI) *m/z*: $[M]^-$ Calcd for $C_{21}H_{15}F_{12}O_3^-$ 543.0835; Found 543.0870 spectrum of **5e**

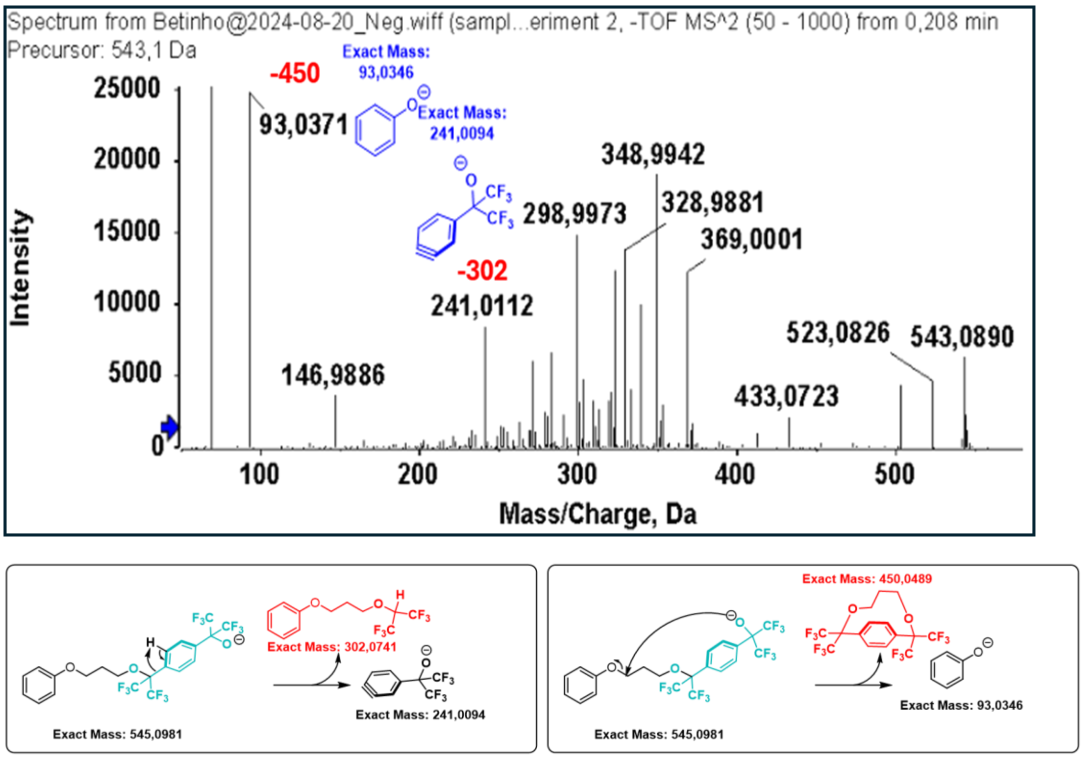

Figure S66. ESI(-)-MS/MS of the signal of *m/z* 545 and its respective fragmentation reactions (**5e**)

3. Behavior of alkyl/aryl alcohols towards the selectivity: basicity x nucleophilicity of fluorine anion

3.3.1. Hydrolysis reaction

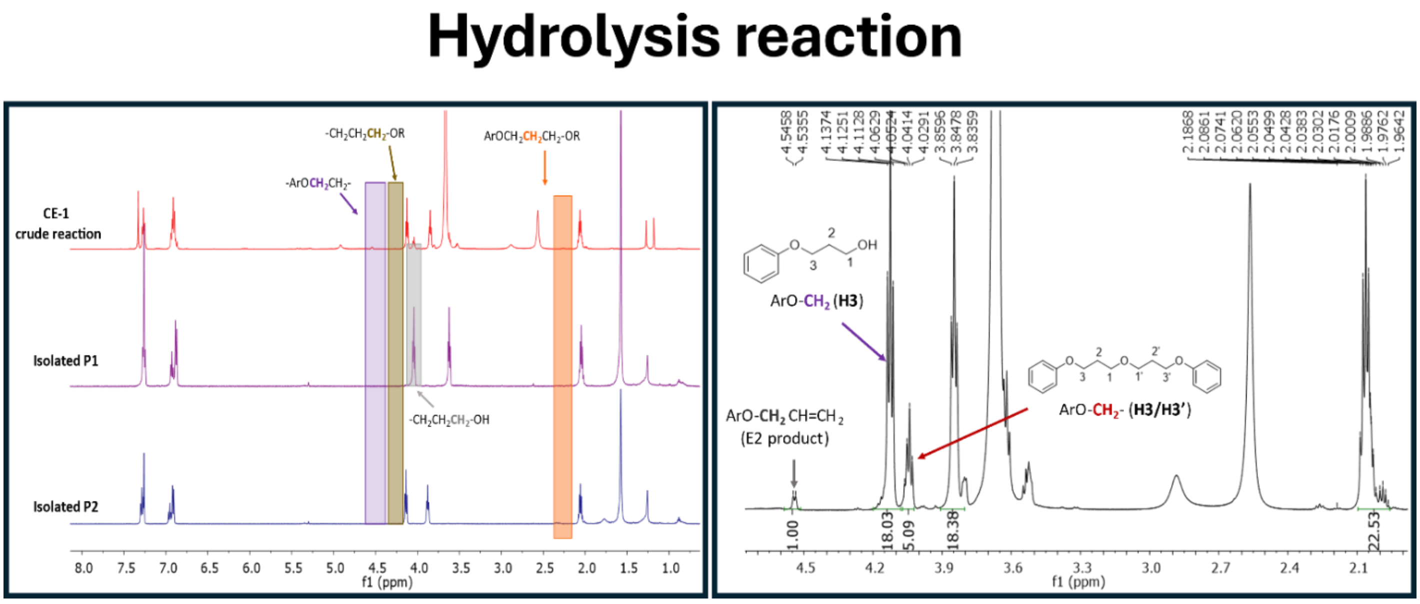

Comment: In control experiment 1, the source of KOH as both a basic and nucleophilic species and in a similar way to KF, led to the formation of the S_N2 product, the **4** alcohol, mainly. Only a small portion of the dimerization product (**6**) and E2 are observed, suggesting that hydroxide ions are not enough strong to deprotonate the hydroxyl group of **4** nor the Hβ of **1**. After 12 hours of reaction, the conversion of the starting material was total, leading to the hydrolysis product (**4**-P2) in 84% yield against 11 % for the dimer (P1-**6**) and 5% of **3** (measured by ^{1}H NMR).

Figure S67. ^{1}H NMR ($CDCl_3$, 500 MHz) spectra of the crude reaction, isolated products (P1-**6** and P2-**4**)

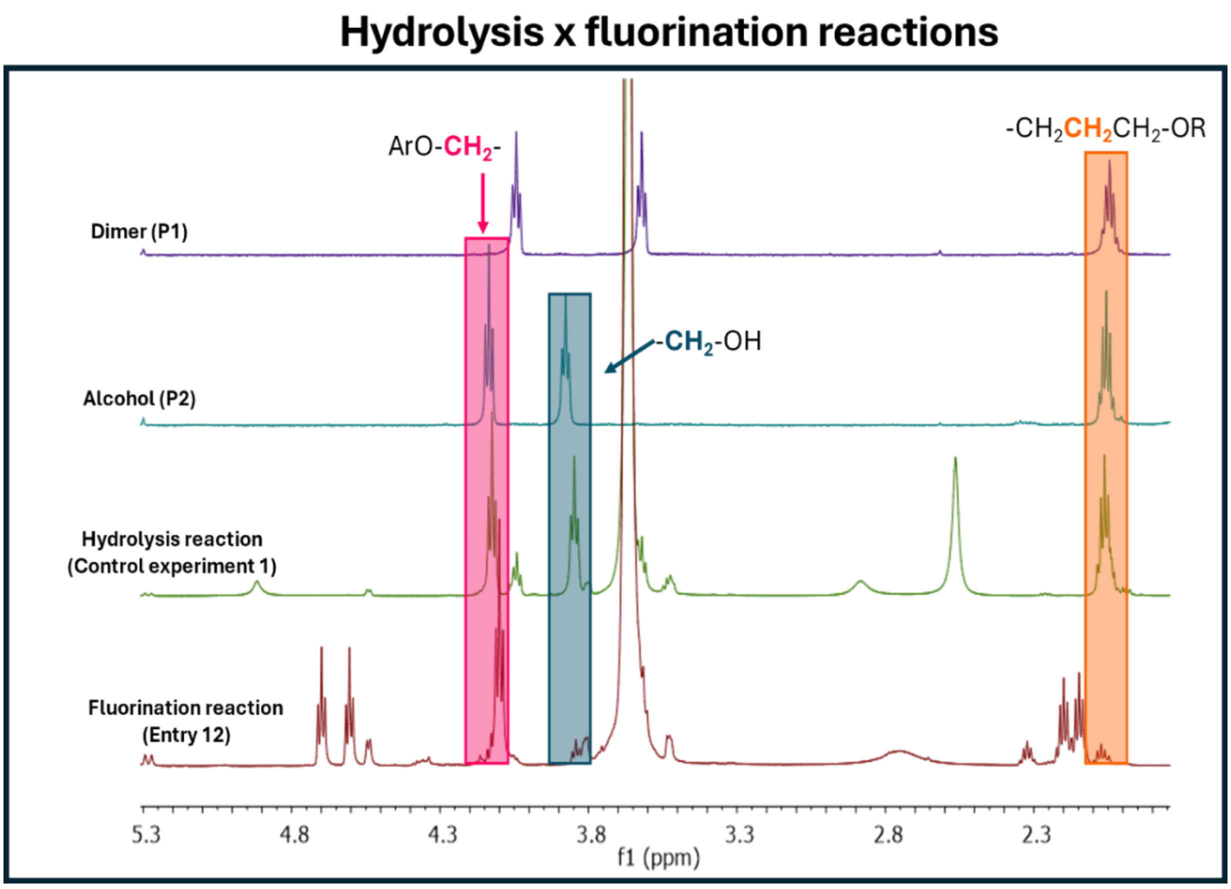

Comment: When we compare the behavior of both KOH and KF, under the same PTC conditions, a small portion of the hydrolysis product is generated under fluorination conditions is observed. This can be attributed to the presence of water in the reaction system, absorbed from the atmosphere, since the reaction is conducted under normal conditions. In addition, these reaction conditions did not produce the dimer, obtained in the control experiment 1.

Figure S68. ^{1}H NMR ($CDCl_3$, 500 MHz) spectra of the isolated C-O bond formation products (P1-**6** and P2-**4**), the crude reactions of both hydrolysis and fluorination (Table S1, Entry 12)

3.3.2. HFIP

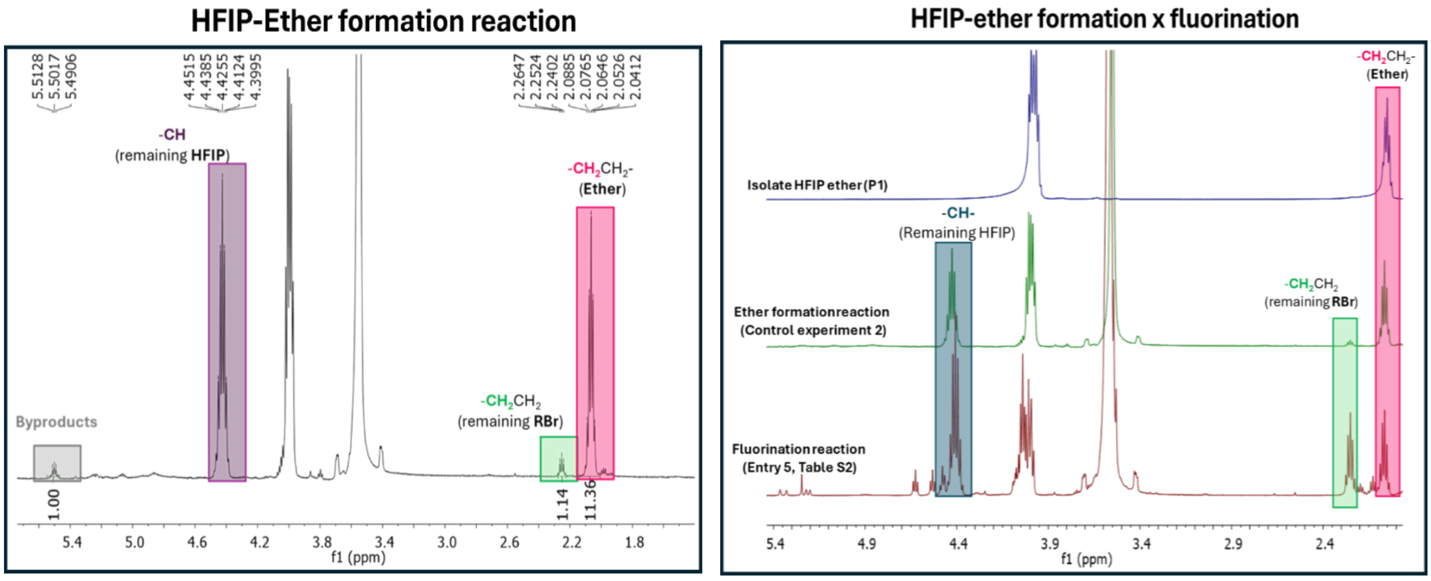

Comment: For HFIP as a reagent, the use of a weak base was enough to forward the alkoxide and then react with primary alkyl bromide with 92% conversion, after 24h. When we compare the basicity of both K_2CO_3 and KF, we observe that they led to the ether. It suggests that they are strong enough bases to deprotonate HFIP and make it a nucleophilic species for S_N2 justifying the competition with the fluoride nucleophile.

Figure S69. ^{1}H NMR ($CDCl_3$, 500 MHz) spectra of crude reaction of Control experiment **2** (left), and isolated ether (P1-**5a**), crude reactions of ether formation with HFIP (Control experiment 2) and fluorination (Table S2, Entry 5)

3.3.3. TBOH-F6

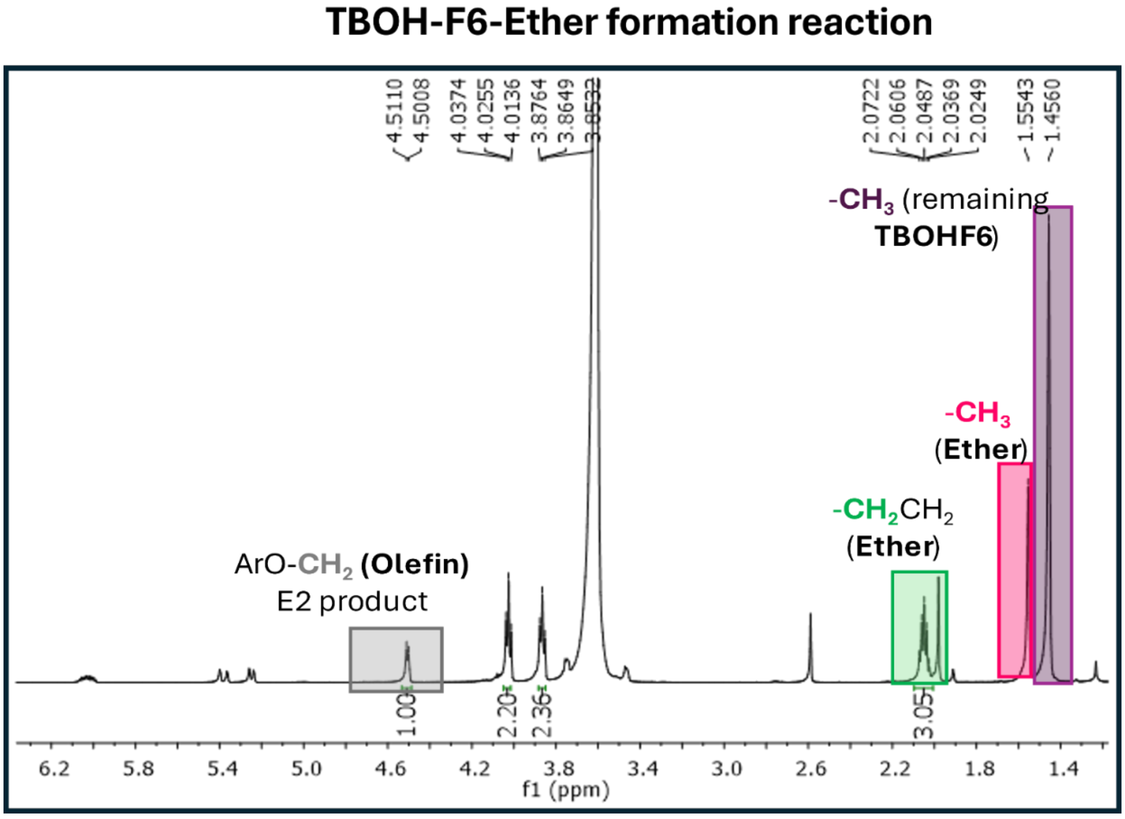

Comment: In the crude reaction, the complete consumption of the starting material in the respective disposal products (E2) and ether (S_N2, via alkoxide) is observed in a ratio of 1:3. With this result, it is suggested that the use of a weak base is strong enough to generate nucleophilic alkoxide. In the presence of KF, a stronger base, a competition of fluorination with ether is observed.

Figure S70. ^{1}H NMR spectrum ($CDCl_3$, 500 MHz) of crude reaction of control experiment **3**

TBOH-F6-ether formation x hydrolysis x fluorination

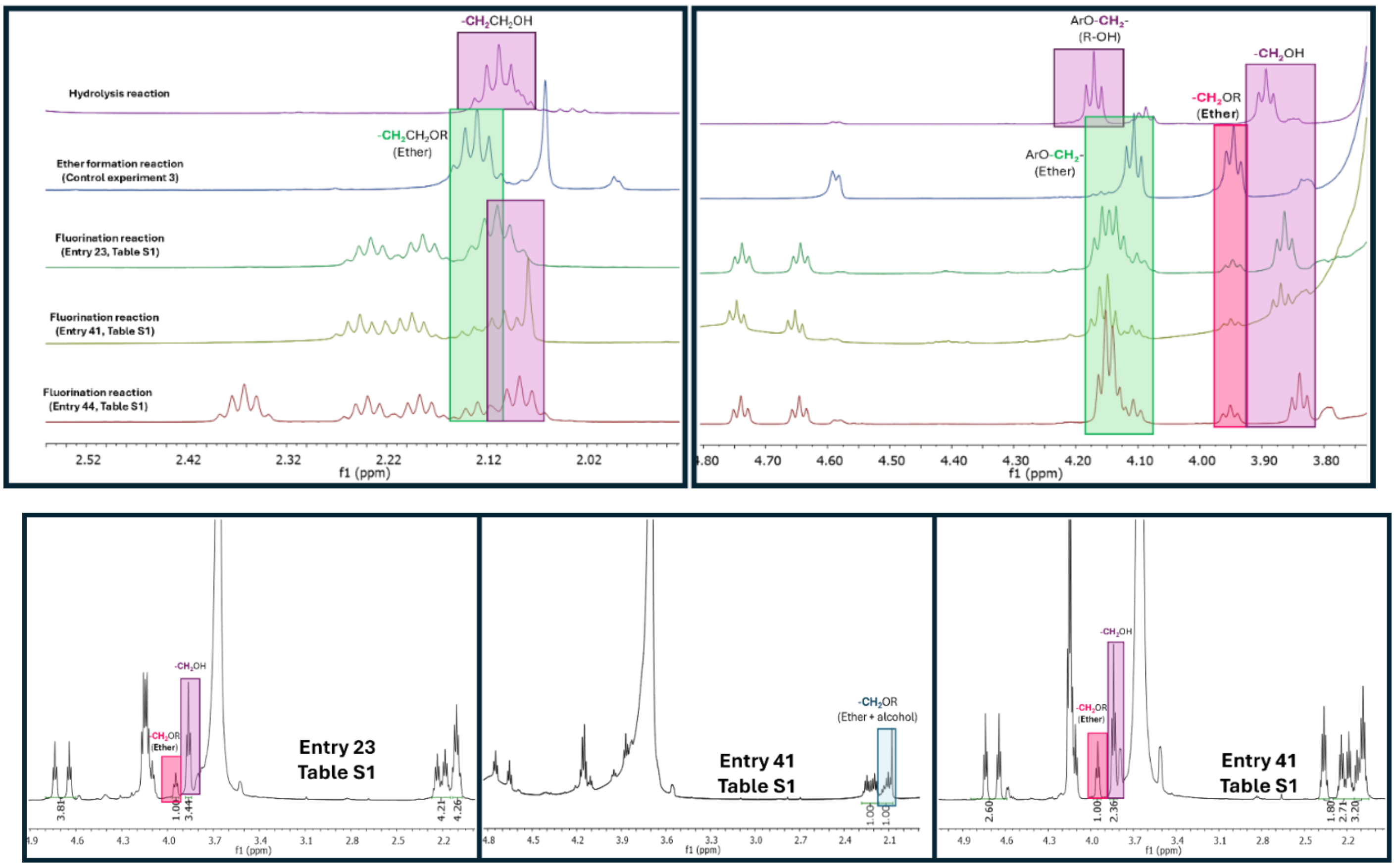

Comment: The combination 18C6/hexafluorinated alcohol (1:3), led to 48% formation of C-O products. Among them, the hydrolysis product was observed in relation to TBOH-F6 ether (3:1 ratio). The combination 18C6/alcohol (2:3) led to only 17%, however, it was not possible to determine the alcohol/ether ratio. A possible explanation could be a significant increase in fluorination kinetics, to the detriment of hydrolysis, promoted by the catalytic system employed. Finally, the 18C6/alcohol combination (2:6, Entry S44) inhibited not only the fluorination reaction, but also led to the formation of 41% of C-O products. However, a higher amount of ether is observed when compared to the PTC:alcohol 1:3 system (Entry S23), in a ratio of 2:1.

Figure S71. Selectivity data and analysis of C-O and C-F formations using TBOH-F6

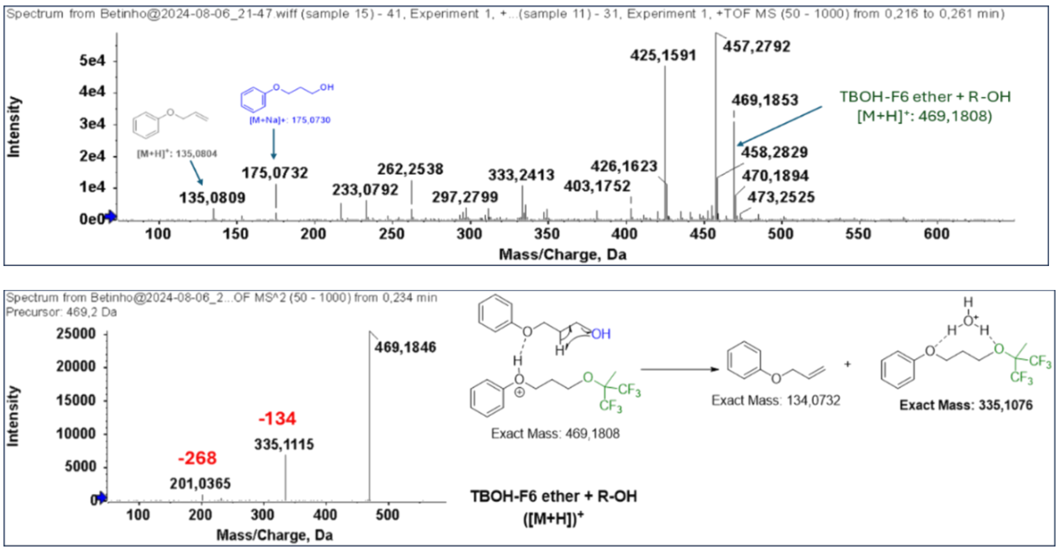

Comment: Some species were identified via HRMS (+TOF), with solvent and 18C6 subtraction. In addition, the MS/MS fragmentations of the signals, *m/z* 135.0 and 175.1, signals of **3** and **4**, respectively. The TBOH-F6 ether was found as an adduct ($[M+H+ROH]^+$)

Figure S72. HRMS spectra of fluorination reaction (Entry 41, Table S1) and ESI(+)-MS/MS of *m/z* 469

3.3.4. TBOH-F3-ether reaction formation

TBOH-F3-Ether formation reaction

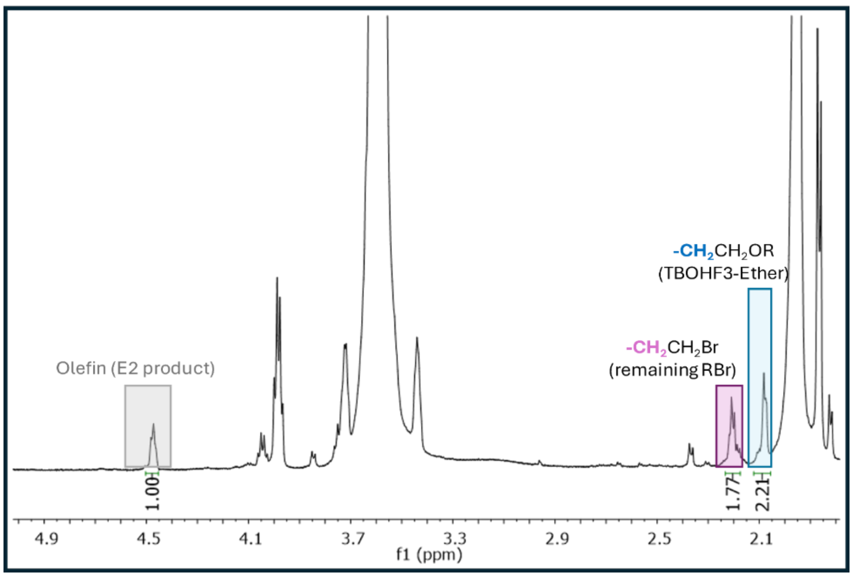

Comment: Unlike TBOH-F6, the trifluorinated bulky alcohol was shown to be less nucleophilic under the same catalytic conditions. After 24 hours, 40% ether, 20% E2 and 40% bromide remaining. These results corroborate the data obtained on the selectivity of fluorination reactions, since even in a strongly basic medium, the alkoxide was not nucleophilic enough. In addition, the fluoride anion is not basic enough to generate the nucleophile in question

Figure S73. ^1H NMR spectrum of crude reaction of control experiment **4** ($CDCl_3$, 500 MHz)

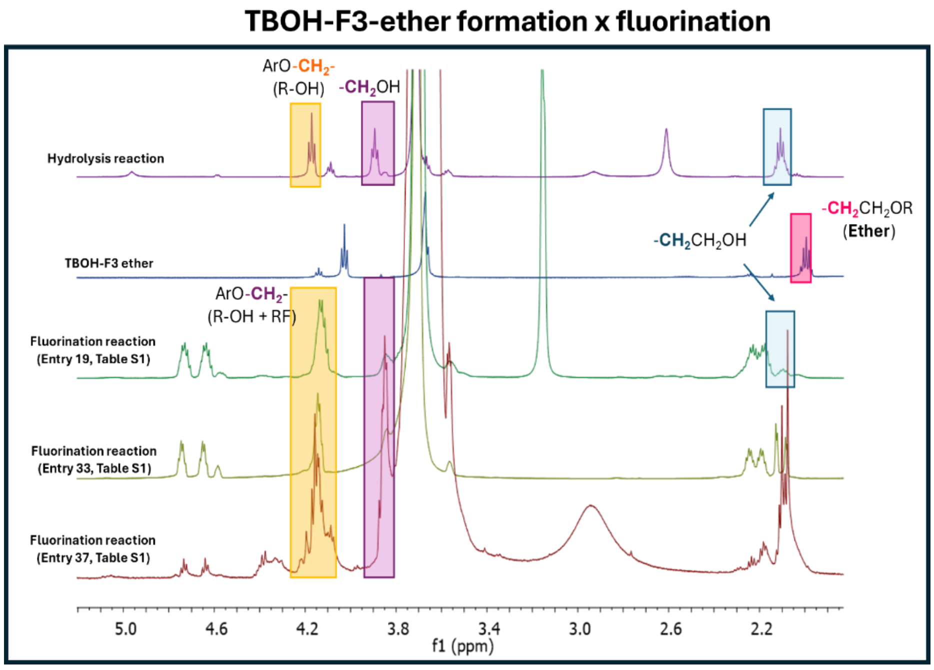

Comment: A little amount of hydrolysis product is observed, under fluorination conditions, however, fluorine ion was not strong enough to generate the nucleophile and it corroborates the best results about TBOH-F3 as catalyst.

Figure S74. Selectivity data and analysis of C-O and C-F formations using TBOH-F3

3.3.5. TBOHt-F6-ether reaction formation

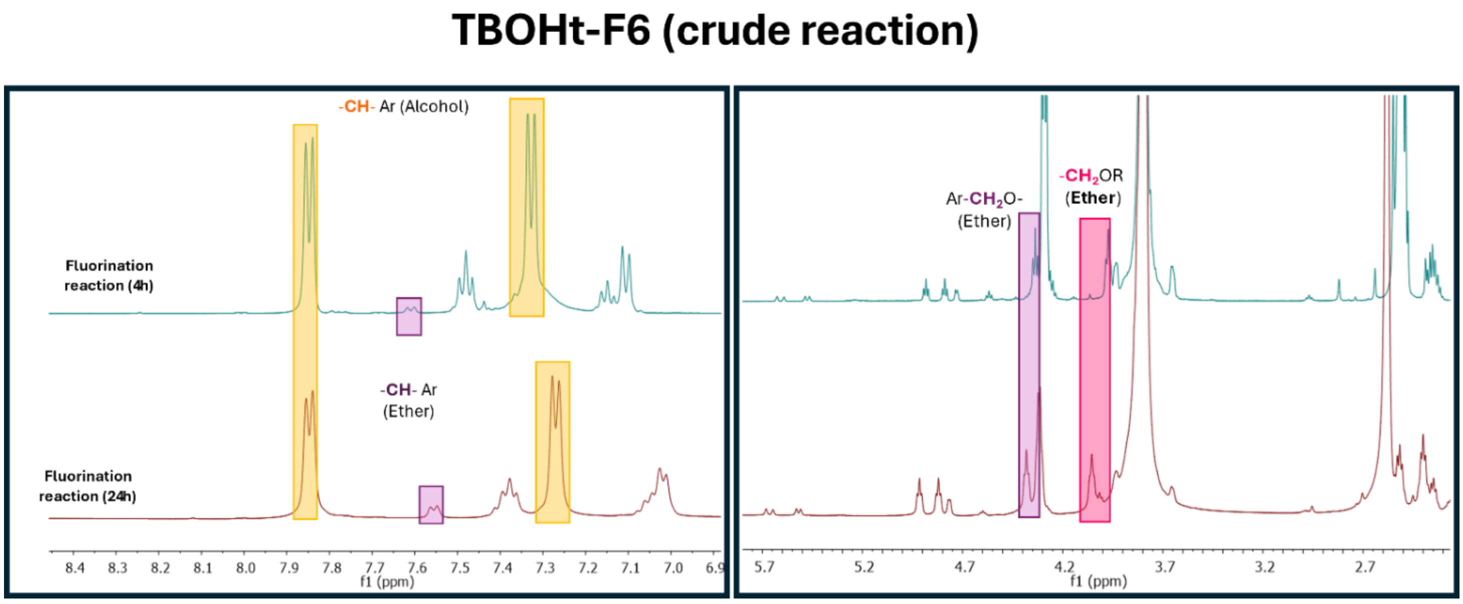

Figure S75. ^{1}H NMR spectra of reaction crude of TBOHt-F6 (1 eq) in the presence of 1 eq of 18C6 after 4h (left) and 24h (right)

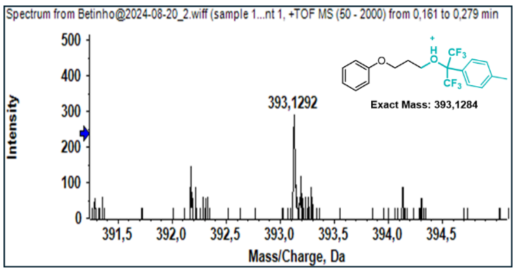

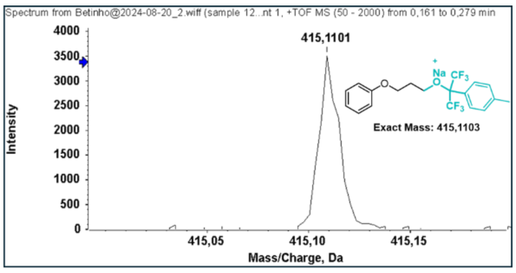

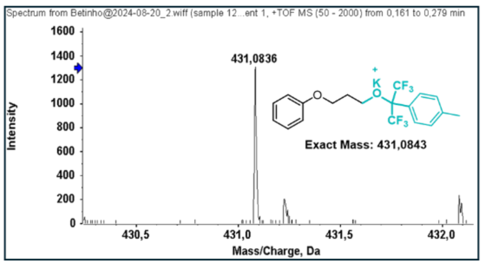

Figure S76. Expanded HRMS spectra of fluorination crude reaction relative to the compound **5d -** HRMS (ESI) *m/z*: $[M+H]^+$ $C_{19}H_{19}F_6O_2^+$ Calcd for 393.1284; Found 393.1292; $[M+Na]^+$ $C_{19}H_{18}NaF_6O_2^+$ Calcd for 415.1103; Found 415.1101; $[M+K]^+$ $C_{19}H_{18}KF_6O_2^+$ Calcd for 431.0843; Found 431.0836

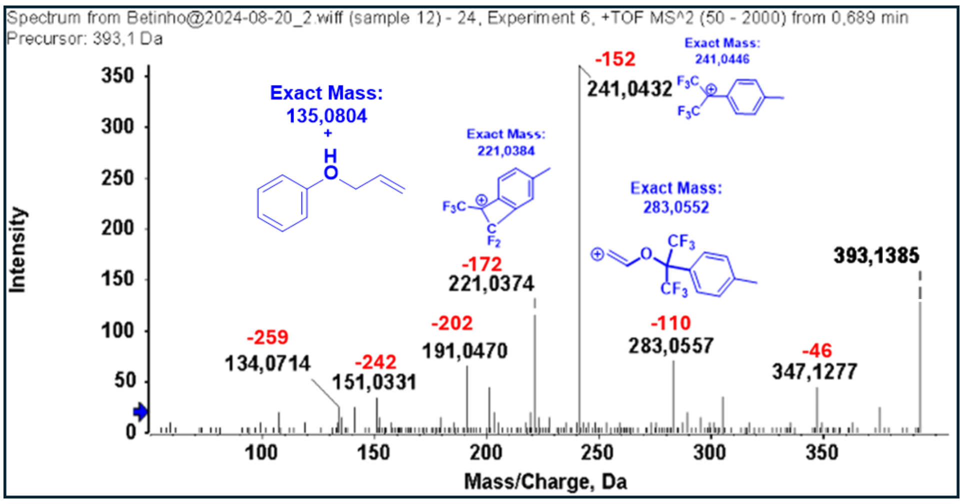

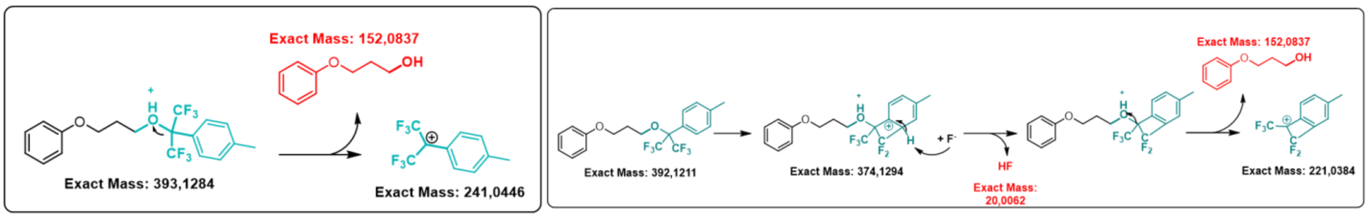

Figure S77. ESI(+)-MS/MS of the signal of *m/z* 393 and its respective fragmentation reactions (**5d**)

3.3.6. BDMb-F12 alcohol

BDMb-F12 (crude reactions)

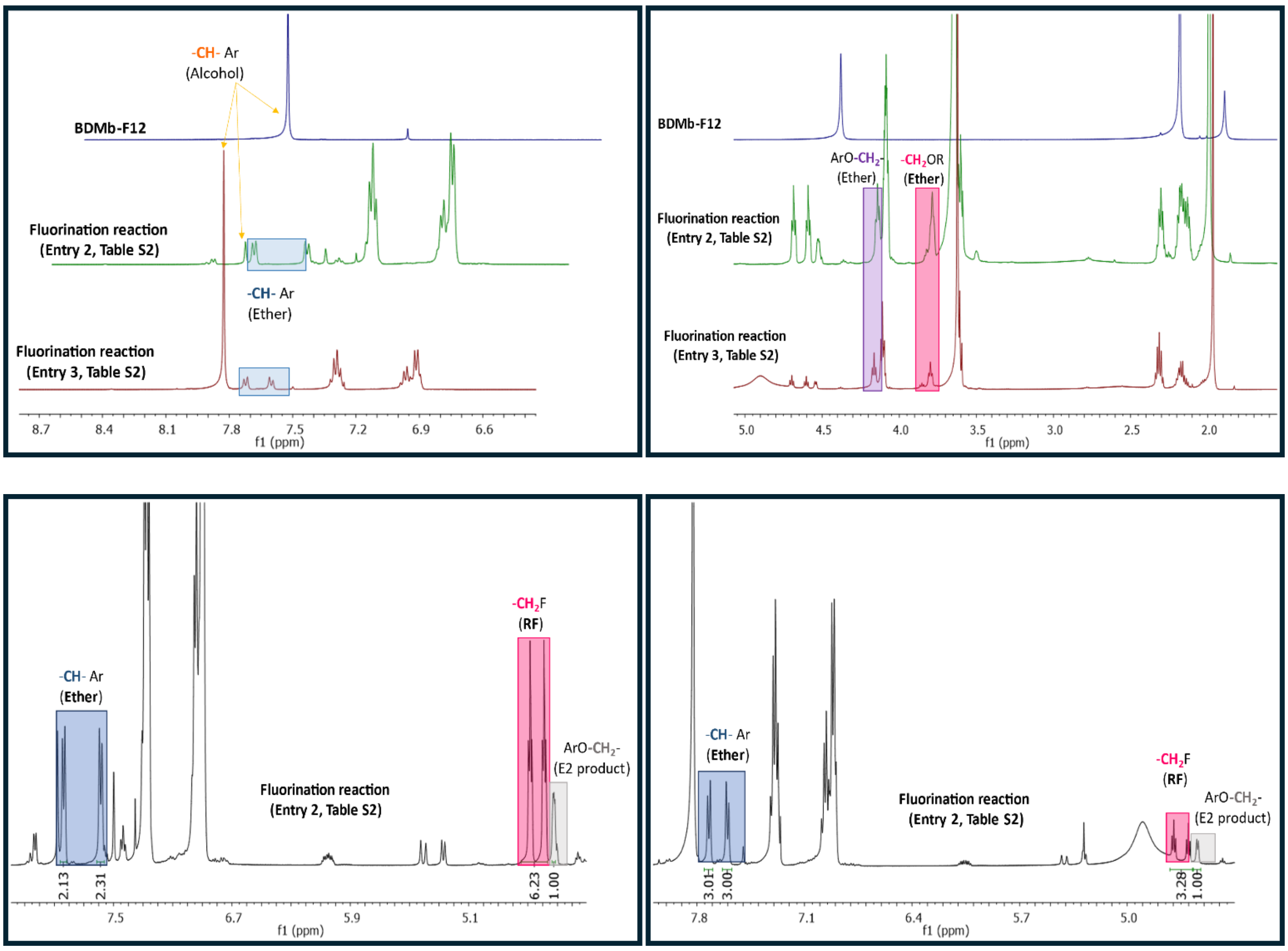

Comment: The effect of BDMb-F12 alcohol at different concentrations (1 and 3 eq) showed that both the react and selectivity of the fluorination reaction depend on its concentration. When comparing both systems, observed a greater suppression of E2 when alcohol is in smaller quantities, showing a significant effec chemoselectivity, in a ratio of 6:1 S_N2:E2. On the other hand, this effect is reversed when alcohol is preser higher concentrations, where the S_N2:E2 ratio is 3:1. Regarding the reactivity, via S_N2 reactions, we obser that the fluorination product is more favored in a smaller amount of alcohol, in a ratio of 3:1 in relation to formation of ether, while in the presence of 3 eq of it, it was 1:1.

Figure S78. ^{1}H NMR ($CDCl_3$, 500 MHz) spectra of crude reactions of TBOHt-F6 (1 and 3 eq) under fluorination conditions and selectivity data.

4. Table of Theoretical Calculations

Table S3: Calculated thermodynamic properties.[a]

Process	ωB97M-V[b]	ΔGn[c]	ΔΔGsolv[d]	ΔGg[e]	ΔGsol[f]
**TBOH-F6 alcohol**					
KF(s) -> KF(g)					48.3
KBr(s) -> KBr(g)					42.0
KF(g) + 18C6 -> KF-18C6	-36.16	9.14	-16.48	-27.02	-43.50
KBr(g) + 18C6 -> KBr-18C6	-42.02	10.24	-13.62	-31.78	-45.41
KF-18C6 + RBr -> TS1-18C6	-2.08	11.28	11.85	9.20	21.06
KF-18C6 + RBr -> TS2-18C6-c1	4.67	7.66	10.74	12.33	23.07
KF-18C6 + RBr -> TS2-18C6-c2	1.86	7.69	11.61	9.55	21.16
KF-18C6 + RBr -> KBr-18C6 + RF	-24.08	1.65	2.60	-22.43	-19.83
KF-18C6 + RBr -> KBr-18C6 + HF + PE2	-8.33	-9.96	-0.64	-18.29	-18.92
KF-18C6 + TOHa -> KF-18C6-TOHb	-36.48	9.93	18.02	-26.55	-8.54
KBr-18C6 + TOHa -> KBr-18C6-TOHb	-17.79	9.82	10.51	-7.97	2.53
KF-18C6-TOHb + RBr -> TS1-18C6-TOHb	12.43	14.08	2.26	26.51	28.77
KF-18C6-TOHb + RBr -> TS2-18C6-TOHb	24.92	8.89	-0.04	33.81	33.77
KF-18C6-TOHb + RBr -> KBr-18C6-TOHb + RF	-5.39	1.54	-4.91	-3.85	-8.76
KF-18C6-TOHb + RBr -> KBr-18C6-TOHb + HF + PE2	10.36	-10.07	-8.15	0.29	-7.85
**TBOH-F3 alcohol**					
	ωB97M-V[b]	ΔGn[c]	ΔΔGsolv[d]	ΔGg[e]	ΔGsol[f]
KF-18C6 + TOH -> KF-18C6-TOH	-27.44	9.70	12.92	-17.74	-4.82
KBr-18C6 + TOH -> KBr-18C6-TOH	-17.23	9.88	9.81	-7.35	2.46
KF-18C6-TOH + RBr -> TS1-18C6-TOH	4.52	13.25	8.94	17.77	26.71
KF-18C6-TOH + RBr -> TS2-18C6-TOH	15.58	9.55	6.30	25.13	31.43
KF-18C6-TOHb + RBr -> KBr-18C6-TOHb + RF	-13.87	1.83	-0.50	-12.04	-12.54
KF-18C6-TOHb + RBr -> KBr-18C6-TOHb + HF + PE2	1.88	-9.78	-3.74	-7.90	-11.64

a – Units in kcal mol^{-1}, 298 K, 1 mol L^{-1} standard state. Geometry optimization at CPCM/X3LYP/ma-def2-SVP level. b – calculation with the ma-def2-TZVPP basis set. c – Vibrational, rotational, and translational contribution to the free energy. d – Solvent effect calculated by the CPCM method. e – Gas phase free energy. f – Solution phase free energy.

Table S4: Electronic energies in Hartree of the optimized structures.[a]

TBOH-F6 data		
	ωB97M-V	Number of Imaginary frequencies
KF	-699.810199	0
KBr	-3173.995485	0
RBr	-2998.794499	0
RF	-524.638244	0
PE2	-424.14994	0
TOHa	-829.2993373	0
18C6	-922.989727	0
KF-18C6	-1622.857547	0
KBr-18C6	-4097.052179	0
HF	-100.463195	0
TS1-18C6	-4621.655353	1
TS2-18C6-c2	-4621.649077	2
KF-18C6-TOHb	-2452.215025	1
KBr-18C6-TOHb	-4926.379872	1
TS1-18C6-TOHb	-5450.989721	2
TS2-18C6-TOHb	-5450.969806	2

TBOH-F3 data		
	ωB97M-V	Number of Imaginary frequencies
TOH	-531.481248	0
KF-18C6-TOH	-2154.382525	1
KBr-18C6-TOH	-4628.560885	0
TS1-18C6-TOH	-5153.169828	2
TS2-18C6-TOH	-5153.152203	2

a – Electronic energies obtained at ωB97M-V level with the def2-TZVPP basis set (ma-def2-TZVPP for O, F, Br) on the CPCM/X3LYP/def2-SVP optimized structures. The additional imaginary frequencies in the transition states and minima were very low (close to 10 cm^{-1}) and difficult to eliminate. For the calculation of the free energies in these cases, these frequencies were included as real frequencies of 10 cm^{-1} for more accurate calculations.

5. References

(1) Iwasaki, T.; Fukuoka, A.; Yokoyama, W.; Min, X.; Hisaki, I.; Yang, T.; Ehara, M.; Kuniyasu, H.; Kambe, N. Nickel-Catalyzed Coupling Reaction of Alkyl Halides with Aryl Grignard Reagents in the Presence of 1,3-Butadiene: Mechanistic Studies of Four-Component Coupling and Competing Cross-Coupling Reactions. *Chem. Sci.* **2018**, *9* (8), 2195–2211. https://doi.org/10.1039/C7SC04675H.

(2) Atack, T. C.; Lecker, R. M.; Cook, S. P. Iron-Catalyzed Borylation of Alkyl Electrophiles. *J. Am. Chem. Soc.* **2014**, *136* (27), 9521–9523. https://doi.org/10.1021/ja505199u.
